# The genetic architecture of fibromyalgia across 2.5 million individuals

**DOI:** 10.1038/s41591-026-04492-6

**Published:** 2026-07-28

**Authors:** Isabel Kerrebijn, Gyda Bjornsdottir, Keon Arbabi, Lea Urpa, Hele Haapaniemi, Gudmar Thorleifsson, Lilja Stefansdottir, Stephan Frangakis, Jesse Valliere, Lovemore Kunorozva, Erik Abner, Caleb Ji, Markus Kangur, Bitten Aagaard, Henning Bliddal, Søren Brunak, Mie T. Bruun, Maria Didriksen, Christian Erikstrup, Sarah Finer, Arni J. Geirsson, Daniel F. Gudbjartsson, Thomas F. Hansen, David van Heel, Ingileif Jonsdottir, Stacey Knight, Kirk U. Knowlton, Christina Mikkelsen, Lincoln D. Nadauld, Thorunn A. Olafsdottir, Sisse R. Ostrowski, Ole B. V. Pedersen, Saedis Saevarsdottir, Astros T. Skuladottir, Erik Sørensen, Hreinn Stefansson, Patrick Sulem, Olafur A. Sveinsson, Gudny E. Thorlacius, Unnur Thorsteinsdottir, Henrik Ullum, Arnor Vikingsson, Thomas M. Werge, Chronic Pain Genomics Consortium, Isabel Kerrebijn, Gyda Bjornsdottir, Stephan Frangakis, Erik Abner, Thomas F. Hansen, Frances M. K. Williams, Chad M. Brummett, Thorgeir E. Thorgeirsson, Nasa Sinnott-Armstrong, Hanna M. Ollila, Michael Wainberg, FinnGen, Lea Urpa, Hele Haapaniemi, Hanna M. Ollila, DBDS Genomic Consortium, Bitten Aagaard, Henning Bliddal, Søren Brunak, Mie T. Bruun, Maria Didriksen, Christian Erikstrup, Thomas F. Hansen, Christina Mikkelsen, Sisse R. Ostrowski, Ole B. V. Pedersen, Henrik Ullum, Thomas M. Werge, Bente Glintborg, Estonian Biobank Research Team, Erik Abner, Genes & Health Research Team, Sarah Finer, David van Heel, Richa Saxena, Kari Stefansson, Chad M. Brummett, Bente Glintborg, Daniel J. Clauw, Thorgeir E. Thorgeirsson, Frances M. K. Williams, Nasa Sinnott-Armstrong, Hanna M. Ollila, Michael Wainberg

**Affiliations:** 1 Lunenfeld-Tanenbaum Research Institute, Mount Sinai Hospital, Toronto, Ontario Canada; 2 Institute of Medical Science, University of Toronto, Toronto, Ontario Canada; 3 Amgen deCODE Genetics, Reykjavik, Iceland; 4 Krembil Centre for Neuroinformatics, Centre for Addiction and Mental Health, Toronto, Ontario Canada; 5 Stanley Center for Psychiatric Research, Broad Institute, Cambridge, MA USA; 6 Center for Genomic Medicine, Massachusetts General Hospital, Boston, MA USA; 7 Institute for Molecular Medicine Finland (FIMM), HiLIFE, University of Helsinki, Helsinki, Finland; 8 Department of Anesthesiology, University of Michigan Medical School, Ann Arbor, MI USA; 9 Molecular and Population Genetics Program, Broad Institute, Cambridge, MA USA; 10 Division of Sleep and Circadian Disorders, Brigham and Women’s Hospital, Boston, MA USA; 11 Institute of Genomics, Estonian Genome Center, University of Tartu, Tartu, Estonia; 12 Department of Computer Science, University of Toronto, Toronto, Ontario Canada; 13 Department of Clinical Immunology, Aalborg University Hospital, Aalborg, Denmark; 14 The Parker Institute, Copenhagen University Hospital Bispebjerg Frederiksberg, Copenhagen, Denmark; 15 Department of Public Health, University of Copenhagen, Copenhagen, Denmark; 16 Novo Nordisk Center for Protein Research, University of Copenhagen, Copenhagen, Denmark; 17 Clinical Immunology Research Unit, Department of Clinical Immunology, Odense University Hospital, Odense, Denmark; 18 Department of Clinical Immunology, Copenhagen University Hospital, Rigshospitalet, Copenhagen, Denmark; 19 Department of Clinical Immunology, Aarhus University Hospital, Aarhus, Denmark; 20 Department of Clinical Medicine, Aarhus University, Aarhus, Denmark; 21 Wolfson Institute of Population Health, Queen Mary University of London, London, UK; 22 Department of Rheumatology, Landspitali University Hospital, Reykjavik, Iceland; 23 Neurogenomics, Translational Research Centre, Copenhagen University Hospital, Glostrup, Denmark; 24 Danish Multiple Sclerosis Center, Copenhagen University Hospital, Glostrup, Denmark; 25 Danish Headache Center, Copenhagen University Hospital, Glostrup, Denmark; 26 Blizard Institute, Queen Mary University of London, London, UK; 27 Precision Healthcare University Research Institute, Queen Mary University of London, London, UK; 28 Intermountain Medical Center, Intermountain Heart Institute, Salt Lake City, UT USA; 29 Intermountain Healthcare, Saint George, UT USA; 30 School of Health Sciences, Faculty of Medicine, University of Iceland, Reykjavik, Iceland; 31 Department of Clinical Medicine, Faculty of Health and Medical Sciences, University of Copenhagen, Copenhagen, Denmark; 32 Department of Clinical Immunology, Zealand University Hospital, Koege, Denmark; 33 Department of Neurology, Landspitali University Hospital, Reykjavik, Iceland; 34 Statens Serum Institut, Copenhagen, Denmark; 35 Institute of Biological Psychiatry, Mental Health Services, Copenhagen University Hospital, Copenhagen, Denmark; 36 Department of Anesthesiology, Mass General Brigham, Harvard Medical School, Boston, MA USA; 37 Opioid Research Institute, Office for the Vice President for Research, University of Michigan, Ann Arbor, MI USA; 38 Overdose Prevention Engagement Network, Institute for Healthcare Policy and Innovation, Michigan Medicine, Ann Arbor, MI USA; 39 Copenhagen Center for Arthritis Research (COPECARE) and DANBIO, Center for Rheumatology and Spine Diseases, Rigshospitalet, Glostrup, Denmark; 40 Chronic Pain and Fatigue Research Center, Department of Anesthesiology, University of Michigan, Ann Arbor, MI USA; 41 Department of Twin Research and Genetic Epidemiology, School of Life Course Sciences, King’s College London, London, UK; 42 Herbold Computational Biology Program, Fred Hutchinson Cancer Center, Seattle, WA USA; 43 Department of Genome Sciences, University of Washington, Seattle, WA USA; 44 Brotman Baty Institute, University of Washington, Seattle, WA USA; 45 Center for Synthetic Biology, University of Washington, Seattle, WA USA; 46 Center for One Health Research, University of Washington, Seattle, WA USA; 47 Department of Psychiatry, University of Toronto, Toronto, Ontario Canada; 48 Division of Biostatistics, Dalla Lana School of Public Health, University of Toronto, Toronto, Ontario Canada

**Keywords:** Genome-wide association studies, Fibromyalgia

## Abstract

Fibromyalgia is a common and debilitating chronic pain syndrome of poorly understood etiology. Here we conduct a multi-ancestry genome-wide association study meta-analysis across 2,563,755 individuals (54,629 cases and 2,509,126 controls) from 11 cohorts, identifying 26 risk loci for fibromyalgia. The strongest association was with a coding variant in *HTT*, the causal gene for Huntington’s disease. Gene prioritization implicated the *HTT* regulator *GPR52*, as well as diverse genes with neural roles, including *DCC*, *DRD2*/*NCAM1*, *MDGA2* and *CELF4*. Fibromyalgia heritability was exclusively enriched within brain tissues and neural cell types. Fibromyalgia showed strong, positive genetic correlation with a wide range of chronic pain, psychiatric and somatic disorders, including genetic correlations above 0.7 with low back pain, post-traumatic stress disorder and irritable bowel syndrome. Despite large sex differences in fibromyalgia prevalence, the genetic architecture of fibromyalgia was nearly identical between males and females. This study provides robust genetic evidence defining fibromyalgia as a central nervous system disorder, thereby establishing a biological framework for its complex pathophysiology and extensive clinical comorbidities.

## Abstract (web summary)

A multi-ancestry genome-wide association study meta-analysis across 2,563,755 individuals identifies 26 risk loci for fibromyalgia, including *HTT*, and finds genetic correlations with chronic pain and psychiatric and somatic disorders.

## Main

Fibromyalgia is a multifaceted syndrome encompassing chronic widespread musculoskeletal pain, fatigue, unrefreshing sleep, cognitive impairment and somatic symptoms. It frequently co-occurs with other pain conditions, irritable bowel syndrome, myalgic encephalomyelitis/chronic fatigue syndrome, autoimmune and neuropsychiatric disorders, and metabolic syndrome^1^.

Fibromyalgia is considered the prototypical central sensitization or nociplastic pain syndrome^2^, whereby the central nervous system becomes sensitized to peripheral nociceptive input, leading to increased pain response to painful stimuli (hyperalgesia) and typically non-painful stimuli (allodynia)^3^. Whether fibromyalgia has an autoimmune component is a matter of long-standing debate^4^.

While fibromyalgia has a partly genetic basis^5^, attempts to find specific genetic loci have proven elusive^6–9^. We searched for fibromyalgia genetic risk factors through combined genetic analysis of over 2.5 million individuals, identifying risk loci, prioritizing causal genes, mapping heritability-enriched tissues and cell types, quantifying genetic overlap with comorbidities, and assessing inter-sex genetic consistency.

## Results

### Associations with 26 variants establish genetic underpinnings of fibromyalgia

**Fig. 1:**
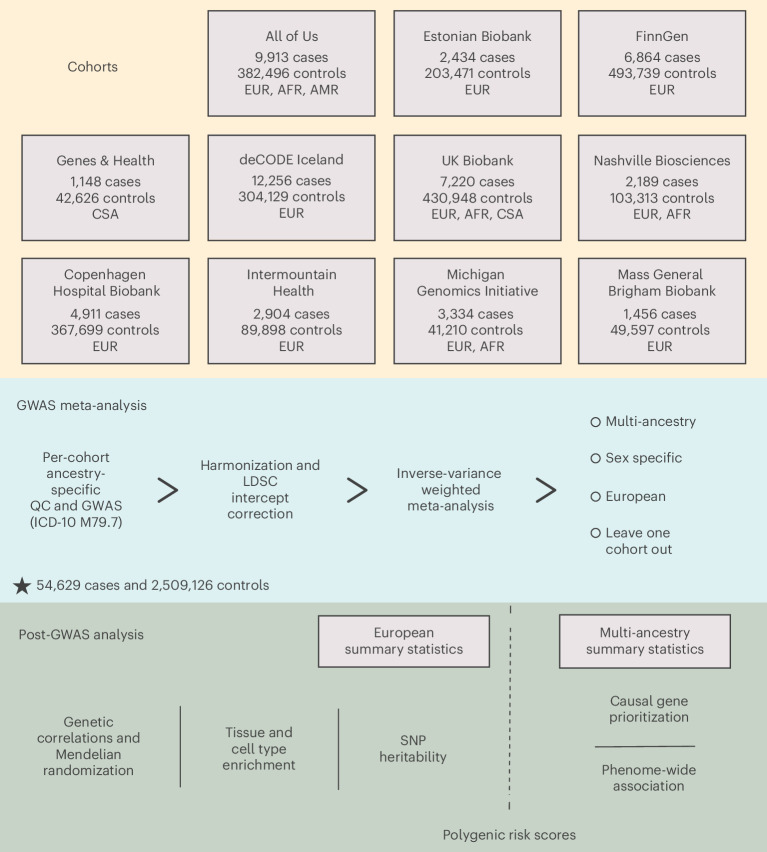
Study overview. The three panels summarize the contributing cohorts and sample sizes, the GWAS meta-analysis workflow and the downstream post-GWAS analyses performed. ‘Copenhagen Hospital Biobank’ is short for ‘Copenhagen Hospital Biobank and Danish Blood Donor Study’. EUR, European; AFR, African; AMR, admixed American.

We performed multi-ancestry genome-wide association study (GWAS) meta-analyses of fibromyalgia (International Classification of Diseases, Tenth Revision (ICD-10) code M79.7 in inpatient and/or primary care records) across 11 cohorts (Fig. 1). We performed quality control and GWAS per cohort–ancestry pair, corrected for inflation using linkage disequilibrium (LD) score regression intercepts and conducted fixed-effects inverse-variance-weighted meta-analysis across cohorts and ancestries.

**Fig. 2:**
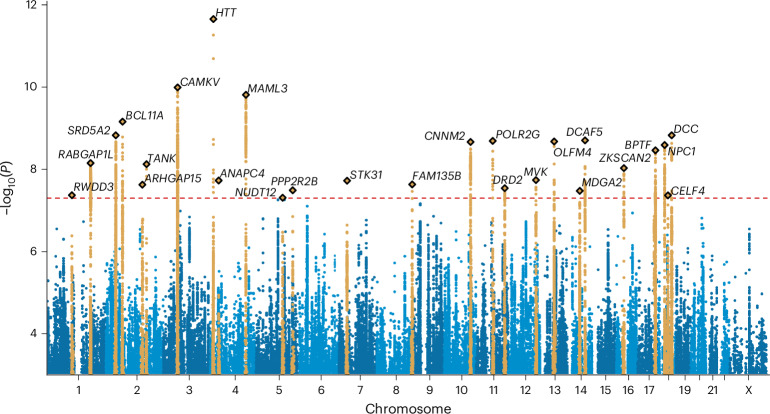
Manhattan plot of the primary meta-analysis. Association statistics were obtained from REGENIE genome-wide association analyses, with variant-level significance assessed using two-sided tests of association. Genome-wide significance was defined as *P* < 5 × 10^−8^. Gold-highlighted variants have LD *r*^2^ > 0.001 and are within 5 Mb of the lead variants (diamonds). The nearest gene to each lead variant is labeled.

Our primary meta-analysis encompassed 54,629 fibromyalgia cases (90% European) and 2,509,126 controls, totaling 2,563,755 individuals (Supplementary Table 1). Prevalence varied sixfold across cohorts, from 1.2% (Estonian Biobank) to 7.5% (Michigan Genomics Initiative), with a median prevalence of 2.5% (All of Us). We found 26 independent genome-wide-significant (*P* < 5 × 10^−8^) risk loci for fibromyalgia (Table 1 and Fig. 2), with largely consistent effect sizes across cohorts (Supplementary Fig. 1).

**Table 1:** Lead variants and candidate causal genes from the primary meta-analysis

Variant	Location (hg38)	A1	A2	A1 freq.	Odds ratio (95% CI)	*P* value	Nearest gene	FLAMES- prioritized gene	Candidate causal gene(s)
rs149109767	chr4:3,228,683	A	AGAG	8%	1.090 (1.065, 1.118)	2.2 × 10^−12^	*HTT*	*HTT*	*HTT*
rs9862795^a^	chr3:49,878,073	T	A	49%	1.045 (1.031, 1.059)	1.0 × 10^−10^	*CAMKV*	N/A	*CAMKV/MST1R*
rs809955^a^	chr4:139,953,606	A	G	37%	0.956 (0.943, 0.969)	1.5 × 10^−10^	*MAML3*	*MAML3*	*MAML3*
rs359243	chr2:60,248,374	C	T	62%	1.044 (1.030, 1.058)	6.9 × 10^−10^	*BCL11A*	*BCL11A*	*BCL11A*
rs62100765^a^	chr18:53,209,048	T	C	40%	1.043 (1.029, 1.057)	1.5 × 10^−9^	*DCC*	*DCC*	*DCC*
rs6748639	chr2:31,697,603	T	C	26%	1.048(1.032, 1.064)	1.5 × 10^−9^	*SRD5A2*	*SPAST*	*SDR5A2, SPAST*
rs71953676	chr14:69,077,365	C	CTTATTTAT	42%	1.052 (1.035, 1.069)	2.0 × 10^−9^	*DCAF5*	N/A	N/A
rs34237396	chr11:62,760,731	A	AT	54%	0.960 (0.948, 0.973)	2.0 × 10^−9^	*POLR2G*	N/A	N/A
rs1443914^a^	chr13:53,343,095	C	T	52%	0.960 (0.948, 0.973)	2.1 × 10^−9^	*OLFM4*	*PCDH8*	N/A
rs140473396	chr10:103,036,128	GAC	G	10%	0.929 (0.907, 0.952)	2.2 × 10^−9^	*CNNM2*	N/A	N/A
rs1788821	chr18:23,540,905	G	A	66%	0.958 (0.945, 0.972)	2.6 × 10^−9^	*NPC1*	*NPC1*	*NPC1*
rs34261763^a^	chr17:67,873,992	CTGGA	C	22%	1.058 (1.038, 1.077)	3.5 × 10^−9^	*BPTF*	*BPTF*	*BPTF*
rs6657341	chr1:174,306,124	A	G	45%	0.961 (0.949, 0.974)	7.1 × 10^−9^	*RABGAP1L*	N/A	*GPR52*
rs62194170	chr2:161,009,993	A	G	20%	0.952 (0.937, 0.968)	7.5 × 10^−9^	*TANK*	N/A	N/A
rs4787316^a^	chr16:25,341,698	T	C	14%	1.055 (1.036, 1.075)	9.3 × 10^−9^	*ZKSCAN2*	*ZKSCAN2*	N/A
rs3742015	chr12:109,579,320	C	T	45%	1.038 (1.025, 1.052)	1.8 × 10^−8^	*MVK*	*MMAB*	N/A
rs34811474^a^	chr4:25,407,216	A	G	21%	0.954 (0.939, 0.970)	1.9 × 10^−8^	*ANAPC4*	*ANAPC4*	N/A
rs6977550	chr7:23,865,825	G	A	50%	1.042 (1.027, 1.057)	1.9 × 10^−8^	*STK31*	*STK31*	*NPY*
rs28645908	chr8:138,519,579	A	G	9%	0.935 (0.913, 0.957)	2.3 × 10^−8^	*FAM135B*	*COL22A1*	N/A
rs10166361	chr2:143,500,256	T	C	39%	0.960 (0.946, 0.974)	2.4 × 10^−8^	*ARHGAP15*	*ARHGAP15*	*KYNU*
rs2734833	chr11:113,422,198	A	G	56%	0.962 (0.949, 0.976)	2.9 × 10^−8^	*DRD2*	*DRD2*	*DRD2, NCAM1*
rs11167957	chr5:147,056,960	T	C	60%	1.039 (1.025, 1.053)	3.2 × 10^−8^	*PPP2R2B*	*PPP2R2B*	*PPP2R2B*, *GPR151*
rs12587412^a^	chr14:46,803,220	G	T	56%	0.963 (0.950, 0.976)	3.3 × 10^−8^	*MDGA2*	*MDGA2*	*MDGA2*
rs34683740	chr1:95,843,084	C	T	7%	1.072 (1.046, 1.100)	4.3 × 10^−8^	*RWDD3*	*RP11- 147C23.1*	N/A
rs11664242^a^	chr18:37,596,198	G	A	72%	0.960 (0.946, 0.974)	4.3 × 10^−8^	*CELF4*	*CELF4*	*CELF4*
rs325506	chr5:104,676,602	G	C	58%	0.964 (0.951, 0.977)	4.9 × 10^−8^	*NUDT12*	N/A	N/A

Variant-level association significance was assessed using two-sided tests of association, with genome-wide significance defined as *P* < 5 × 10^−8^.

freq., frequency; hg38, human genome build 38; N/A, no gene prioritized.

**Fig. 3:**
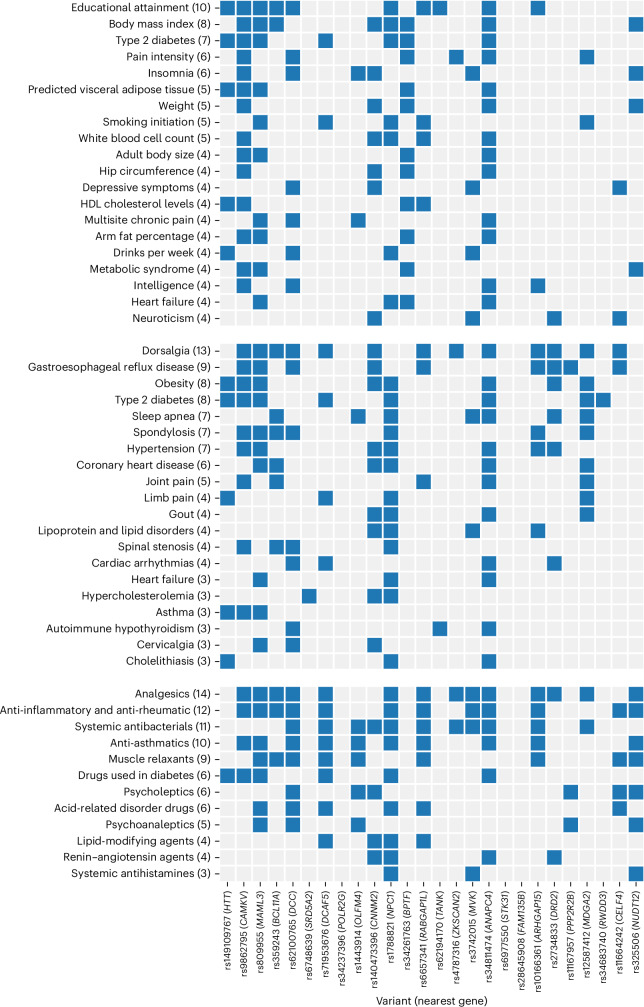
Phenome-wide associations of lead variants. Each of the 26 lead variants from the primary meta-analysis was tested for overlap with genome-wide significant variants from the GWAS Catalog, either with the lead variant itself or variants with *r*^2^ > 0.8 (top). The 26 lead variants were also tested for association with 330 diseases in a meta-analysis of the Million Veteran Program, FinnGen and the UK Biobank (middle), and 124 drug classes in FinnGen (bottom), applying Bonferroni correction across the number of variants and diseases or drug classes tested. The number of variants (out of 26) significantly associated with each disease or drug is listed in brackets; for brevity, only diseases or drugs associated with at least 10% of the lead variants (that is, ≥3 of 26) are shown, or 15% (that is, ≥4 of 26) for the GWAS Catalog analysis, with full results listed in Supplementary Tables 4–6. Only lead variants with at least one significant phenome-wide association are shown.

^a^Variants or variants in high LD have previously been associated with pain phenotypes in the GWAS Catalog (Supplementary Table 4 and Fig. 3 (top)).

**Extended Data Fig. 1:**
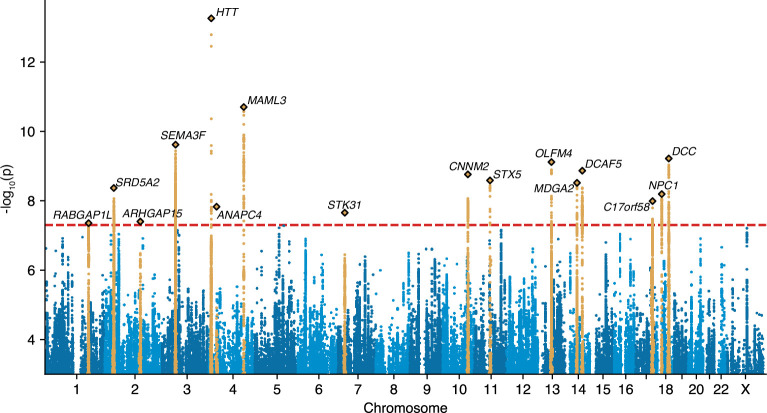
Manhattan plot of the European-only meta-analysis. Association statistics were obtained from REGENIE genome-wide association analyses, with variant-level significance assessed using two-sided tests of association. Genome-wide significance was defined as p < 5 × 10^−8^. Gold-highlighted variants have linkage disequilibrium r^2^ > 0.001 and are within 5 megabases of the lead variants (diamonds). The nearest gene to each lead variant is labeled.

**Extended Data Fig. 2:**
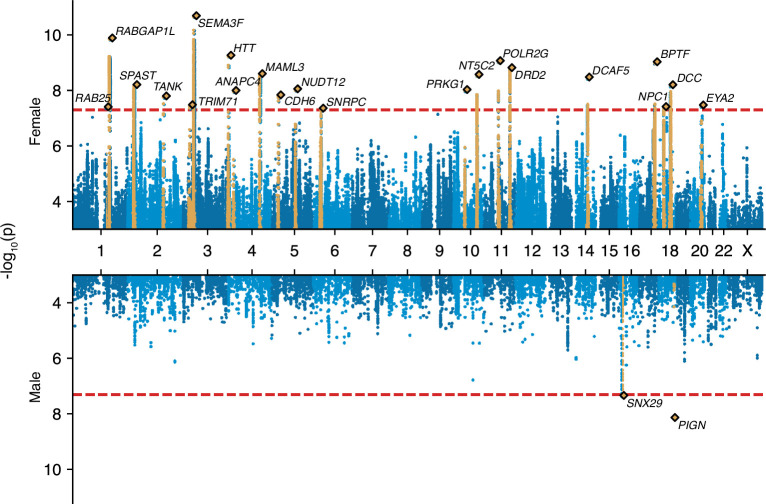
Miami plot of the multi-ancestry female-only and male-only meta-analyses. Association statistics were obtained from REGENIE genome-wide association analyses, with variant-level significance assessed using two-sided tests of association. Genome-wide significance was defined as p < 5 × 10^−8^. Gold-highlighted variants have linkage disequilibrium r^2^ > 0.001 and are within 5 megabases of the lead variants (diamonds). Note that males are European only, due to low male case counts in non-European ancestries (see Methods).

**Extended Data Fig. 3:**
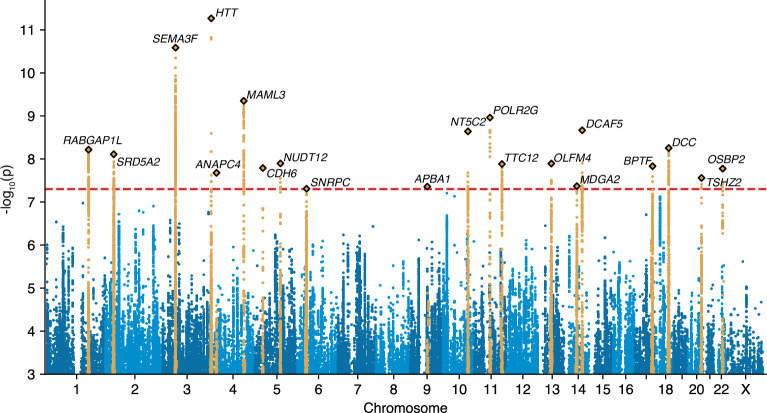
Manhattan plot of the European-only, female-only meta-analysis. Association statistics were obtained from REGENIE genome-wide association analyses, with variant-level significance assessed using two-sided tests of association. Genome-wide significance was defined as p < 5 × 10^−8^. Gold-highlighted variants have linkage disequilibrium r^2^ > 0.001 and are within 5 megabases of the lead variants (diamonds). The nearest gene to each lead variant is labeled. The male version of this analysis is not shown here because it would be the same as the bottom half of Extended Data Fig. 2: the male-only meta-analysis is restricted to European males due to low male case counts in non-European ancestries (see Methods).

**Extended Data Fig. 4:**
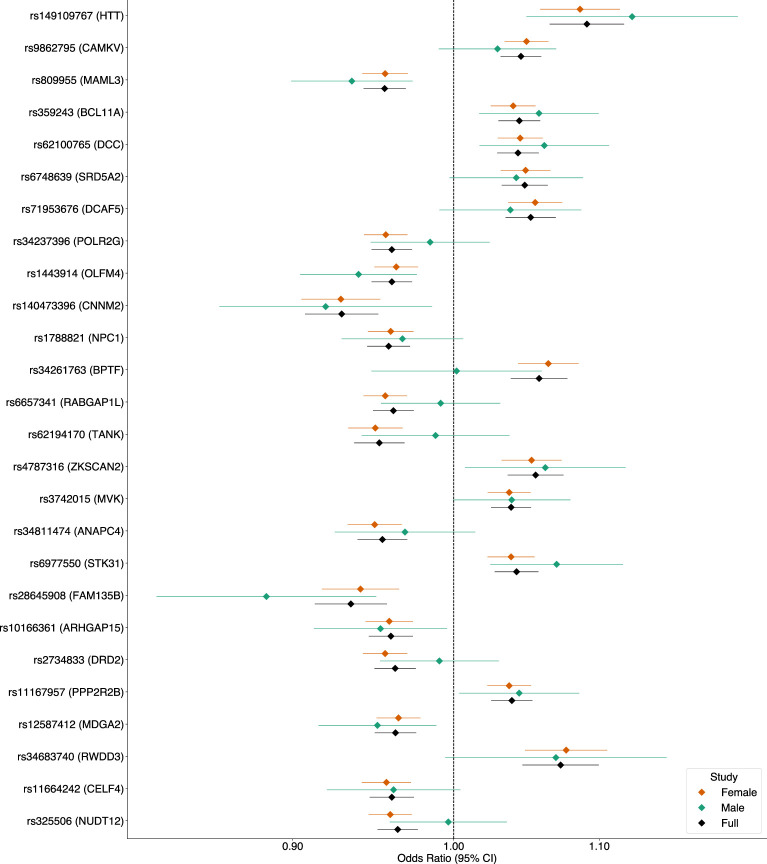
Sex differences in fibromyalgia lead variant associations. Male, female, and full-cohort odds ratios with 95% confidence intervals for the 26 lead variants from the primary meta-analysis.

We performed multiple secondary meta-analyses: leave-one-cohort-out analyses to enable unbiased genetic risk prediction, European-only meta-analyses to reduce bias in LD-based analyses (Extended Data Fig. 1) and sex-stratified meta-analyses (Extended Data Figs. 2 and 3, Supplementary Figs. 2 and 3, and Supplementary Table 2). Consistent with known sex differences in fibromyalgia prevalence^10^, the vast majority of cases (47,895 cases, 87.7%) were female. While the lower number of male cases yielded less statistical power (2 loci in males versus 21 in females), the inter-sex genetic correlation in European-ancestry individuals was 1.03 (*P* = 2.9 × 10^−16^), statistically indistinguishable from perfect correlation, and odds ratios for the 26 lead variants from the primary meta-analysis were similar in males and females (Extended Data Fig. 4). Thus, fibromyalgia’s genetic architecture is largely consistent between sexes.

We prioritized causal genes via manual literature review, complemented by a deCODE genetic annotation pipeline that considers quantitative trait loci and coding variants in strong LD (Supplementary Table 3 and Methods) and the FLAMES machine learning method. We discuss notable prioritizations here, many of which implicate brain function or brain disease.

### The top fibromyalgia association is with a coding variant in the Huntingtin gene

The most significant association in the primary analysis was with rs149109767-A (OR = 1.09, *P* = 2.2 × 10^−12^), an inframe glutamic acid deletion in the Huntingtin (*HTT*) gene. *HTT*, the causal gene for Huntington’s disease (HD), is ubiquitously expressed and involved in diverse cellular processes including signaling, transcriptional regulation, cell division, endocytosis, autophagy and axonal transport, and is required for healthy neurodevelopment^11^. Our fibromyalgia variant is located in exon 58 of the 67-exon *HTT* gene, far from the exon 1 repeat-expansion region that causes HD, but is part of the HD-associated ‘A1’ haplotype found in Europeans^12^. This variant shows the largest effect among the 26 lead variants: carriers have about 9% increased odds of fibromyalgia.

We investigated whether our fibromyalgia variant is associated with HD using a six-European-cohort HD GWAS meta-analysis available at deCODE (*N*_case/control_ = 184/1,198,665). The top HD association (rs71180116-CCAGCAGCAGCAGCAGCAGCAGCAGCAGCAGCAGCAG, OR = 44.3, *P* = 2.62 × 10^−30^) was not in strong LD with our lead fibromyalgia variant (*r*^2^ = 0.0055; Lewontin’s normalized disequilibrium coefficient (*D*) = 0.258) and showed no association with fibromyalgia (OR = 1.027, *P* = 0.41). While our fibromyalgia variant rs149109767-A did associate with HD risk (OR = 2.118, *P* = 0.00354), this association disappeared in the UK Biobank after conditioning on rs71180116 (conditional OR = 1.328, *P* = 0.297), an effect replicated in the smaller Icelandic GWAS.

We found a second association with a possible HD link: rs6657341-A (OR = 0.961, *P* = 7.1 × 10^−9^), an intronic variant in *RABGAP1L*, ~150 kilobases (kb) upstream of its second-nearest gene *GPR52*. While *RABGAP1L* has no clear link to fibromyalgia, GPR52 is a brain-specific orphan G-protein-coupled receptor being investigated as a drug target for HD, as it regulates HTT levels^13,14^.

### Additional associations implicate pathways in pain processing

Several associations implicated pain processing pathways. One involved rs11664242, an intergenic variant ~30 kb upstream of its nearest coding gene *CELF4*. *CELF4* encodes an RNA-binding protein and translational regulator predominantly expressed in excitatory neurons, regulating synaptogenesis during neurodevelopment by modulating translation of synaptic mRNAs^15^. CELF4 reduces pain and sensory sensitivity by negatively regulating nociceptor excitability^16^, and *CELF4* gene therapy is an investigational therapeutic strategy for chronic pain.

Another association was found with rs6977550, an intergenic variant ~30 kb downstream of its nearest coding gene *STK31*. *STK31* encodes a testis-specific protein kinase with unclear relevance to fibromyalgia; however, the variant is ~400 kb upstream of *NPY* (its sixth-nearest gene), an abundant neuropeptide that modulates appetite, circadian rhythm, anxiety and immune responses^17^ and has both pro- and anti-nociceptive effects^18^.

We also identified rs10166361, an intronic variant in *ARHGAP15*, which encodes a Rho GTPase-activating protein that regulates cytoskeletal organization^19^. In mouse models, *ARHGAP15* knockout impairs cortical interneuron excitability (causing subclinical seizures) and hippocampal function (causing memory impairment)^19,20^. The variant is also ~450 kb downstream of *KYNU* (the third-nearest gene), which encodes kynureninase, an enzyme that cleaves kynurenine into anthranilic acid^21^. Kynurenine and its metabolites are involved in pain processing, and the kynurenine pathway is a proposed therapeutic target for pain^22^. Gene-based tests have linked *KYNU* to depressive symptoms, often comorbid with chronic pain^23^.

### Other significant associations point to brain-related mechanisms

One particularly notable brain association was with rs2734833, an intronic variant in *DRD2*, a locus with psychiatric and sleep associations. *DRD2* encodes the dopamine D2 receptor, which influences motivation and cognition^24^, and is the primary target of most antipsychotic drugs. The variant lies ~150 kb downstream of *NCAM1*, the fourth-nearest gene, which encodes a cell surface receptor (CD56) with both nervous and immune roles. In the nervous system, it regulates neural cell adhesion, neuronal migration, neurite outgrowth, axon guidance and synaptic plasticity, and plays a role in memory^25^. In the immune system, CD56 is the primary marker for natural killer cells and distinguishes their two main subtypes. It helps natural killer cells attach to target cells and trigger the release of cytotoxic granules that kill target cells^26^.

The second-most significant association was with rs9862795, an intergenic variant located ~8 kb upstream from *CAMKV*. *CAMKV* encodes a pseudokinase from the Ca^2+^/calmodulin-dependent protein kinase family that is predominantly brain-expressed and critical for dendritic spine maintenance, synaptic transmission and synaptic plasticity^27^. The lead variant is also in strong LD (*r*^2^ = 0.905; Supplementary Table 3) with two variants in a pleiotropic tyrosine kinase receptor gene, *MST1R*, which regulates macrophage activation, inflammation and antiviral defence.

Two other lead variants were rs809955, an intronic variant in *MAML3*, and rs12587412, an intergenic variant ~40 kb downstream of its nearest coding gene *MDGA2*. *MAML3* encodes a transcriptional coactivator in the Notch signaling pathway^28^. Notch signaling is vital for development and cell-fate decisions: in development, inhibition promotes neural differentiation, while in adulthood, activation influences neural stem cell regulation, neuronal survival, migration and synaptic plasticity^29,30^. *MDGA2* is predominantly expressed in the brain and regulates synaptic organization^31^, axonal migration^32^, BDNF/TrkB signaling^33^ and excitatory neurotransmission^31^. *MDGA2* haploinsufficiency is associated with autism spectrum disorder^33^.

Another neurodevelopmental association was with rs62100765, an intronic variant in *DCC*, which was the sole coding gene within 1 megabase (Mb). *DCC* encodes a transmembrane receptor that guides midline-crossing axons during neurodevelopment^34^ and regulates myelin structure and axon domain organization^35^.

We also found an association with rs6748639, an intergenic variant located ~100 kb upstream of the nearest coding gene, *SRD5A2*, and ~350 kb upstream of *SPAST*, the fifth-nearest gene and the one prioritized by FLAMES. SRD5A2 is one of three enzymes that convert testosterone to the more potent androgen dihydrotestosterone, which is critical to normal male sexual development: biallelic loss causes 5α-reductase deficiency, which can result in female-appearing external genitalia^36^. Some studies report an inverse association between testosterone level and pain perception^37^. An alternative causal gene candidate at this locus, *SPAST*, encodes the neuronally expressed protein spastin, which regulates microtubule length and disassembly^38^; haploinsufficiency is the most common cause of hereditary spastic paraplegia^39^.

Several associations implicated genes linked to intellectual disability and developmental disorders. One was with rs359243, an intergenic variant located in a gene-dense region ~200 kb downstream from its nearest coding gene, the transcription factor *BCL11A*. Besides its canonical role in hematopoiesis and hemoglobin regulation, BCL11A has neurodevelopmental roles, and haploinsufficiency causes intellectual disability and autism^40^.

Another developmental disorder association was with rs34261763, an intronic variant in *BPTF*, which encodes a subunit of the developmentally essential NURF chromatin remodeling complex^41^. *BPTF* haploinsufficiency causes a neurodevelopmental disorder with intellectual disability, developmental and speech delay, microcephaly, and facial and limb dysmorphia^41^.

We also found an association with rs11167957, an intronic variant in *PPP2R2B*, a brain-enriched subunit of the serine/threonine phosphatase PP2A (ref. ^42^). CAG repeat expansion in *PPP2R2B* causes spinocerebellar ataxia 12, an autosomal dominant speech and motor disorder^43^, and *PPP2R2B* missense variants are linked to intellectual disability with developmental delay^42^. The variant is also ~550 kb upstream of the orphan brain-specific G protein-coupled receptor gene *GPR151* (its 5th-nearest gene), which mediates neuropathic pain via microglial activation and neuroinflammation^44,45^.

Finally, we found an association with rs1788821, an intronic variant in *NPC1*. *NPC1* encodes a transmembrane protein vital for intracellular cholesterol trafficking, and biallelic loss of NPC1 function causes Niemann–Pick disease type C1, characterized by lysosomal lipid accumulation, neuroinflammation, abnormal dendrite growth, neurofibrillary tangles and neuroaxonal dystrophy^46^. The lead variant is in strong LD with missense variants in *NPC1* and an autophagy-related gene^47^, *RMC1* (*r*^2^ = 0.998 and 0.990, respectively; Supplementary Table 3).

### Phenome-wide associations of lead variants point to substantial pleiotropy

We cross-referenced the 26 lead variants and their high-LD proxies (*r*^2^ > 0.8) with the GWAS Catalog. A total of 20 traits were associated with at least 4 of the 26 lead variants (Fig. 3 (top) and Supplementary Table 4). The most frequent association was with educational attainment (10 variants), followed by body mass index (8), type 2 diabetes (7), pain intensity (6) and insomnia (6). Notable psychiatric associations included depressive symptoms (4 variants), schizophrenia (3), depression (2) and suicide attempt (1). Remaining associations spanned pain, immune and behavioral traits (for example, multisite chronic pain, white blood cell count, neuroticism), and anthropometric and metabolic traits (for example, weight, visceral adipose tissue, metabolic syndrome).

We also performed a phenome-wide association study (PheWAS), associating the 26 lead variants with 330 diseases, leveraging preexisting GWAS meta-analyses of the Million Veteran Program, FinnGen and the UK Biobank (‘MVP–Finngen–UKBB’). After Bonferroni correction across the 330 diseases and 26 variants tested, 20 diseases were associated with 3 or more lead variants (Fig. 3 (middle) and Supplementary Table 5). These diseases largely fell into three categories: pain and pain-associated traits (dorsalgia, spondylosis, joint pain, limb pain, spinal stenosis, cervicalgia), metabolic syndrome and its comorbidities (type 2 diabetes, obesity, sleep apnea, hypertension, coronary heart disease, gout, lipoprotein and lipid disorders, hypercholesterolemia, cholelithiasis) and immune disease (autoimmune hypothyroidism, asthma). The most common association was dorsalgia (back pain, 13 of 26 variants associated); the second-most common was gastroesophageal reflux disease (9 variants).

Applying the same approach to 124 drug prescription GWAS from FinnGen (Fig. 3 (bottom) and Supplementary Table 6), the most frequent associations were with analgesics (14 variants) and anti-inflammatory and anti-rheumatic drugs (12 variants). A total of 11 variants were associated with both categories, reflecting prescription of nonsteroidal anti-inflammatory drugs (included in both categories) for pain. A further 10 drug categories were associated with at least 3 lead variants, including psychoactive drugs (muscle relaxants, psycholeptics, psychoanaleptics), treatments for metabolic syndrome and its comorbidities (diabetes drugs, lipid-modifying agents, renin–angiotensin agents) and immune modulators (anti-asthmatics, anti-histamines).

These results show that the top genetic risk variants for fibromyalgia are highly pleiotropic, broadly influencing risk for many of the disorder’s most common comorbidities and likelihood of prescription of the drugs used to treat them.

### Fibromyalgia heritability is exclusively enriched in neural tissues and cell types

**Fig. 4:**
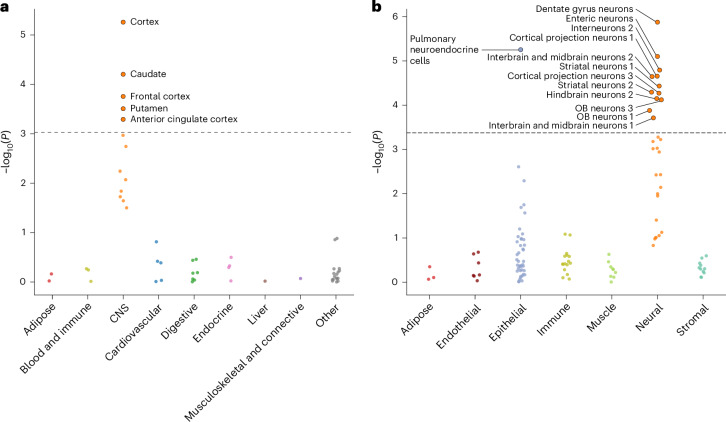
Tissue and cell-type enrichments. **a**,**b**, Fibromyalgia heritability enrichments among variants inside or within 100 kb of genes with enriched expression in tissues in the GTEx project (**a**) and cell types in PanSci, an ~20 million whole-mouse single-cell atlas (**b**). Results are shown as negative log(*P* values) for enrichment, grouped and colored by tissue group (GTEx) or cell lineage (PanSci), with random side-to-side jitter for visibility. Enrichment was tested using LDSC-SEG, with significance assessed using two-sided enrichment *P* values and Bonferroni-significant tissues, and cell types are labeled. Full results are listed in Supplementary Tables 7–9. OB, olfactory bulb.

We used LD score regression applied to specifically expressed genes (LDSC-SEG) to quantify fibromyalgia heritability enrichment near genes with enriched expression in each of 53 tissues in the Genotype-Tissue Expression (GTEx) project, and each of 119 cell types in the ~20 million whole-mouse PanSci single-cell atlas (Fig. 4 and Supplementary Tables 7 and 8). We found strong and exclusive enrichment within the nervous system: all 5 GTEx tissues with Bonferroni-significant enrichment were brain regions (cortex, caudate, frontal cortex, putamen and anterior cingulate cortex), and 12 of 13 enriched cell types were neuronal, with the 13th (pulmonary neuroendocrine cells) being neuron-like.

The strongest cell-type association was with neurons from the dentate gyrus (*P* = 1.3 × 10^−6^), a hippocampal region critical for contextualizing sensory experience, including pain. Significant enrichments were also found in enteric neurons (*P* = 7.9 × 10^−6^) and diverse interneurons, cortical projection neurons and striatal neurons. Even when grouping cell types by lineage with the goal of increasing power, the only significant lineage was neural (*P* = 2.3 × 10^−4^; *P* > 0.05 for all other cell types; Supplementary Table 9).

Collectively, these results implicate a broad network of neurons across the central and peripheral nervous systems, including sensory-processing neurons, in the etiology of fibromyalgia.

### Pervasive genetic correlations with comorbid disorders

**Fig. 5:**
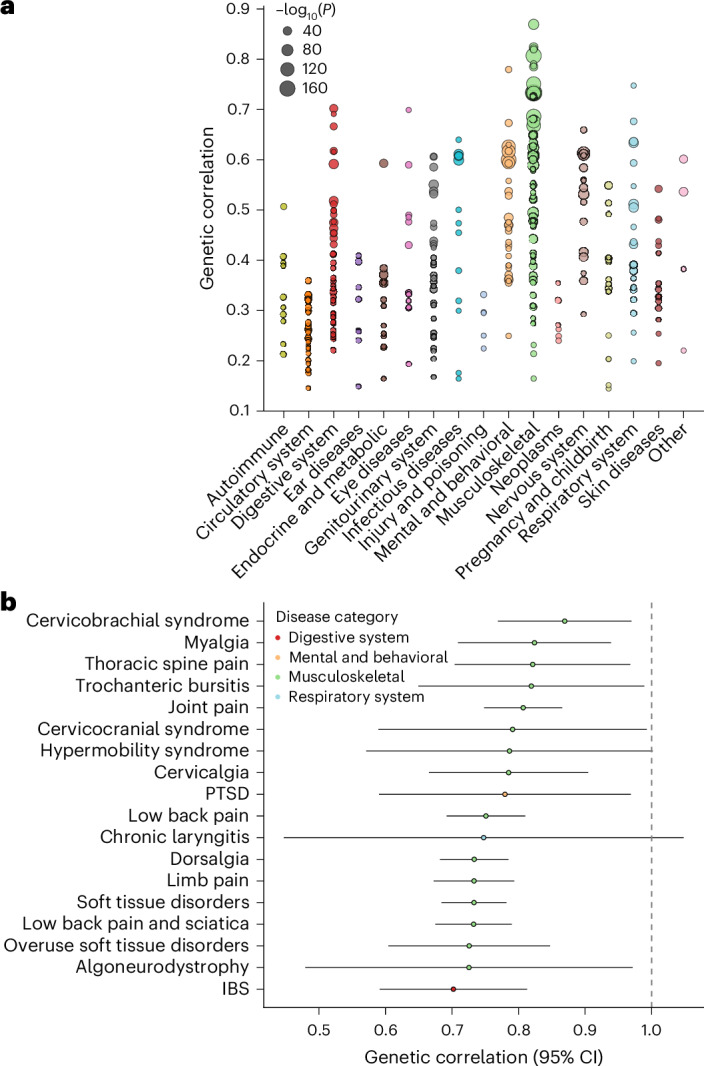
Genetic correlations between fibromyalgia and FinnGen disease endpoints. **a**, Genetic correlations of the 337 disease endpoints with Bonferroni-significant genetic correlations with fibromyalgia, grouped by disease area. Genetic correlations were estimated using LDSC, with significance assessed using two-sided tests of whether the genetic correlation differed from zero and Bonferroni correction for the number of FinnGen endpoints tested. The area of each circle is proportional to the logarithm of its genetic correlation *P* value. **b**, Genetic correlations of the 18 diseases with genetic correlation greater than 0.7; error bars denote 95% CIs around the *r*_g_ point estimate. Full results are listed in Supplementary Table 10. Note that there were no significant negative correlations. PTSD, post-traumatic stress disorder; IBS, irritable bowel syndrome.

We computed genetic correlations between the European-only meta-analysis and each of 855 diseases in FinnGen, yielding 337 Bonferroni-significant genetic correlations (Fig. 5 and Supplementary Table 10).

The strongest genetic overlap was with pain and musculoskeletal disorders. The strongest signals arose from conditions characterized by widespread or syndromic pain, including cervicobrachial syndrome (genetic correlation (*r*_g_) = 0.87, *P* = 9.2 × 10^−65^), myalgia (*r*_g_ = 0.82, *P* = 9.4 × 10^−45^), hypermobility syndrome (*r*_g_ = 0.79, *P* = 8.4 × 10^−13^) and soft tissue disorders (*r*_g_ = 0.73, *P* = 5.0 × 10^−190^). We also observed substantial genetic correlations with a range of common localized pain presentations, from joint pain (*r*_g_ = 0.81, *P* = 1.7 × 10^−160^) and low back pain (*r*_g_ = 0.75, *P* = 1.1 × 10^−137^) to trochanteric bursitis (*r*_g_ = 0.82, *P* = 3.1 × 10^−21^) and migraine (*r*_g_ = 0.61, *P* = 2.2 × 10^−73^).

Fibromyalgia showed substantial genetic correlations with mental and behavioral disorders, with a magnitude for some traits, such as post-traumatic stress disorder (*r*_g_ = 0.78, *P* = 6.6 × 10^−16^), that rivaled those of pain conditions. Strong correlations were also evident for dissociative disorders (*r*_g_ = 0.63, *P* = 5.0 × 10^−6^), somatoform disorder (*r*_g_ = 0.67, *P* = 8.8 × 10^−26^) and depression (*r*_g_ = 0.63, *P* = 4.1 × 10^−118^), alongside more modest correlations with generalized anxiety disorder (*r*_g_ = 0.46, *P* = 3.8 × 10^−20^) and insomnia (*r*_g_ = 0.47, *P* = 2.0 × 10^−43^).

Significant genetic overlap extended to other commonly co-occurring conditions, including disorders of the digestive, genitourinary and respiratory systems. Among digestive disorders, we observed strong signals for irritable bowel syndrome (*r*_g_ = 0.70, *P* = 2.1 × 10^−35^) and functional dyspepsia (*r*_g_ = 0.67, *P* = 9.1 × 10^−24^). We also identified correlation with genitourinary conditions such as polycystic ovarian syndrome (*r*_g_ = 0.59, *P* = 8.3 × 10^−39^) and the respiratory condition chronic laryngitis (*r*_g_ = 0.75, *P* = 1.1 × 10^−6^).

Genetic correlations with autoimmune disorders were comparatively modest. The highest correlations in this category were with psoriatic arthropathies (*r*_g_ = 0.41, *P* = 5.4 × 10^−17^) and Sjögren’s syndrome (*r*_g_ = 0.39, *P* = 1.7 × 10^−6^). Other rheumatological conditions with significant genetic correlations included rheumatoid arthritis (*r*_g_ = 0.33, *P* = 3.1 × 10^−16^), psoriasis (*r*_g_ = 0.29, *P* = 6.0 × 10^−15^) and autoimmune hypothyroidism (*r*_g_ = 0.21, *P* = 4.2 × 10^−17^). Given the genetic correlations observed with asthma (*r*_g_ = 0.51, *P* = 2.5 × 10^−57^) and rheumatoid arthritis (RA) and the long-standing debate on whether fibromyalgia is an (auto)immune disease^4^, we explored genetic correlations with subtypes of these diseases. Asthma can be broadly classified as T2 high or T2 low, defined by high or low levels of T2 molecules such as eosinophils, with half of individuals with asthma falling into each category^48^. While the mechanisms driving T2-high (typical) asthma are increasingly understood, much less is known about T2-low asthma^48^. Using GWAS results from T2-high and T2-low asthma at deCODE^49^, we found much stronger genetic correlation between fibromyalgia and T2-low (*r*_g_ = 0.44, *P* = 3.5 × 10^−24^) relative to T2-high (*r*_g_ = 0.27, *P* = 7.4 × 10^−14^) asthma, consistent with fibromyalgia’s lack of genetic correlation with eosinophil levels (*r*_g_ = 0.04, *P* = 0.1).

Similarly, RA is classified as seronegative or seropositive based on the absence or presence of anticitrullinated protein antibodies and rheumatoid factor, with seronegative RA considered less severe than seropositive RA. We found that fibromyalgia is more genetically correlated with the seronegative form of RA (*r*_g_ = 0.41, *P* = 2.7 × 10^−15^) than with seropositive RA (*r*_g_ = 0.24, *P* = 3.0 × 10^−10^), an association also observed clinically^50^. Thus, fibromyalgia is genetically correlated with the more heterogeneous and less well-defined forms of asthma and RA, further distinguishing fibromyalgia’s genetic architecture from that of well-characterized, peripherally driven inflammatory and autoimmune disease.

Collectively, this landscape of genetic correlations highlights the profound pleiotropy of fibromyalgia genetic risk factors. This apparent paradox—widespread somatic correlations despite a genetic architecture strongly enriched in neuronal cell types—is resolved when considering that many of the abovementioned conditions are themselves rooted in the central nervous system. A partially shared, centrally mediated genetic etiology could drive their frequent co-occurrence with fibromyalgia.

### Genetic risk prediction is modestly effective at patient stratification

**Extended Data Fig. 5:**
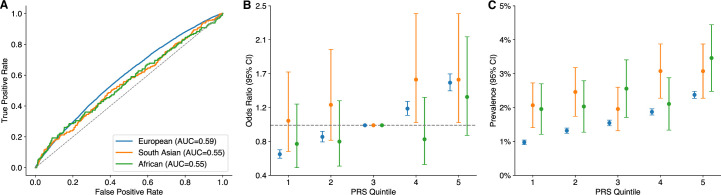
Polygenic risk for fibromyalgia in the UK Biobank. Genetic ancestry-stratified **a**) areas under the receiver-operating curve, **b**) odds ratios with 95% confidence intervals for each quintile of polygenic risk relative to the middle quintile, and **c**) prevalences of fibromyalgia with 95% confidence intervals within each quintile, using polygenic risk scores computed from the multi-ancestry leave-UK Biobank-out meta-analysis.

To enable fibromyalgia genetic risk prediction, we constructed polygenic risk scores (PRSs) for the European and multi-ancestry leave-UK Biobank-out meta-analyses (Data Availability). We evaluated the performance of the multi-ancestry leave-UK Biobank-out PRS within the UK Biobank. The PRS showed modest predictive ability, commensurate with fibromyalgia’s modest observed-scale heritability of 10.4% (95% confidence interval (CI) 9.8% to 11.0%). Performance was highest among European-ancestry UK Biobank participants (area under the receiver-operating curve (AUC) = 0.59), with attenuated South Asian (AUC = 0.55) and African (AUC = 0.55) accuracies (Extended Data Fig. 5a) reflecting the mostly European composition of our samples.

Despite modest AUCs, the PRS effectively stratified individuals by risk, particularly Europeans in which power and predictive accuracy were greatest. Compared with the middle quintile, European-ancestry participants in the highest PRS quintile had a fibromyalgia odds ratio of 1.5 (95% CI 1.4 to 1.7), while those in the lowest quintile had an odds ratio of 0.63 (95% CI 0.57 to 0.68; Extended Data Fig. 5b). This risk stratification translated to marked differences in disease prevalence between the lowest and highest PRS quintiles (Extended Data Fig. 5c): among European-ancestry participants, prevalence increased from approximately 1.0% in the lowest quintile to 2.4% in the highest.

## Discussion

In GWAS meta-analysis of 2.5 million individuals, we identified 26 genetic risk loci for fibromyalgia, providing robust characterization of its genetic architecture. These findings establish a firm biological basis for a long-debated condition.

Fibromyalgia heritability is enriched in the brain and in neuronal cell types, consistent with the central sensitization model of fibromyalgia. The strongest cell-type association was with hippocampal dentate gyrus neurons, involved in episodic memory and contextualizing sensory inputs such as pain; impairment could contribute to fibromyalgia’s cognitive impairment or ‘brain fog’. The second-strongest association was with enteric neurons, which regulate gastrointestinal motility and sensation, supporting involvement of the gut–brain axis in the comorbidity with irritable bowel syndrome. However, genes specifically expressed in closely related cell types often overlap, limiting the confidence with which enrichments can pinpoint precise neuronal populations.

The specific genes implicated at risk loci further refine this model. Multiple candidate causal genes, including *HTT*, *DCC*, *MDGA2* and *CELF4*, have neural roles. Lead variants overlap extensively with brain-related traits in our GWAS Catalog and MVP–Finngen–UKBB PheWAS analyses, including pain traits such as multisite chronic pain (*MAML3*, *DCC*, *OLFM4*, *ANAPC4*) and dorsalgia (*CAMKV/MST1R*, *MAML3*, *BCL11A*, *DCC*, *DCAF5*, *CNNM2*, *RABGAP1L*/*GPR52*, *ZKSCAN2*, *ANAPC4*, *ARHGAP15*, *DRD2*/*NCAM1*, *MDGA2*, *CELF4*), sleep traits such as insomnia (*CAMKV/MST1R*, *DCC*, *OLFM4*, *CNNM2*, *MVK*, *NUDT12*) and canonical psychiatric disorders such as depression (*DCC*, *NUDT12)* and schizophrenia (*CNNM2*, *STK31*/*NPY*, *PPP2R2B*/*GPR151*). In addition, risk loci overlapped with long COVID^51^ (*BPTF*) and myalgic encephalomyelitis/chronic fatigue syndrome^52^ (*OLFM4*, *RABGAP1L/GPR52*), two poorly characterized disorders with a hypothesized neural basis, albeit with different lead variants. That said, some brain-related traits have much stronger genetic correlation with fibromyalgia than others: for instance, pain traits and post-traumatic stress disorder have higher correlations than depression and schizophrenia, which in turn have higher genetic correlation than insomnia.

Our strongest association (~9% increased risk of fibromyalgia) was with a common coding variant in *HTT*, the causal gene for HD, although this variant is distinct from the rare repeat expansion that causes HD. The variant results in the deletion of a single glutamic acid residue in the HTT protein. We also observe an association near *GPR52*, a regulator of *HTT*. *HTT* could contribute to fibromyalgia etiology via both central and peripheral nervous system mechanisms. Centrally, *HTT* is widely expressed, especially in neurons, and HD neuronal dysfunction is concentrated in pain-related brain regions such as the striatum^53^, where we observe enriched fibromyalgia heritability. Peripherally, *HTT* may affect nociception and spinal nociceptive relay: mouse *Htt* is expressed in dorsal root ganglion (DRG) neurons, where it aids peripheral axon regeneration after sciatic nerve injury^54^, as well as in the superficial dorsal horn laminae of the CNS that receive nociceptive inputs from the DRG. While HTT has limited direct evidence of a functional role in peripheral nociception, its interactor Huntingtin-associated protein 1 (HAP1) has stronger evidence. Mouse *Hap1* is expressed in nociceptive DRG subpopulations and spinal dorsal horn neurons^55^ and upregulated in these regions in chronic pain conditions^56^. *Hap1* heterozygous knockout mice have reduced nociceptive neuron excitability and attenuated spinal glial activation after nerve injury^56^.

The genetic underpinnings of fibromyalgia are shared with a remarkably wide variety of other disease types. Genetic correlations implicate digestive, genitourinary and respiratory systems. One explanation is that fibromyalgia genetics captures a transdiagnostic central nervous system vulnerability that predisposes individuals to sensory and affective dysregulation. This vulnerability might manifest clinically as fibromyalgia, irritable bowel syndrome, post-traumatic stress disorder or a constellation of other disorders, depending on other genetic and environmental factors. On the other hand, this pleiotropy could merely reflect genetic influence on comorbid conditions that secondarily increase fibromyalgia risk, such as obesity, which also has a strongly neural genetic architecture.

Our results inform the controversy over whether fibromyalgia is an autoimmune disorder^4^. We did not find a significant GWAS signal in the major histocompatibility complex (MHC) region, nor heritability enrichment in either peripheral immune cell types or glia, but did observe modest genetic correlations between fibromyalgia and autoimmune disorders including Sjögren’s syndrome and RA. These associations should be interpreted with caution in light of the potential for diagnostic misclassification of fibromyalgia as Sjögren’s syndrome or RA^57,58^. Notably, recent work using a broader phenotype that included both ICD-10 code-based and self-reported fibromyalgia reported autoimmune-related genetic signal, although differences in case definition may partly explain the apparent discrepancy with our findings^59^. Further supporting divergence between fibromyalgia and classical autoimmune disorders, genetic correlation was stronger with the more atypical seronegative subtype of RA than the seropositive subtype. Overall, our results suggest that fibromyalgia is not primarily an autoimmune disorder, although it may nonetheless have a peripheral immune and/or neuroimmune component. Power to detect this may have been limited by the predominantly European composition of our sample and by healthy participant bias in biobank cohorts.

Despite several-fold-higher prevalence in females, we found no sex difference in the genetic architecture of fibromyalgia. The observed difference in prevalence is therefore unlikely to be driven by sex-specific genetic risk variants, and may instead reflect nongenetic biological factors, environmental exposures or diagnostic bias^60^.

This study identifies two potential therapeutic targets. The linked associations of *HTT* and its regulator *GPR52* highlight a clear repurposing opportunity, as GPR52 is already an investigational drug target for HD. Our association with *CELF4* supports developing *CELF4*-based gene therapies—already under investigation for chronic pain—for fibromyalgia in particular. These loci represent genetically supported molecular targets for fibromyalgia.

This study has important limitations. Our cohort was mostly European, limiting the generalizability of findings and transferability of our PRSs to non-European ancestries. Our reliance on ICD codes may result in the inclusion of individuals not meeting full syndromic criteria for fibromyalgia, or the exclusion of undiagnosed cases. Incorporating phenotype risk scores^61^ into fibromyalgia phenotyping might mitigate diagnostic heterogeneity between cohorts, or exclude controls with conditions such as depression or back pain.

Our study maps the genetic architecture of fibromyalgia, identifying 26 risk loci and providing robust genetic validation of the notion that fibromyalgia is primarily a central nervous system disorder. Identifying specific risk loci provides the field with concrete molecular starting points, enabling hypothesis-driven studies of pathophysiology and shared etiology with comorbid conditions.

## Methods

### Ethics approval

All of Us (All of Us Institutional Review Board), UK Biobank (North West Multi-centre Research Ethics Committee/North West–Haydock Research Ethics Committee), FinnGen (Coordinating Ethics Committee of the Hospital District of Helsinki and Uusimaa), Estonian Biobank (Estonian Committee on Bioethics and Human Research, Estonian Ministry of Social Affairs), Genes & Health (London–South East Research Ethics Committee/NRES Committee London–South East), Mass General Brigham Biobank (Mass General Brigham Institutional Review Board/Mass General Brigham Human Research Committee), Michigan Genomics Initiative (University of Michigan Medical School Institutional Review Board), deCODE Genetics/Amgen Iceland (National Bioethics Committee of Iceland and Icelandic Data Protection Authority), Copenhagen Hospital Biobank (CHB; Danish National Committee on Health Research Ethics and Capital Region Data Protection Agency), Danish Blood Donor Study (DBDS; Danish National Committee on Health Research Ethics and Danish Data Protection Agency), Intermountain Health (Intermountain Healthcare Institutional Review Board), and Nashville Biosciences/BioVU (Vanderbilt University Medical Center Institutional Review Board) gave ethical approval for this work. Participants were not compensated for this study.

### Phenotype definition

Cases were individuals with at least one inpatient or primary care record containing ICD-10 code M79.7 (fibromyalgia; Supplementary Table 11). No exclusion criteria were applied: controls were all genotyped participants without code M79.7. Phenotype ascertainment was performed independently within each cohort before genome-wide association analysis.

### Genotyping, imputation and quality control

We included autosomal and chromosome X data for all cohorts.

#### All of Us

We obtained whole-genome sequencing (WGS), hg38 data from All of Us’s Curated Data Repository version 8 release, which involved variant calling, initial quality control and genetic ancestry assignment. We used the allele count/allele frequency (ACAF) threshold callset, which contains variants with a minor allele count >100 or minor allele frequency >1% in any genetic ancestry group, as the basis for a second round of ancestry-specific quality control with version 2.0.0 of the plink genetic analysis toolkit^62^, in which variants with minor allele frequency (MAF) < 0.1%, missingness >10% or Hardy–Weinberg equilibrium (HWE) *P* < 1 × 10^−15^ (with mid-*P* correction) were removed. Participants flagged by All of Us in the first round of quality control, those with sex-chromosome aneuploidy or sex at birth not equal to male or female, were excluded. We performed association analysis on each of the 3 largest genetic ancestry groups—EUR, AFR and AMR—with version 3.3 of the regenie GWAS toolkit^63^ applying Firth approximation for variants with association value < 0.01, and using as covariates age, sex, age^2^, age × sex, age^2^ × sex and the first 10 genotype principal components (PCs). For step 1 of regenie, we used an LD-pruned subset of the full genotypes, calculated with plink version 2.0.0 via the option ‘–indep-pairwise 500 kb 1 0.2’.

We gratefully acknowledge All of Us participants for their contributions, without whom this research would not have been possible. We also thank the National Institutes of Health’s All of Us Research Program for making available the participant data examined in this study.

#### UK Biobank

We obtained imputed genotypes from the UK Biobank’s Data-Field 22828. The UK Biobank imputed genotypes using a combination of two GRCh37 reference panels, the Haplotype Reference Consortium and a combined UK10K and 1000 Genomes Phase 3 panel, using the Haplotype Reference Consortium imputation if a variant was present in both panels^64^. We excluded samples with sex-chromosome aneuploidy (Data-Field 22019), that were outliers for heterozygosity or missingness rate (Data-Field 22027), or that had discordant genetic sex (Data-Field 22001) versus self-reported sex (Data-Field 31). We excluded variants with MAF < 0.1%, imputation INFO score <0.8, missingness >10% or HWE *P* < 1 × 10^−15^ (with mid-*P* correction) with plink version 2.0.0. We performed associations on the 3 largest genetic ancestry groups as defined by Pan-UKBB (Return 2442)—EUR, Central and South Asian (CSA) and AFR—with version 3.4.1 of regenie, applying Firth approximation for variants with association *P* value < 0.01 and using as covariates age, sex, age^2^, age × sex, age^2^ × sex and the first 10 genotype PCs. For step 1 of regenie, we used an LD-pruned subset of the full genotypes, calculated with the ‘–indep-pairwise 500 kb 1 0.2’ option from plink version 2.0.0.

We thank all participants of the UK Biobank. This research has been conducted using the UK Biobank Resource under application number 116200.

#### FinnGen

Genotyping in the FinnGen cohort was performed by using Illumina and Affymetrix arrays (Thermo Fisher Scientific) and lifted over to GRCh38/hg38. Individuals with high genotype absence (>5%), inexplicit sex or excess heterozygosity (±4 standard deviations) were excluded from the data. In addition, variants that had high absence (>2%), low minor allele count (<3) or low HWE (*P* < 1 × 10^−6^) were removed. More detailed explanations of the genotyping, quality control and genotype imputation are provided elsewhere^65^. All individuals in the cohort were Finns and matched against the SiSu v4 reference panel.

For the FinnGen cohort (Data Freeze 12), GWAS was conducted using the regenie pipeline (https://github.com/FINNGEN/regenie-pipelines). Analysis was adjusted for age at death or end of follow-up, sex, genotyping batches and the first ten genetic PCs. Firth approximation was applied for variants with association *P* value < 0.01.

Study subjects in FinnGen provided informed consent for biobank research, based on the Finnish Biobank Act. Alternatively, separate research cohorts, collected before the Finnish Biobank Act came into effect (in September 2013) and start of FinnGen (August 2017), were collected based on study-specific consents and later transferred to the Finnish biobanks after approval by the Finnish Medicines Agency and the National Supervisory Authority for Welfare and Health. Recruitment protocols followed the biobank protocols approved by the Finnish Medicines Agency. The Coordinating Ethics Committee of the Hospital District of Helsinki and Uusimaa (HUS) statement number for the FinnGen study is Nr HUS/990/2017.

The FinnGen study is approved by the Finnish Institute for Health and Welfare (permit numbers THL/2031/6.02.00/2017, THL/1101/5.05.00/2017, THL/341/6.02.00/2018, THL/2222/6.02.00/2018, THL/283/6.02.00/2019, THL/1721/5.05.00/2019 and THL/1524/5.05.00/2020), Digital and Population Data Service Agency (permit numbers VRK43431/2017-3, VRK/6909/2018-3, VRK/4415/2019-3), the Social Insurance Institution (permit numbers KELA 58/522/2017, KELA 131/522/2018, KELA 70/522/2019, KELA 98/522/2019, KELA 134/522/2019, KELA 138/522/2019, KELA 2/522/2020 and KELA 16/522/2020), Findata (permit numbers THL/2364/14.02/2020, THL/4055/14.06.00/2020, THL/3433/14.06.00/2020, THL/4432/14.06/2020, THL/5189/14.06/2020, THL/5894/14.06.00/2020, THL/6619/14.06.00/2020, THL/209/14.06.00/2021, THL/688/14.06.00/2021, THL/1284/14.06.00/2021, THL/1965/14.06.00/2021, THL/5546/14.02.00/2020, THL/2658/14.06.00/2021 and THL/4235/14.06.00/2021), Statistics Finland (permit numbers TK-53-1041-17 and TK/143/07.03.00/2020 (earlier TK-53-90-20) TK/1735/07.03.00/2021, TK/3112/07.03.00/2021) and Finnish Registry for Kidney Diseases permission and extract from the meeting minutes on 4 July 2019.

The Biobank Access Decisions for FinnGen samples and data used in FinnGen Data Freeze 12 include THL Biobank BB2017_55, BB2017_111, BB2018_19, BB_2018_34, BB_2018_67, BB2018_71, BB2019_7, BB2019_8, BB2019_26, BB2020_1 and BB2021_65; Finnish Red Cross Blood Service Biobank 7.12.2017; Helsinki Biobank HUS/359/2017, HUS/248/2020, HUS/430/2021 §28 and §29, HUS/150/2022 §12, §13, §14, §15, §16, §17, §18, §23, §58 and §59, and HUS/128/2023 §18; Auria Biobank AB17-5154 and amendment 1 (17 August 2020) and amendments BB_2021-0140, BB_2021-0156 (26 August 2021, 2 Feb 2022), BB_2021-0169, BB_2021-0179, BB_2021-0161, AB20-5926 and amendment 1 (23 April 2020) and its modifications (22 September 2021), BB_2022-0262, BB_2022-0256, Biobank Borealis of Northern Finland_2017_1013, 2021_5010, 2021_5010 Amendment, 2021_5018, 2021_5018 Amendment, 2021_5015, 2021_5015 Amendment, 2021_5015 Amendment_2, 2021_5023, 2021_5023 Amendment, 2021_5023 Amendment_2, 2021_5017, 2021_5017 Amendment, 2022_6001, 2022_6001 Amendment, 2022_6006 Amendment, 2022_6006 Amendment, 2022_6006 Amendment_2, BB22-0067, 2022_0262, 2022_0262 Amendment, Biobank of Eastern Finland 1186/2018 and amendment 22§/2020, 53§/2021, 13§/2022, 14§/2022, 15§/2022, 27§/2022, 28§/2022, 29§/2022, 33§/2022, 35§/2022, 36§/2022, 37§/2022, 39§/2022, 7§/2023, 32§/2023, 33§/2023, 34§/2023, 35§/2023, 36§/2023, 37§/2023, 38§/2023, 39§/2023, 40§/2023 and 41§/2023; Finnish Clinical Biobank Tampere MH0004 and amendments (21.02.2020 and 06.10.2020), BB2021-0140 8§/2021, 9§/2021, §9/2022, §10/2022, §12/2022, 13§/2022, §20/2022, §21/2022, §22/2022, §23/2022, 28§/2022, 29§/2022, 30§/2022, 31§/2022, 32§/2022, 38§/2022, 40§/2022, 42§/2022 and 1§/2023; Central Finland Biobank 1-2017, BB_2021-0161, BB_2021-0169, BB_2021-0179, BB_2021-0170, BB_2022-0256 and BB_2022-0262, BB22-0067; decision allowing to continue data processing until 31 August 2024 for projects BB_2021-0179, BB22-0067,BB_2022-0262, BB_2021-0170, BB_2021-0164, BB_2021-0161 and BB_2021-0169; Terveystalo Biobank STB 2018001 and amendment 25 August 2020; Finnish Hematological Registry and Clinical Biobank decision 18 June 2021; and Arctic biobank P0844: ARC_2021_1001.

#### Estonian Biobank

All the Estonian Biobank participants have been genotyped at the Core Genotyping Lab of the Institute of Genomics, University of Tartu, using Illumina Global Screening Array (versions 1, 2 or 3). Samples were genotyped and PLINK format files were created using Illumina GenomeStudio v2.0.4. Individuals were excluded from the analysis if their call rate was less than 95%, if they were outliers of the absolute value of heterozygosity (>3 standard deviations from the mean) or if sex defined based on heterozygosity of the X chromosome did not match sex in phenotype data. Before imputation, variants were filtered by call rate <95%, HWE *P* < 1 × 10^−4^ (autosomal variants only) and minor allele frequency <1%. Genotyped variant positions were in build 37 and were lifted over to build 38 using Picard. Phasing was performed using the Beagle v5.4 software. Imputation was performed with Beagle v5.4 software (22 July 2022 release) and default settings. The dataset was split into batches of 5,000. A population-specific reference panel consisting of 2,695 WGS samples was used for imputation, and standard Beagle hg38 recombination maps were used. On the basis of the PC analysis, samples that were not of European ancestry were removed. Duplicate and monozygous twin detection was performed with KING 2.2.7, and one sample was removed from the pair of duplicates. Analyses were restricted to individuals of European ancestry.

Association analysis in the Estonian Biobank was carried out for all variants with an INFO score >0.4 using the additive model as implemented in regenie v3.0.3 with standard binary trait settings. Logistic regression was carried out with adjustment for current age, age^2^, sex and the first 10 genetic PCs as covariates, analyzing only variants with a minimum minor allele count of 2.

The activities of the Estonian Biobank are regulated by the Human Genes Research Act, which was adopted in 2000 specifically for the operations of the Estonian Biobank. Individual-level data analysis in the Estonian Biobank was carried out under ethical approval 1.1-12/624 from the Estonian Committee on Bioethics and Human Research (Estonian Ministry of Social Affairs), using data according to release application 3-10/GI/31689 from the Estonian Biobank.

We want to acknowledge the participants of the Estonian Biobank for their contributions. The Estonian Genome Center analyses were partially carried out in the High Performance Computing Center, University of Tartu. The Estonian Biobank Research Team was responsible for data collection, genotyping, quality control and imputation and consisted of A. Metspalu (andres.metspalu@ut.ee), M. Metspalu (mait.metspalu@ut.ee), L. Milani (lili.milani@ut.ee), R. Mägi (reedik.magi@ut.ee), M. Nelis (mari.nelis@ut.ee), T. Esko (tonu.esko@ut.ee) and G. Hudjashov (georgi.hudjashov@ut.ee).

#### Genes & Health

Genotyping was performed on Illumina Infinium Global Screening Array v3 with additional multi-disease variants. Variants with call rates less than 0.99 and/or MAF < 1% were excluded. We excluded individuals unlikely to have genetically inferred Pakistani or Bangladeshi ancestry. Imputation was performed using the TOPMed-r2 panel. We excluded single nucleotide polymorphisms (SNPs) with low imputation scores (INFO < 0.3). In the Genes & Health cohort, sex was defined on the basis of XX (female) and XY (male) chromosomal presence in genotype data.

Diagnoses were curated from routine UK NHS EHR data from primary care (Systematized Nomenclature of Medicine coded) and secondary care (ICD-10 coded) sources. Data were combined without mapping between coding formats. For each clinical code, the earliest ever measure recorded in a participant’s medical records was used, excluding erroneous code dates preceding the participant’s recorded date of birth. Association analysis was run in regenie, applying Firth approximation for variants with association *P* value < 0.01 and using as covariates age, sex, age^2^ and the first 20 genotype PCs.

Genes & Health is and has recently been core funded by Wellcome (WT102627, WT210561), the Medical Research Council (UK) (M009017, MR/X009777/1, MR/X009920/1), Higher Education Funding Council for England Catalyst, Barts Charity (845/1796), Health Data Research UK (for London substantive site) and research delivery support from the NHS National Institute for Health Research Clinical Research Network (North Thames). We acknowledge the support of the National Institute for Health and Care Research Barts Biomedical Research Centre (NIHR203330); a delivery partnership of Barts Health NHS Trust, Queen Mary University of London, St George’s University Hospitals NHS Foundation Trust and St George’s University of London.

Genes & Health is and has recently been funded by Alnylam Pharmaceuticals, Genomics and a Life Sciences Industry Consortium of AstraZeneca, Bristol-Myers Squibb, GlaxoSmithKline Research and Development, Maze Therapeutics, Merck Sharp & Dohme, Novo Nordisk A/S, Pfizer and Takeda Development Centre Americas.

We thank Social Action for Health, Centre of The Cell, members of our Community Advisory Group and staff who have recruited and collected data from volunteers. We thank the NIHR National Biosample Centre (UK Biocentre), the Social Genetic & Developmental Psychiatry Centre (King’s College London), Wellcome Sanger Institute and Broad Institute for sample processing, genotyping, sequencing and variant annotation. This study uses data provided by patients and collected by the NHS as part of their care and support. This research used Queen Mary University of London’s Apocrita HPC facility, supported by QMUL Research-IT, 10.5281/zenodo.438045.

We thank Barts Health NHS Trust, NHS Clinical Commissioning Groups (City and Hackney, Waltham Forest, Tower Hamlets, Newham, Redbridge, Havering, Barking and Dagenham), East London NHS Foundation Trust, Bradford Teaching Hospitals NHS Foundation Trust, Public Health England (especially D. Wyllie), Discovery Data Service and Endeavour Health Charitable Trust (especially D. Stables), Voror Health Technologies (especially S. Don), NHS England (for what was NHS Digital) for GDPR-compliant data sharing backed by individual written informed consent.

Most of all, we thank all of the volunteers participating in Genes & Health.

A favorable ethical opinion for the main Genes & Health research study was granted by NRES Committee London - South East (reference 14/LO/1240) on 16 September 2014. Queen Mary University of London is the sponsor and data controller.

#### Mass General Brigham Biobank

The Mass General Brigham Biobank genotyped 53,297 participants on the Illumina Global Screening Array (GSA) and 11,864 on Illumina Multi-Ethnic Global Array (MEG). The GSA arrays captured approximately 652,000 SNPs and short insertions–deletions (indels), while the MEG arrays captured approximately 1.38 M SNPs and short indels. These genotypes were filtered for high missingness (>2%) and variants out of HWE (*P* < 1 × 10^−12^), as well as variants with an allele frequency (AF) discordant (*P* < 1 × 10^−150^) from a synthesized AF calculated from gnomAD subpopulation frequencies and a genome-wide GnomAD model fit of the entire cohort. This resulted in approximately 620,000 variants for the GSA and 1.15 M for MEG. The two sets of genotypes were then separately phased and imputed on the TOPMed imputation server (Minimac4 algorithm) using the TOPMed-r2 reference panel. The resultant imputation sets were both filtered at an *r*^2^ > 0.4 and an MAF > 0.001, and then the two sets were merged and intersected resulting in approximately 19.5 M hg38 autosomal variants. The sample set for analysis was then restricted to just those classified as EUR according to a random-forest classifier trained with the Human Genome Diversity Project^66^ as the reference panel, with the minimum probability for assignment to an ancestral group of 0.5, in 19 of 20 iterations of the model. To correct for population stratification, PCs were computed in genetically European participants. Association analysis for the full cohort (*N* = 51,053) was performed with variants using regenie (v3.2.8)^63^ with adjustment for age, sex, genotype-chip, tranche and the first ten genetic PCs. The summary statistics were filtered with a minimum minor allele count of 50. These summary statistics were then lifted over to human genome version 19 (hg19).

In the MGB Biobank, sex-specific association analyses were performed in the hg38 build and carried out using regenie v3.2.8 with covariates of age, tranche, genotype-chip and the first 10 PCs of ancestry calculated by performing the within-EUR principal component analysis (PCA). The summary statistics were filtered with a minimum minor allele count of 25.

#### Michigan Genomics Initiative

We included autosomal and chromosome X data from the Michigan Genomics Initiative (MGI), a health system-based biobank of patients recruited primarily during surgical encounters at Michigan Medicine. As of Freeze 6, MGI comprised 80,529 participants with linked genotype and electronic health record data. Detailed descriptions of the MGI cohort, recruitment protocols and overall design are available in a previous study^67^.

DNA samples were genotyped at the University of Michigan Advanced Genomics Core on customized versions of the Illumina Infinium CoreExome-24 (v1.0, v1.1, v1.3; ~570,000 markers) or Illumina Infinium GSA (v1.3; ~682,000 markers). Array content included standard backbones with additional custom content to capture GWAS candidate variants, predicted loss-of-function alleles, ancestry-informative markers and pharmacogenomic variants. Genotype calling was performed in GenomeStudio, supplemented by zCall for recovery of rare variants.

Sample-level quality control (QC) excluded individuals for consent withdrawal, genotype-inferred sex mismatch, sex-chromosome aneuploidy, call rate <99%, contamination >2.5%, unresolved technical duplicates or batch-level DNA extraction issues. Relatedness was estimated with KING v2.1.3, and contamination with VICES. Variant-level QC removed probes that did not uniquely map to hg38, sites with call rate <98%, HWE *P* < 1 × 10^−4^ in unrelated European-ancestry samples or poor clustering metrics (GenTrain <0.15, cluster separation <0.3). Additional harmonization excluded variants with large allele frequency deviations compared with 1000 Genomes reference populations.

Phasing was performed with Eagle v2.4 using the TOPMed reference panel, followed by imputation against TOPMed haplotypes via the Michigan Imputation Server. Post-imputation, variants with *r*^2^ < 0.3 or MAF < 0.01% were removed, yielding ~52 million high-quality variants. PCs were calculated using FlashPCA2 after pruning rare and correlated variants.

For GWAS, we analyzed participants of genetically inferred European ancestry as defined by PCA projection and ADMIXTURE (*K* = 7 reference populations from the Human Genome Diversity Project). Association testing was performed with SAIGE v0.35, a generalized mixed-model framework that accounts for relatedness and case–control imbalance. Covariates included age, sex, genotyping array and the first ten genetic PCs. Variants with MAF ≥ 0.01% and imputation *r*^2^ ≥ 0.3 were included in association testing.

We acknowledge the Michigan Genomics Initiative participants, AI & Digital Health Innovation at the University of Michigan, the University of Michigan Medical School Central Biorepository and the University of Michigan Advanced Genomics Core for providing data and specimen storage, management, processing and distribution services, and the Center for Statistical Genetics in the Department of Biostatistics at the School of Public Health for genotype data curation, imputation and management in support of the research reported in this Article.

#### deCODE Genetics/Amgen Iceland

At the time of analysis, 63,118 samples from Icelandic participants were whole-genome sequenced at deCODE using Illumina standard TruSeq methods to a mean depth of 38× (ref. ^68^). Only samples with a genome-wide average coverage of 20× and higher were included. Genotypes of SNPs and indels were identified and called jointly by Graphtyper (v.2.7.1)^69^. In all, 173,025 samples from Icelandic participants had been chip-genotyped using various Illumina SNP arrays^68^. The chip-typed individuals were long-range phased^70^, and the variants identified in the whole-genome sequences of Icelanders imputed into the chip-typed individuals. Using extensive and encrypted Icelandic genealogy data, familial imputation of genotypes in first- and second-degree relatives was used to increase sample size^68,70^. The final dataset used included 19,657,761 variants with imputation information over 0.8 and MAF over 0.1%.

The Icelandic samples and diagnostic data were obtained from Icelandic medical record data repositories and analyzed under approval from the National Bioethics Committee (17-035-V11-S2, previously 12-162) following review by the Icelandic Data Protection Authority. Data were anonymized and encrypted by a third-party system, approved and monitored by the Icelandic Data Protection Authority.

#### CHB and the DBDS study

We obtained genotypes from the CHB^71^ study on pain and degenerative musculoskeletal diseases (CHB-PDS; approval: NVK-1803812, P-2019-51) and the DBDS (approval: NVK-1700407, P-2019-99)^72^. All samples were genotyped on Illumina’s Infinium Global Screening Array (versions 1.0 and 3.0) and imputed to build hg38. Genotyping and imputation were performed by deCODE Genetics. Before imputation, duplicate samples and those with genotype call rates <98% were removed. Phasing was carried out with SHAPEIT4 (ref. ^73^), and imputation was performed using deCODE’s in-house workflow, with a joint Graphtyper-based reference panel comprising ~50,000 individuals, including ~10,800 Danes, including danmac5.dk (ref. ^74^). Initial quality control of genotypes included removal of samples with sex discrepancies, missingness >5% and variants with missingness >10%, or HWE *P* < 1 × 10^−5^. For genome-wide association analyses, we removed variants with MAF < 0.1%, imputation INFO < 0.8, missingness >10% or HWE *P* < 1 × 10^−15^ (with mid-*P* correction), using plink version 2.0.0.

#### Intermountain Health

Under research collaboration between Intermountain Health and deCODE Genetics/Amgen, samples were obtained from consenting participants of European descent in two ongoing studies: the Intermountain Inspire Registry and the HerediGene Population Study^75^. The Intermountain Healthcare Institutional Review Board approved both studies, and all participants provided written informed consent before enrollment. Samples were genotyped at deCODE Genetics/Amgen using Illumina Global Screening Array chips. In all, 138,006 individuals of European origin were chip typed. Intermountain and Danish imputation was based on a multi-ethnic reference panel of 50,179 whole-genome-sequenced individuals of mostly European descent, including 23,288 individuals from Intermountain and 11,722 individuals from Denmark.

Samples were filtered on 98% variant yield and duplicates removed. The WGS protocol was the same as described above for the Icelandic and Danish data. Sequence variants were imputed into 138,006 chip-typed individuals. Over 245 million high-quality sequence variants and indels, sequenced to a mean depth of 20×, were identified using Graphtyper (v.2.7.1)^69^. Quality-controlled chip genotype data were phased using SHAPEIT4 (ref. ^73^). A phased haplotype reference panel was prepared from the sequence variants using the long-range phased chip-genotyped samples^68^. In all, 21,316,504 variants (imputation info > 0.8 and MAF > 0.1%) were tested.

#### Nashville Biosciences

Nashville Biosciences (NashBio) is a data and analytics provider owned by Vanderbilt University Medical Center (VUMC). NashBio uses the biobank collection BioVU of VUMC that includes a collection of de-identified DNA samples linked to de-identified data from the electronic health records of VUMC referred to as the Synthetic Derivative (SD) database. All patients of VUMC consented to their residual samples from routine clinical testing and data being contributed to BioVU.

The NashBio dataset included in this study is a subset of BioVU, consisting of, in total, 80,965 whole-genome-sequenced individuals of genetically defined European descent and 31,025 individuals of genetically defined African descent. All samples were whole genome sequenced at deCODE Genetics/Amgen using Illumina NovaSeq in accordance with deCODE/Amgen protocols (deCODE Genetics/Amgen Iceland). Using the same quality criteria as for other analyzed deCODE/Amgen datasets, in total, 22,316,589 variants were tested in the European cohort and 33,108,534 in the African cohort.

BioVU extracts and banks germline DNA samples that are de-identified and only linked to the SD through a randomly assigned unique identifier, not back to the patient or their underlying medical record. The use of BioVU is classified as non-human subject research by the Institutional Review Board (IRB) of VUMC, and NashBio is not required to seek study-specific consent for use of these datasets. The overall biobanking program is reviewed annually by the IRB to maintain this determination and make decisions about patient protections, privacy and ethical issues. Each individual study seeking to use the SD database and BioVU biobank is filed with the IRB of VUMC to validate its non-human subject classification and appropriate use of data.

#### Genetic ancestry analysis in deCODE datasets

For the non-Icelandic datasets analyzed at deCODE Genetics/Amgen (Copenhagen Hospital Biobank and the DBDS Intermountain Health and Nashville Biosciences), genetic ancestry analysis was performed to identify a subset of individuals with similar ancestry. For the Danish samples, we used ADMIXTURE (v1.23)^76^ run in supervised mode using the 1000 Genomes populations CEU (Utah residents with northern and western European ancestry), CHB (Han Chinese in Beijing, China), ITU (Indian Telugu in the UK), PEL (Peruvian in Lima, Peru) and YRI (Yoruba in Ibadan, Nigeria) as training samples. These training samples had themselves been filtered for ancestry outliers using PCA and unsupervised ADMIXTURE. Samples assigned <0.93 CEU were excluded, resulting in 358,483 samples included in the analysis. For the Intermountain samples, we used the same methods as for the Danish samples to identify a subset of 108,149 individuals of European descent that were included in the analysis.

To identify ancestry-homogeneous subsets in the Nashville Biosciences cohort, sample ancestry was estimated using similar methods as in the Danish cohort. Samples with admixed African and European ancestry (defined as YRI + CEU > 0.9, YRI > 0.3 and CEU > 0.02) or predominantly African ancestry (YRI > 0.9) were placed in the AFR subcohort. Genotypes were prepared for PCA with PLINK v1.9 (ref. ^62^) using–maf 0.01–thin-count 1000000–indep-pairwise 60000 6000 0.3 to minimize effects of local LD and very recent population structure. Pairwise relatedness was calculated using KING v2.3.0–ibdseg–degree 3 (ref. ^77^) to identify relatives of third degree or closer, with one sample from each pair of relatives removed before PCA and projected onto the resulting PCs using a deCODE in-house script that adjusts for shrinkage. PCA was performed with PCAone v0.3.4 (ref. ^78^) using default parameters.

#### Association testing in deCODE datasets

The four fibromyalgia case–control GWASs were performed at deCODE Genetics/Amgen (Iceland, Denmark, US Intermountain Health and US NashBio) with software developed at deCODE Genetics, using logistic regression assuming an additive model^68^. For the Icelandic data, the model included sex, county of birth, current age or age at death (first- and second-order terms included), blood sample availability for the individual, sequencing status and an indicator function for the overlap of the lifetime of the individual with the time span of phenotype collection. To include imputed but ungenotyped individuals in Iceland, we used county of birth as a proxy covariate for the first PC because county of birth has been shown to be in concordance with the first PC in Iceland^79^. For the Danish data, 12 PCs were used, in addition to sex, year of birth and sequencing status as covariates, whereas for the Intermountain Health data, we included 4 PCs, year of birth, sex and sequencing status as covariates. For the NashBio data, associations were adjusted for the top 20 PCs in addition to sex, year of birth and sequencing batch. The number of PCs used to adjust for population stratification was determined by identifying the point at which further PCs appeared to capture local LD rather than population structure as reflected by sharp peaks in PC loadings and in plateauing of eigenvalues^80^. All statistical tests were two sided unless otherwise indicated.

### Harmonization and meta-analysis

Each cohort’s summary statistics were harmonized via an in-house analysis pipeline. For each summary statistics file (that is, for each cohort and ancestry), we removed variants with missing data in any column, non-ACGT alleles, *P* values outside (0, 1], allele frequencies outside (0, 1), imputation INFO scores outside (0, 1], or non-positive odds ratios, standard errors, or sample sizes. We normalized indels to their minimal representation by iteratively removing common trailing then leading base pairs from the reference and alternate alleles—subject to the constraint that each allele retains at least one base pair—and advancing the genomic position by the number of leading base pairs removed. We inferred Reference SNP cluster ID (rs) numbers based on chromosome and base-pair columns using dbSNP build 156, requiring an exact match to both the ref and the alt allele, allowing allele flips for single-nucleotide variants (but not indels) and converting the dbSNP variants to minimal representation as well before doing the matching. We removed variants without rs numbers in dbSNP, or that mapped to multiple genomic positions (this happens very rarely). To account for the tendency of dbSNP to merge rs numbers over time, if a variant mapped to multiple rs numbers, we took the lowest-numbered rs number. We calculated each cohort and ancestry’s effective sample size per variant via the formula *N*_eff_ = ((4/(2 × AAF × (1 − AAF) × INFO)) − BETA^2^)/SE^2^, skipping the ‘× INFO’ if INFO was not available, where INFO denotes the imputation information score, BETA the estimated log odds ratio for the variant, and SE its standard error. We then summed these *N*_eff_ across cohorts and ancestries.

To account for *P* value inflation due to residual confounding such as cryptic relatedness, we applied stratified LD score regression^81^ to each cohort and ancestry’s summary statistics before meta-analysis, using the effective sample size *N*_eff_ rather than the raw *N* to avoid bias^82^. We used the standard 52-annotation baseline model precomputed by the developers of stratified LD score regression. We manually calculated LD scores based on ancestry-specific reference panels from the corresponding 1000 Genomes Phase 3 superpopulation, where 1000 Genomes EUR was used for European cohorts, SAS (South Asian) for Central/South Asian cohorts, AFR for African cohorts, and AMR for admixed American cohorts. However, we subset to variants present in Europeans in HapMap 3, as the standard 52 annotations are available only for those variants. For cohort–ancestry pairs in which the LD score regression intercept was greater than 1, we divided each variant’s *χ*^2^ statistic by the LD score regression intercept, then adjusted the standard errors and *P* values accordingly. This has the effect of reducing the significance of every variant in summary statistics with evidence of inflation, while leaving non-inflated summary statistics alone.

After harmonization and LD score regression correction, we performed standard fixed-effects inverse-variance weighted meta-analyses via the ‘–meta-analysis’ option from plink version 1.9.0. Variants were matched across cohorts based on the combination of rs number, reference allele and alternate allele. In total, 54,629 cases and 2,509,126 controls went into the multi-ancestry meta-analysis, and 49,000 cases and 2,264,287 controls went into the European genetic ancestry-only analysis. We also performed sex-stratified meta-analyses restricted to males and females, and leave-one-cohort-out analyses for each of the 11 constituent cohorts, with both multi-ancestry (leaving out all ancestries from that cohort, if the cohort had multiple) and European-only versions. For females, we performed both European-only and multi-ancestry meta-analyses, but for males, we performed only the European-only meta-analysis owing to the lack of male summary statistics in Genes & Health and the low number of male cases in non-European ancestries in the multi-ancestry cohorts.

After meta-analysis, we filtered to variants with an overall minor allele frequency of >1% across all cohorts that went into that particular meta-analysis. As two cohorts (the UK Biobank and Mass General Brigham Biobank) reported variants in hg19 coordinates, we avoided having to perform an (error-prone) remapping of variant positions from hg19 to hg38 by filtering to variants present in at least 5 cohorts in the multi-ancestry meta-analyses and at least 3 studies in the European meta-analyses, thereby ensuring that every variant would be present in at least one hg38 study by the pigeonhole principle.

### Causal gene prioritization

We prioritized causal genes for each risk locus in the multi-ancestry meta-analysis via a combination of approaches. First, we performed manual literature search for each lead variant, focusing heavily on the nearest gene to each lead variant as nearest genes are expected to be causal about two-thirds of the time^83^. We supplemented this search with two orthogonal bioinformatic approaches: the state-of-the-art machine learning method FLAMES^84^ and an in-house quantitative trait locus (QTL)-based pipeline developed by deCODE Genetics.

#### FLAMES

FLAMES is a framework that nominates candidate causal genes at risk loci from a GWAS, via an ensemble of two complementary approaches.

First, a gradient boosting model scores genes based on locus-specific variant-to-gene evidence, aggregating features such as QTLs, variant effect predictor annotations, chromatin interactions and variant-to-gene distance. The model’s predictive weights were established by training it on external GWAS loci, in which ‘silver-standard’ causal genes had been identified via missense or predicted loss-of-function variants from exome studies. For this analysis, we supplied the model with 99% credible sets derived from the sum of single effects^85^ fine-mapping of variants within 500 kb of the lead variant at each locus from our primary meta-analysis.

Second, FLAMES incorporates gene-level scores from polygenic priority score (PoPS)^86^, another tool for causal gene prioritization. PoPS prioritizes genes by identifying gene features that are enriched for genetic association across the entire GWAS—such as pathway memberships, protein–protein interaction networks, co-expression data and expression in various tissues and cell types—then scoring each gene based on which of these enriched features it has. PoPS identifies these trait-relevant gene features by leveraging multi-marker analysis of genomic annotation^87^ to aggregate variant-level GWAS *P* values into gene-level association scores, then using a regression model to identify which gene features are predictive of high multi-marker analysis of genomic annotation scores.

FLAMES integrates these two evidence streams by multiplying the gradient boosting scores with scaled PoPS scores, effectively upweighting genes supported by both locus-specific and GWAS-wide evidence. At the recommended confidence threshold (scaled FLAMES score >0.248), FLAMES nominated a candidate causal gene at 19 of the 26 loci from the primary meta-analysis, while abstaining from prediction at the remaining 7 loci owing to a lack of confidence in the causal gene.

#### deCODE pipeline

As a second strategy to nominate candidate causal genes, we performed functional annotation and QTL analyses on all 26 lead variants from the primary meta-analysis, as well as all variants in high LD (*r*^2^ ≥ 0.8 and within ±1 Mb) with these lead variants.

We used variant effect predictor^88^ to attribute to the studied variants the most severe predicted variant consequences in canonical and non-canonical transcripts. We classified as high-impact variants those predicted as start-lost, stop-gained, stop-lost, splice-donor, splice-acceptor or frameshift, collectively called loss-of-function variants, whereas moderate impact variants are those predicted to otherwise affect coding or splicing of a protein (missense).

For all lead and correlated variants, we studied their association with (1) mRNA expression (top local expression quantitative trait locus (eQTL), splicing QTL or alternative polyadenylation QTL) in multiple tissues analyzed at deCODE, in addition to data from GTEx^89^ and other public datasets, and (2) plasma protein levels (top *cis*-protein QTL) identified in large proteomic datasets from Iceland and the UK^90^. RNA sequencing was performed on whole blood from 17,848 Icelanders and on subcutaneous adipose tissue from 769 Icelanders, respectively. Gene expression was computed based on personalized transcript abundances using kallisto^91^. The association between sequence variants and gene expression (*cis*-eQTL) was tested via linear regression, assuming additive genetic effect and normal quantile gene expression estimates, adjusting for measurements of sequencing artifacts, demographic variables, blood composition and PCs^92^. The gene expression PCs were computed per chromosome using a leave-one-chromosome-out method. All variants within 1 Mb of each gene were tested.

The Icelandic proteomics data were analyzed using the SomaLogic SOMAscan v4 proteomics assay that scans 4,907 aptamers, measuring 4,719 proteins in samples from 35,892 Icelanders with genetic information available at deCODE Genetics^90,93^. Plasma protein levels were standardized and adjusted for year of birth, sex and year of sample collection (2000–2019)^90,93^. The UK proteomics dataset was analyzed using the Olink Explore 3072 proximity extension assay platform with 2,941 immunoassays characterizing 2,925 proteins in 54,265 participants in the UK Biobank^90,93^.

### Phenome-wide associations of lead variants

For each of our 26 lead variants from the primary meta-analysis, we looked up genome-wide significant (*P* < 5 × 10^−8^) associations in the GWAS Catalog that overlapped either the variant itself or any proxy variants in high LD (*r*^2^ ≥ 0.8 and within ±1 Mb). To account for the GWAS Catalog containing large numbers of closely related phenotypes, we grouped related traits for visualization. Specifically, we removed GWAS results obtained through MTAG analysis or performed on multiple traits combined, identified by ‘and’ or ‘or’ in their trait names. We then standardized trait names by removing words in parentheses and laterality descriptors (‘left’ and ‘right’), and standardized the spelling of ‘neutrophill’ to ‘neurophil’. Finally, we grouped trait names that became identical after this standardization.

We also looked up the *P* values of our 26 lead variants in preexisting GWAS summary statistics for each of 330 disease endpoints from the November 2024 release of MVP–Finngen–UKBB (https://mvp-ukbb.finngen.fi/about), a GWAS meta-analysis of Million Veteran Program, FinnGen and the UK Biobank. We performed Bonferroni correction across the 330 diseases and 26 variants tested, leading to a significance threshold of *P* = 0.05/8,580 ≈ 5.83 × 10^−6^.

We applied the same approach to 124 GWAS of drug prescription endpoints from FinnGen’s DF12 release, in which each GWAS was on whether an individual was ever prescribed any drug from a particular category (for example, analgesics). We performed Bonferroni correction across the 124 drug categories and 26 variants tested, leading to a significance threshold of *P* = 0.05/3,224 ≈ 1.55 × 10^−5^.

### Heritability

For the European-only meta-analysis, we estimated single-nucleotide polymorphism-based heritability—the proportion of phenotypic variance explained by the aggregated effect of single-nucleotide polymorphisms across the autosomal genome—using stratified LD score regression^81^ with the standard 52-annotation baseline model mentioned above. We used LD scores computed from the European-ancestry subsample of 1000 Genomes Phase 3 (precomputed by LDSC authors), and subset to variants present in Europeans in HapMap 3. The observed-scale heritability was calculated from the LD score regression slope. As with all LD score regression-based analyses in this study, we used the effective sample size *N*_eff_ rather than the raw *N* to avoid bias due to case–control imbalance.

### Tissue and cell-type enrichment

We used LDSC-SEG^94^ to infer tissue and cell-type enrichments for the European-only meta-analysis. As for the global heritability analysis, we used LD scores computed from the European-ancestry subsample of 1000 Genomes Phase 3 (precomputed by LDSC authors) and the 52-annotation baseline model, subsetting to variants present in Europeans in HapMap 3. LDSC-SEG is a variant of stratified LD score regression that analyzes each tissue or cell type in turn, adding a binary annotation to the 52-annotation baseline model for each one. This annotation flags variants located within or within 100 kb of the 10% of genes most specifically expressed in that tissue or cell type. The heritability enrichment for each tissue or cell type is computed by dividing its per-SNP heritability (derived from the LD score regression slope) by the average per-SNP heritability across all SNPs in the analysis.

To compute tissue enrichments, we used annotations provided by the LDSC-SEG authors that were derived for each of 53 tissues in the GTEx project, in which the top 10% of specifically expressed genes were defined by ranking genes by their differential expression *t*-statistic for expression in that tissue versus all other tissues. For GTEx brain tissues, the comparison was instead between that tissue and all non-brain tissues.

To compute cell-type enrichments, we generated gene sets from a comprehensive single-cell transcriptomic atlas of the mouse, PanSci, which profiles over 20 million cells from 14 organs and tissues^95^. From this dataset, we included 119 cell types and 7 broad lineages that were represented by at least 1,000 cells after quality control (filtering to cells with ≤10% mitochondrial reads, ≥100 genes detected and non-zero Malat1 expression). Our analysis was restricted to a universe of 16,404 protein-coding genes with identifiable human orthologs. For each cell type and lineage, we defined its specifically expressed genes as those with a detection rate >10% within that cell type and a fold change >2 compared with all other cells.

### Genetic correlation

We performed genetic correlation between our leave-FinnGen-out European-only fibromyalgia meta-analysis and each of 1,284 clinical endpoints from FinnGen Release 13 with https://www.finngen.fi/en/researchers/clinical-endpoints, using LD score regression^96^. We calculated SNP heritability with LDSC for all 2,691 endpoints and performed genetic correlation only on those endpoints with significant heritability, resulting in a list of 1,284 endpoints. Variants were aligned and filtered to HapMap release 3 SNPs, and the LD reference was obtained from the European population of the 1000 Genomes Project Phase 3 release. We used the 80th percentile of per-variant effective sample sizes (*N*_eff_) to munge the summary statistics.

To avoid diluting the results with low-specificity phenotypes, we then excluded endpoints containing any of the (case-insensitive) keywords ‘other’ (for example, ‘Other disorders of ear’), ‘unspecified’ (for example, ‘Disorder of external ear, unspecified’), classified (for example, ‘Other viral diseases, not elsewhere classified’), ‘any’ (for example, ‘Any mental disorder’) or ‘all’ (for example, ‘All kidney diseases’), as well as derived endpoints for which the listed category did not correspond to a chapter of the ICD. We also excluded fibromyalgia itself as an endpoint. These exclusions reduced the number of endpoints tested from 1,284 to 855, resulting in a Bonferroni significance threshold of *P* = 0.05/855 ≈ 5.85 × 10^−5^.

For visualization purposes, we grouped endpoints by their ICD chapter. We grouped four categories with few significant endpoints—‘III Diseases of the blood and blood-forming organs and certain disorders involving the immune mechanism’, ‘XVIII Symptoms, signs and abnormal clinical and laboratory findings, not elsewhere classified’, ‘XXI Factors influencing health status and contact with health services’ and ‘XXII Codes for special purposes’—into an ‘Other’ category. Because fibromyalgia has been previously proposed to share etiology with autoimmune disorders, we also created a separate ‘Autoimmune’ category, as these disorders would otherwise be scattered across ICD chapters based on the affected organ system.

We also estimated the genetic correlation between fibromyalgia and the subtypes of asthma and RA using summary statistics available at deCODE. In line with other LDSC-based analyses, we used per-variant effective sample sizes, filtered variants to HapMap release 3 SNPs and used precomputed LD scores for European populations (downloaded from https://data.broadinstitute.org/alkesgroup/LDSCORE/eur_w_ld_chr.tar.bz2).

### PRSs

We computed PRSs for the leave-UK Biobank-out European and multi-ancestry meta-analyses, using PRS-CS^97^. PRS-CS takes GWAS summary statistics and an LD reference panel as input, and applies Bayesian shrinkage to the variants’ effect sizes to infer posterior effect sizes, which are used as the weights of the PRS. These weights can then be scored on any cohort of interest, which should be non-overlapping with the cohorts used to create the PRS to avoid bias.

We used the European-ancestry subsample of 1000 Genomes Phase 3 as the reference panel, removing indels from the summary statistics before the analysis. We ran PRS-CS with the ‘–n_burnin 5000’ and ‘–n_iter 10000’ options to specify 5,000 burn-in iterations and 10,000 total iterations of the Markov Chain Monte Carlo sampling. We then scored the PRS weights on the UK Biobank cohort with the ‘–score’ option from plink version 2.0.0. As PRS-CS requires a single sample size across all variants, the sample size we provided to PRS-CS was the 80th percentile of per-variant effective sample sizes (*N*_eff_), as recommended by others for PRS analyses^98^.

To assess the accuracy of the PRS across ancestries, we scored the PRS weights from the multi-ancestry leave-UK Biobank-out meta-analysis on each of the 3 largest genetic ancestries from the UK Biobank (European, Central and South Asian, and African). We assessed PRS accuracy via the AUC, as well as by computing odds ratios for each quintile of polygenic risk relative to the middle quintile, and prevalence of fibromyalgia within each quintile.

### Statistics and reproducibility

No statistical method was used to predetermine sample size. No data were excluded from the analyses. The experiments were not randomized. The investigators were not blinded to allocation during experiments and outcome assessment.

### Reporting summary

Further information on research design is available in the Nature Portfolio Reporting Summary linked to this article.

## Online content

Any methods, additional references, Nature Portfolio reporting summaries, source data, extended data, supplementary information, acknowledgements, peer review information; details of author contributions and competing interests; and statements of data and code availability are available at 10.1038/s41591-026-04492-6.

## Supplementary information

Supplementary Information Supplementary Figs. 1–3.

Reporting Summary

Supplementary Tables Supplementary Table 1: Case and control sample sizes in full-cohort analysis stratified by cohort, ancestry and sex. Supplementary Table 2: Lead variants and candidate causal genes from sex-specific meta-analyses. Supplementary Table 3: Candidate causal genes prioritized through functional annotations and identification of coding variants in high linkage disequilibrium with lead variants. Supplementary Table 4: GWAS Catalog associations for lead variants and variants in high LD with lead variants. Supplementary Table 5: Disease PheWAS associations of lead variants queried from MVP + FinnGen + UKBB meta-analysis summary statistics. Supplementary Table 6: Drug PheWAS associations of lead variants queried from FinnGen summary statistics. Supplementary Table 7: Tissue enrichment results from LDSC-SEG analysis. Supplementary Table 8: Cell-type enrichment results from LDSC-SEG analysis of main mouse cell types. Supplementary Table 9: Cell-type enrichment results from LDSC-SEG analysis of mouse cell lineages. Supplementary Table 10: Genetic correlation results between fibromyalgia and FinnGen disease endpoints. Supplementary Table 11: Overview of QC parameters and phenotype definition per cohort.

## Data Availability

GWAS summary statistics are available in the GWAS Catalog (accession codes GCST90838603 to GCST90838628) and PRSs are available in the PGS catalog (score IDs PGS012560 and PGS012561). All summary statistics and PRSs are also available at https://paingenomics.org. This study used data from the All of Us Research Program’s Controlled Tier Dataset Curated Data Repository version 8, available to authorized users on the Researcher Workbench.

## Code availability

The code is available via GitHub at https://github.com/i-kerrebijn/fibromyalgia_GWAS_meta-analysis.
