# Supplementary material for: The genetic architecture of fibromyalgia across 2.5 million individuals
Source: Nat Med. 2026 Jul 28;32(8):3060–70. doi: 10.1038/s41591-026-04492-6 (PMC13472937; doi:10.1038/s41591-026-04492-6)
Supplement: Supplementary file 1 [file 41591_2026_4492_MOESM1_ESM.pdf]

nature medicine

Supplementary information https://doi.org/10.1038/s41591-026-04492-6

The genetic architecture of fibromyalgia across 2.5 million individuals

In the format provided by the authors and unedited

Supplementary Figures

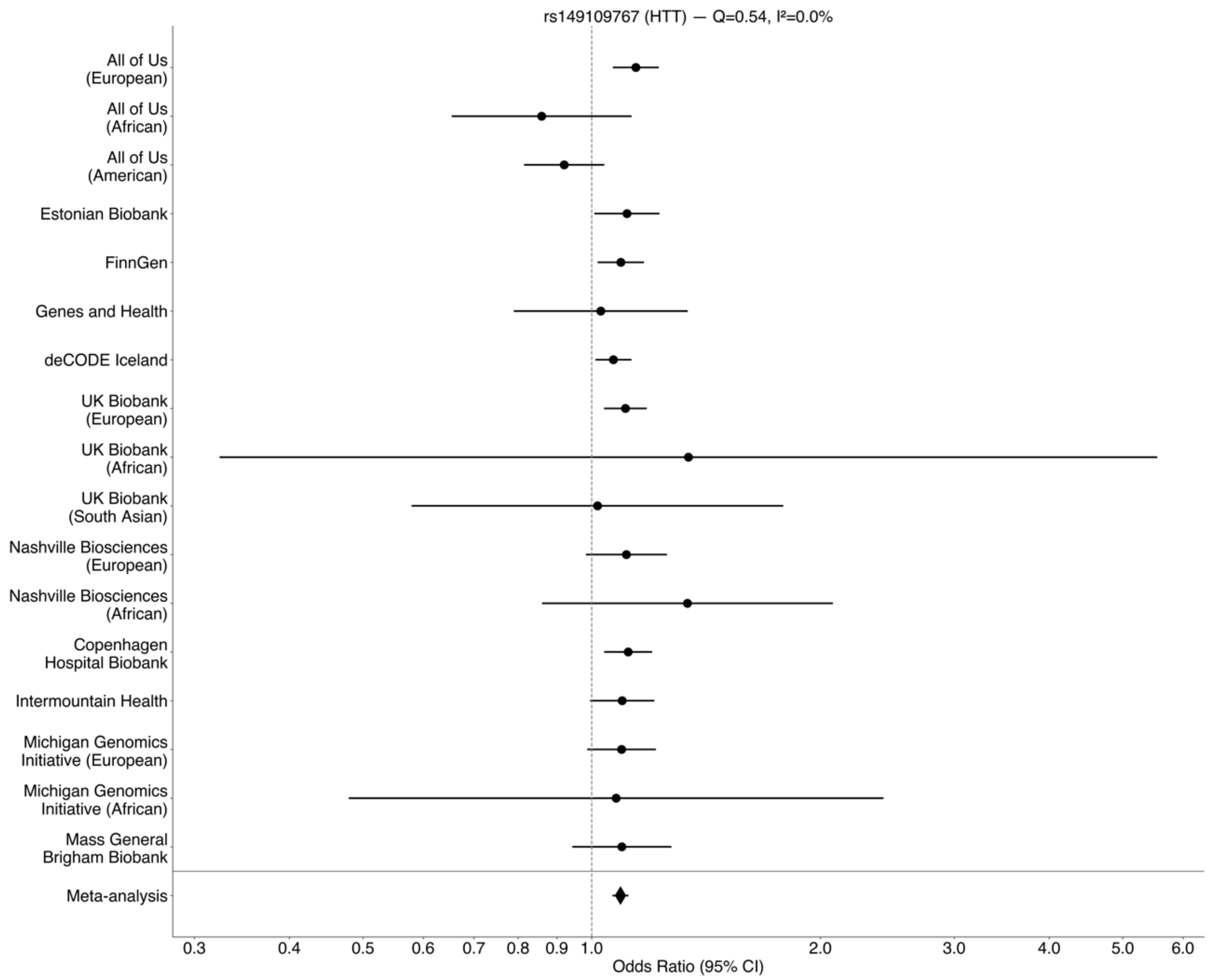

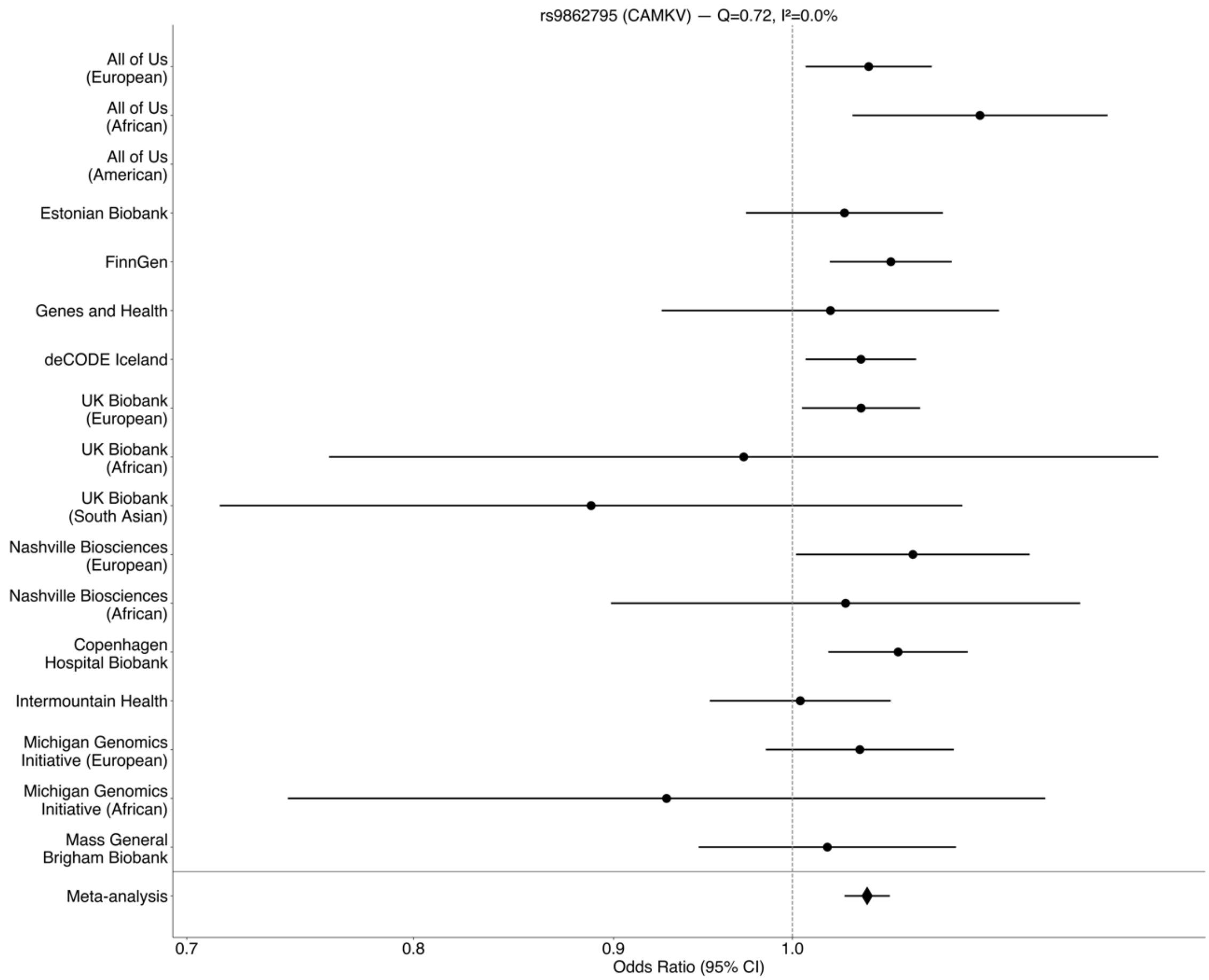

rs9862795 (CAMKV) — Q=0.72, I²=0.0%
All of Us (European)
All of Us (African)
All of Us (American)
Estonian Biobank
FinnGen
Genes and Health
deCODE Iceland
UK Biobank (European)
UK Biobank (African)
UK Biobank (South Asian)
Nashville Biosciences (European)
Nashville Biosciences (African)
Copenhagen Hospital Biobank
Intermountain Health
Michigan Genomics Initiative (European)
Michigan Genomics Initiative (African)
Mass General Brigham Biobank
Meta-analysis
0.7
0.8
0.9
1.0
Odds Ratio (95% CI)

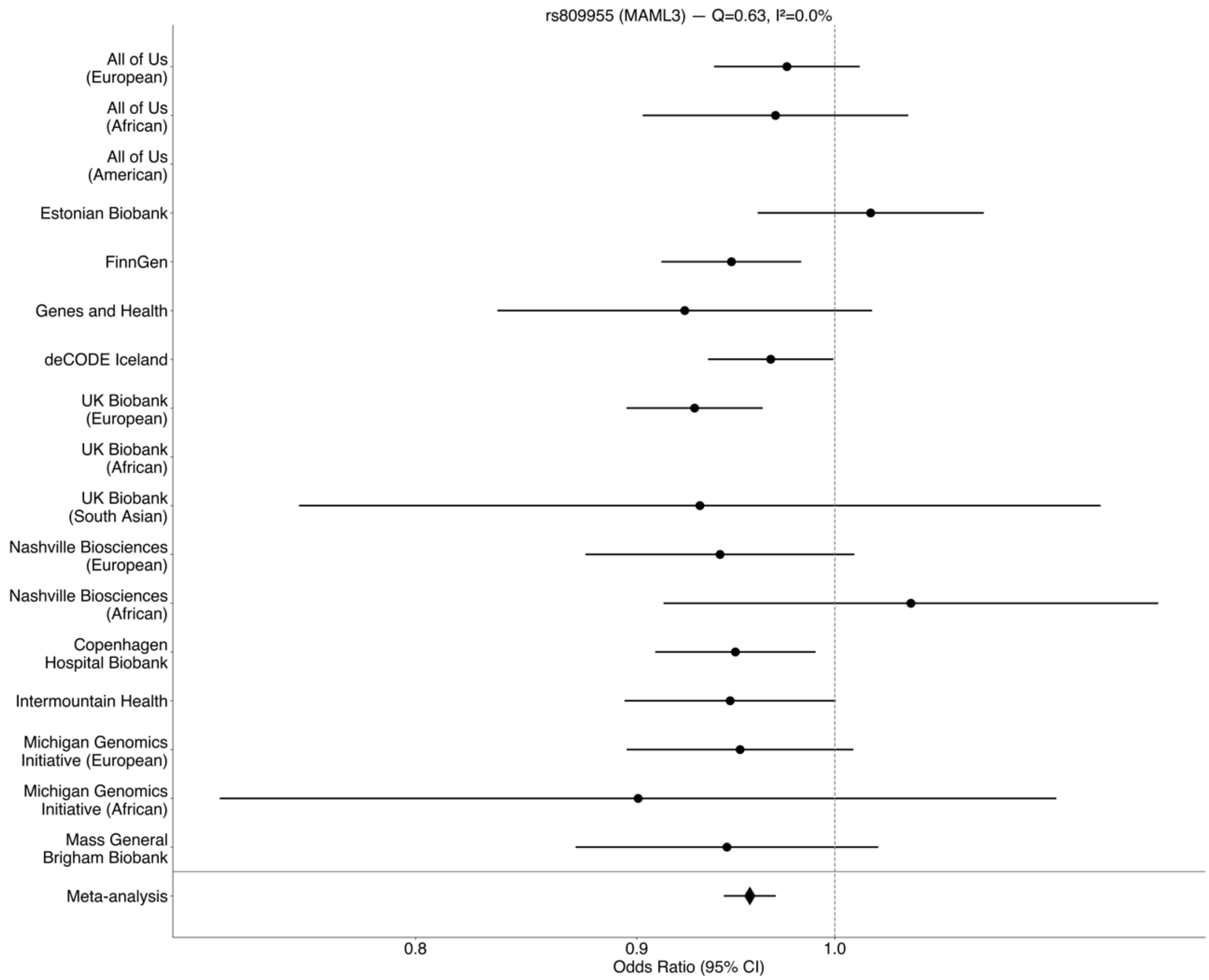
rs809955 (MAML3) — Q=0.63, I²=0.0%
All of Us (European)
All of Us (African)
All of Us (American)
Estonian Biobank
FinnGen
Genes and Health
deCODE Iceland
UK Biobank (European)
UK Biobank (African)
UK Biobank (South Asian)
Nashville Biosciences (European)
Nashville Biosciences (African)
Copenhagen Hospital Biobank
Intermountain Health
Michigan Genomics Initiative (European)
Michigan Genomics Initiative (African)
Mass General Brigham Biobank
Meta-analysis
0.8
0.9
1.0
Odds Ratio (95% CI)

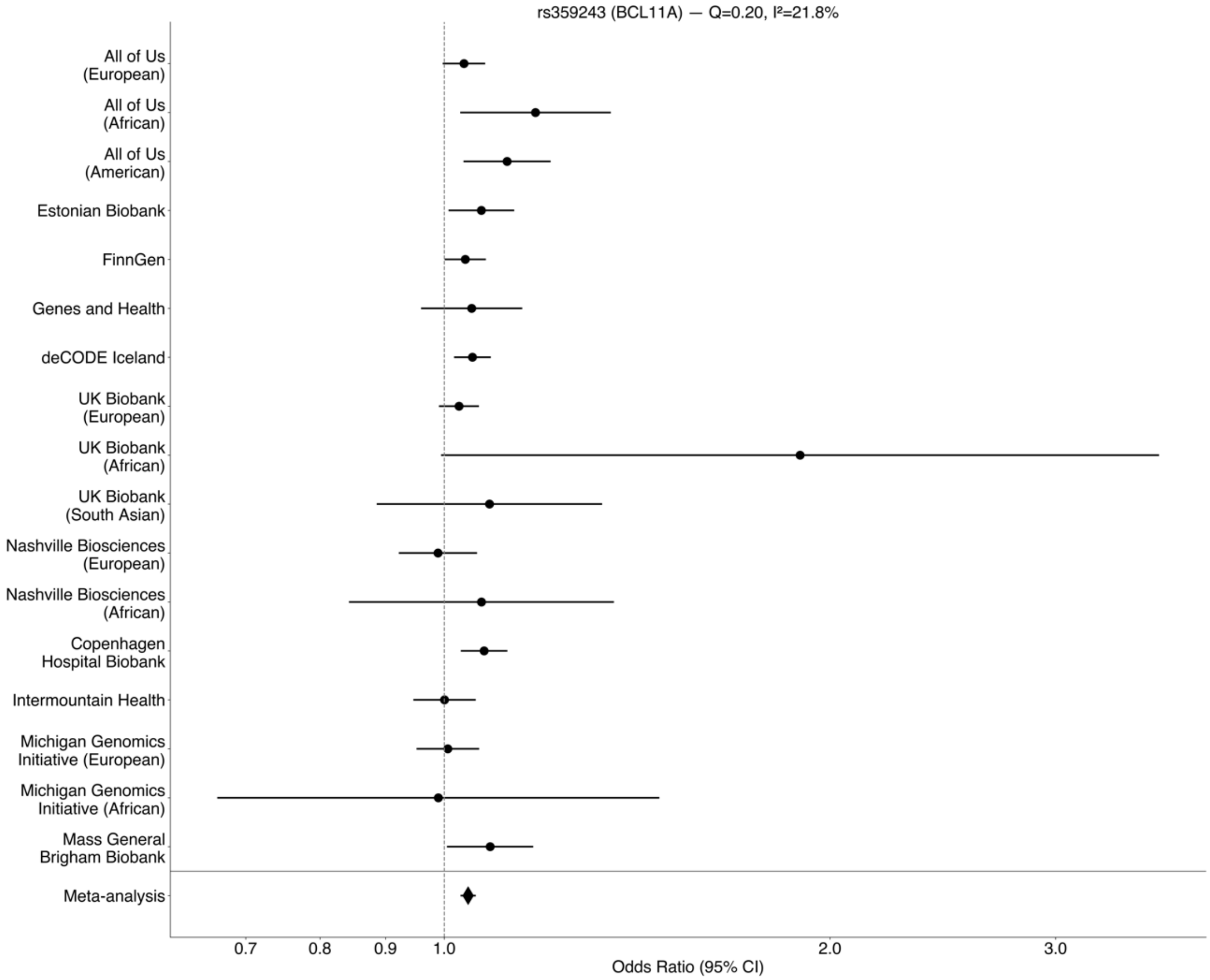

rs359243 (BCL11A) — Q=0.20, I²=21.8%
All of Us (European)
All of Us (African)
All of Us (American)
Estonian Biobank
FinnGen
Genes and Health
deCODE Iceland
UK Biobank (European)
UK Biobank (African)
UK Biobank (South Asian)
Nashville Biosciences (European)
Nashville Biosciences (African)
Copenhagen Hospital Biobank
Intermountain Health
Michigan Genomics Initiative (European)
Michigan Genomics Initiative (African)
Mass General Brigham Biobank
Meta-analysis
0.7
0.8
0.9
1.0
2.0
3.0
Odds Ratio (95% CI)

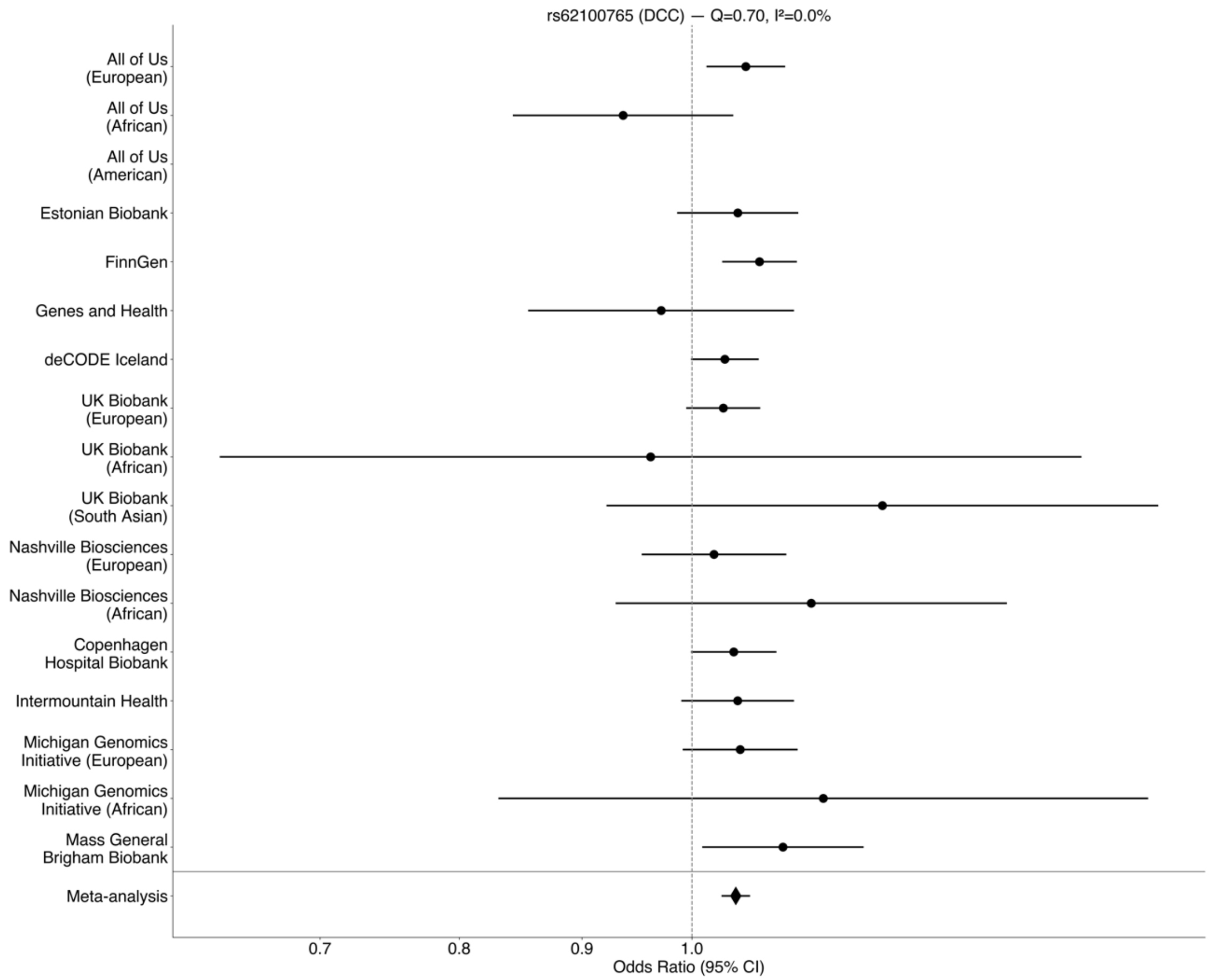

rs62100765 (DCC) — Q=0.70, I²=0.0%
All of Us (European)
All of Us (African)
All of Us (American)
Estonian Biobank
FinnGen
Genes and Health
deCODE Iceland
UK Biobank (European)
UK Biobank (African)
UK Biobank (South Asian)
Nashville Biosciences (European)
Nashville Biosciences (African)
Copenhagen Hospital Biobank
Intermountain Health
Michigan Genomics Initiative (European)
Michigan Genomics Initiative (African)
Mass General Brigham Biobank
Meta-analysis
0.7
0.8
0.9
1.0
Odds Ratio (95% CI)

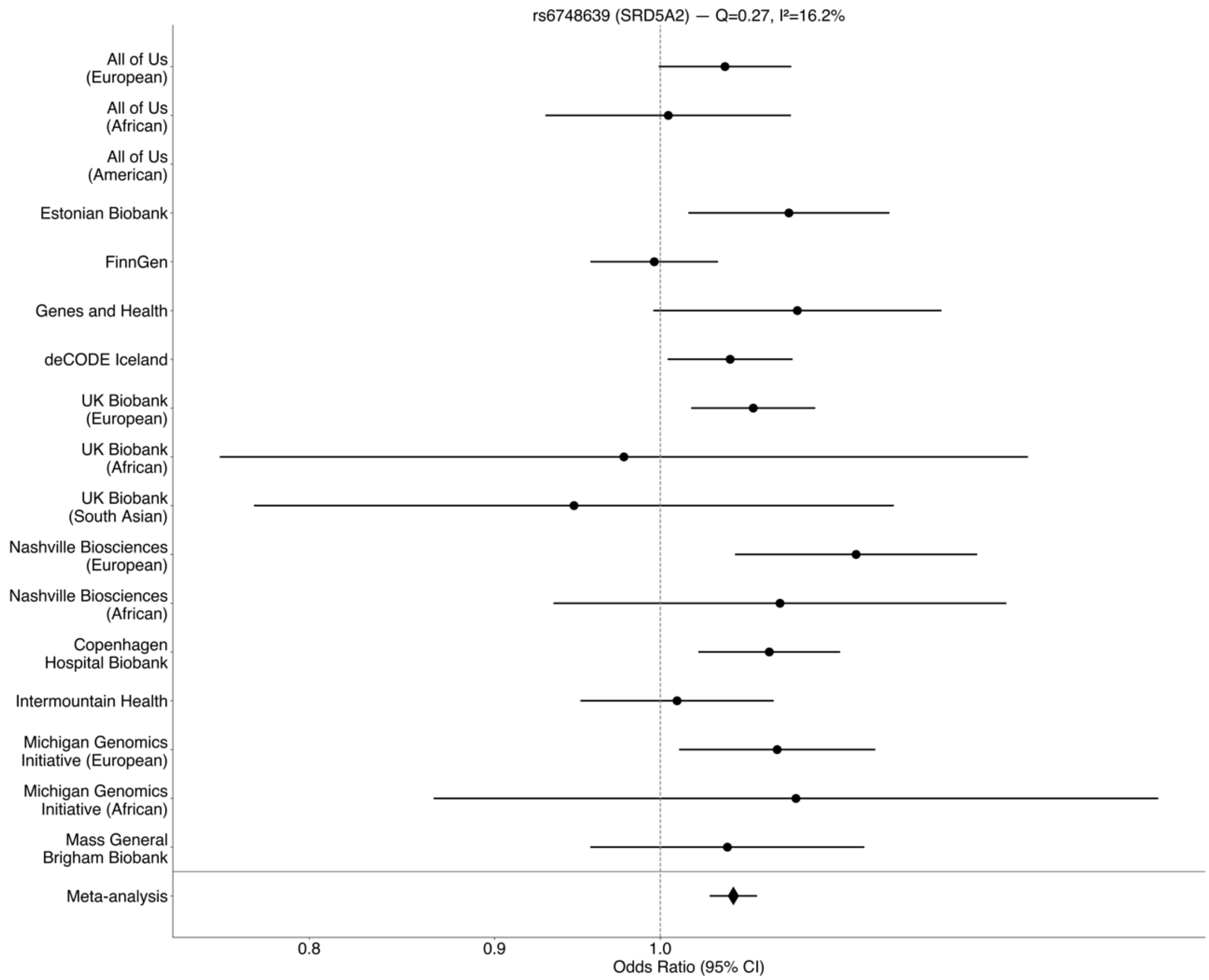

rs6748639 (SRD5A2) — Q=0.27, I²=16.2%
All of Us (European)
All of Us (African)
All of Us (American)
Estonian Biobank
FinnGen
Genes and Health
deCODE Iceland
UK Biobank (European)
UK Biobank (African)
UK Biobank (South Asian)
Nashville Biosciences (European)
Nashville Biosciences (African)
Copenhagen Hospital Biobank
Intermountain Health
Michigan Genomics Initiative (European)
Michigan Genomics Initiative (African)
Mass General Brigham Biobank
Meta-analysis
0.8
0.9
1.0
Odds Ratio (95% CI)

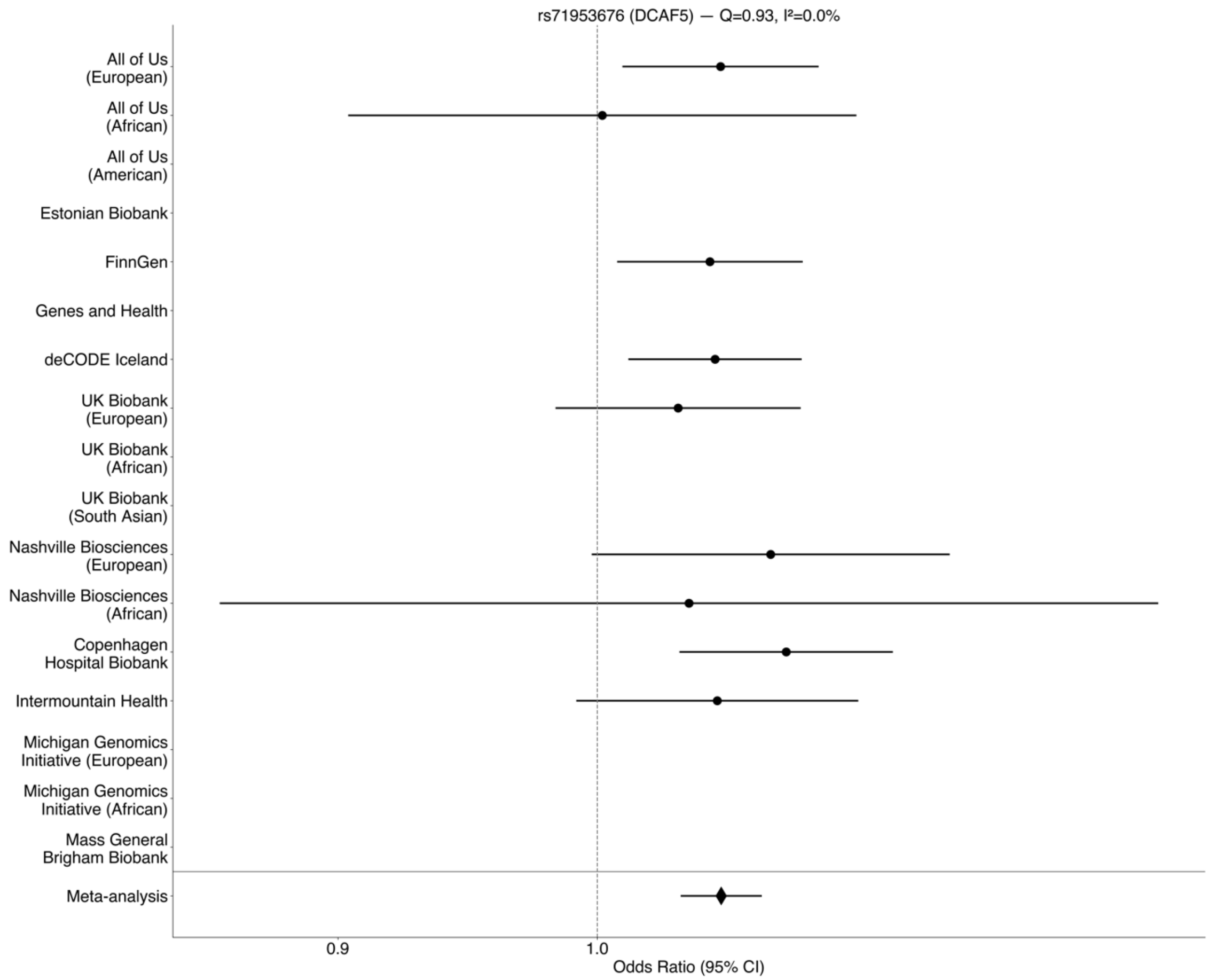

rs71953676 (DCAF5) — Q=0.93, I²=0.0%
All of Us (European)
All of Us (African)
All of Us (American)
Estonian Biobank
FinnGen
Genes and Health
deCODE Iceland
UK Biobank (European)
UK Biobank (African)
UK Biobank (South Asian)
Nashville Biosciences (European)
Nashville Biosciences (African)
Copenhagen Hospital Biobank
Intermountain Health
Michigan Genomics Initiative (European)
Michigan Genomics Initiative (African)
Mass General Brigham Biobank
Meta-analysis
0.9
1.0
Odds Ratio (95% CI)

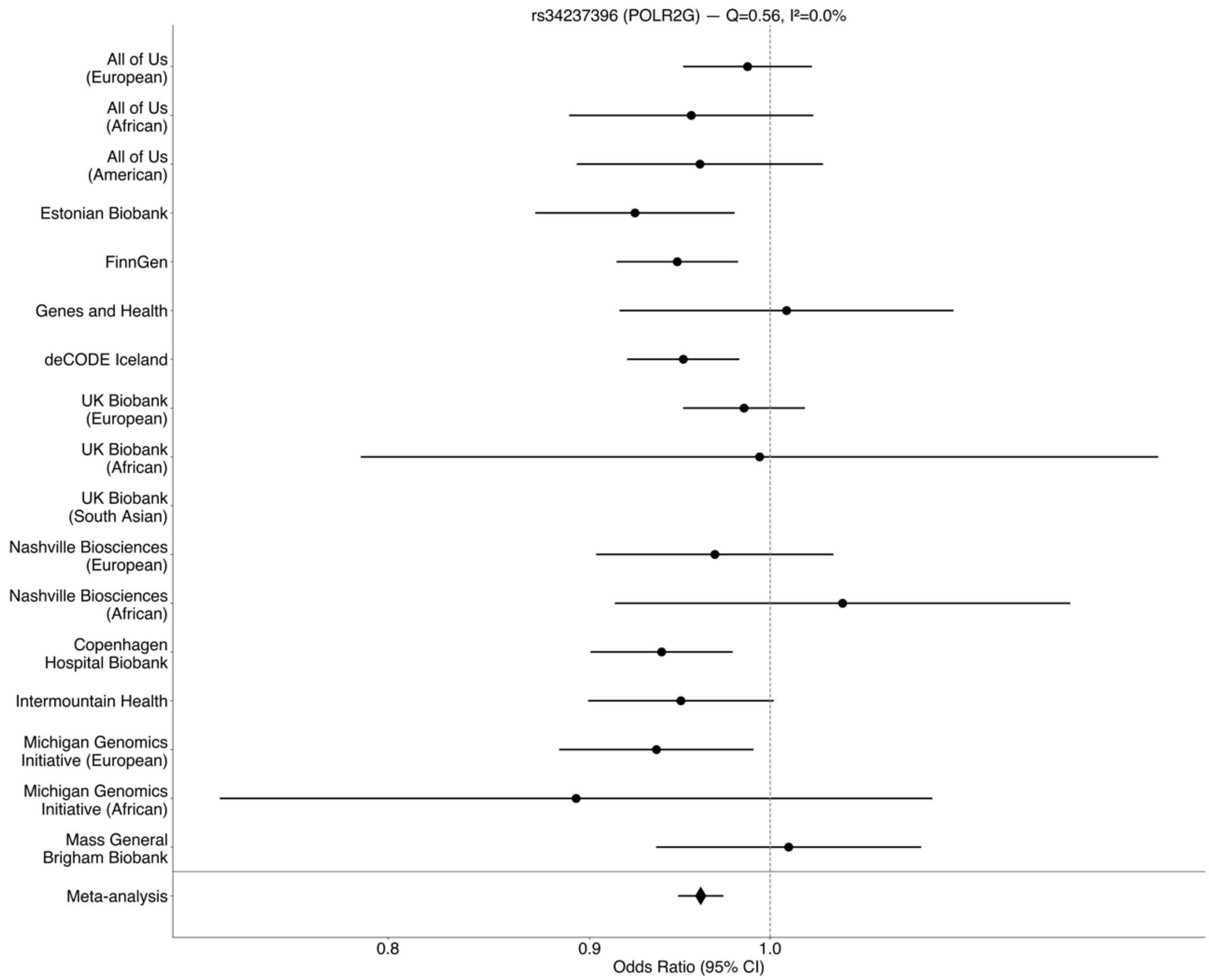
rs34237396 (POLR2G) — Q=0.56, I²=0.0%
All of Us (European)
All of Us (African)
All of Us (American)
Estonian Biobank
FinnGen
Genes and Health
deCODE Iceland
UK Biobank (European)
UK Biobank (African)
UK Biobank (South Asian)
Nashville Biosciences (European)
Nashville Biosciences (African)
Copenhagen Hospital Biobank
Intermountain Health
Michigan Genomics Initiative (European)
Michigan Genomics Initiative (African)
Mass General Brigham Biobank
Meta-analysis
0.8
0.9
1.0
Odds Ratio (95% CI)

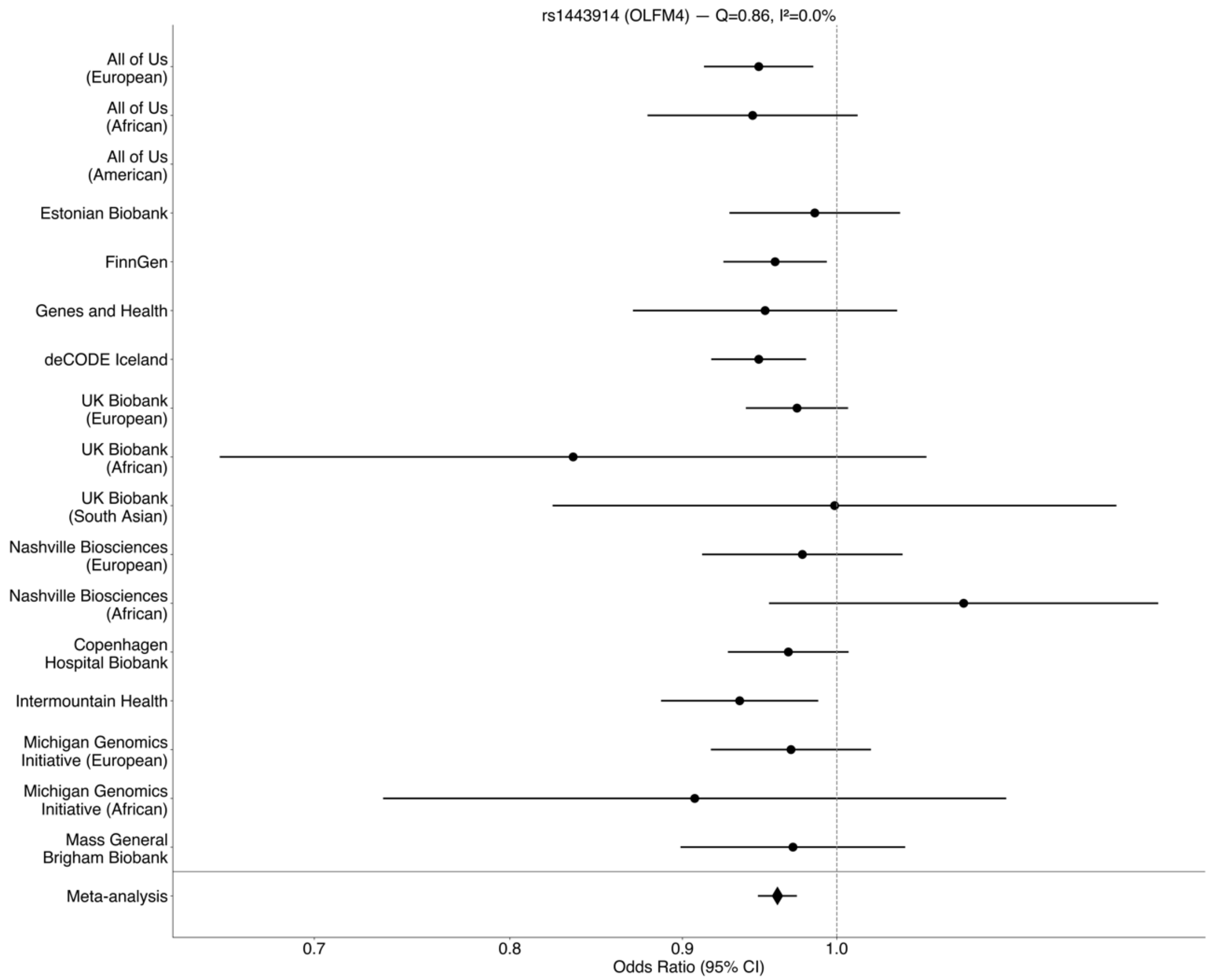
rs1443914 (OLFM4) — Q=0.86, I²=0.0%
All of Us (European)
All of Us (African)
All of Us (American)
Estonian Biobank
FinnGen
Genes and Health
deCODE Iceland
UK Biobank (European)
UK Biobank (African)
UK Biobank (South Asian)
Nashville Biosciences (European)
Nashville Biosciences (African)
Copenhagen Hospital Biobank
Intermountain Health
Michigan Genomics Initiative (European)
Michigan Genomics Initiative (African)
Mass General Brigham Biobank
Meta-analysis
0.7
0.8
0.9
1.0
Odds Ratio (95% CI)

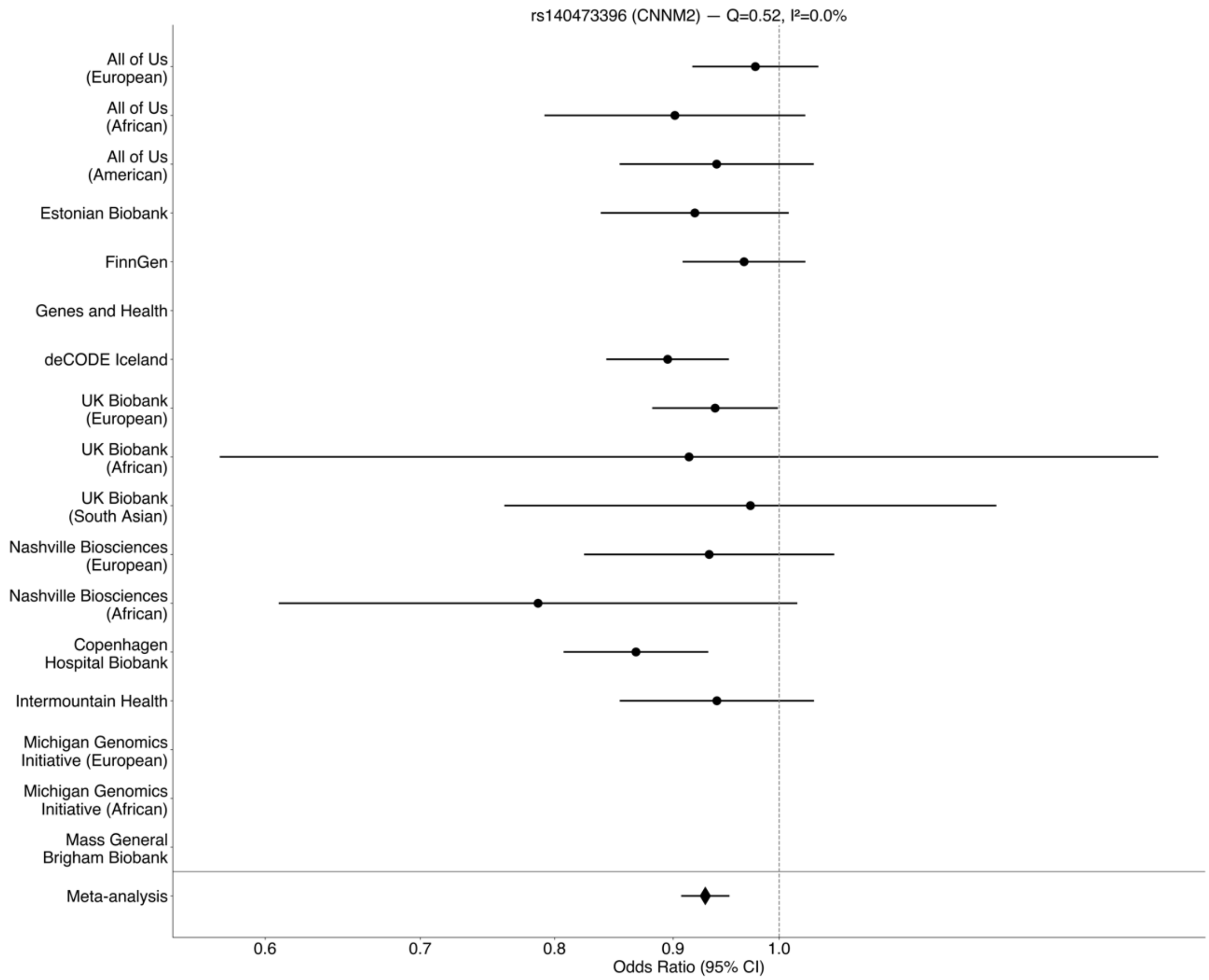
rs140473396 (CNNM2) — Q=0.52, I²=0.0%
All of Us (European)
All of Us (African)
All of Us (American)
Estonian Biobank
FinnGen
Genes and Health
deCODE Iceland
UK Biobank (European)
UK Biobank (African)
UK Biobank (South Asian)
Nashville Biosciences (European)
Nashville Biosciences (African)
Copenhagen Hospital Biobank
Intermountain Health
Michigan Genomics Initiative (European)
Michigan Genomics Initiative (African)
Mass General Brigham Biobank
Meta-analysis
0.6
0.7
0.8
0.9
1.0
Odds Ratio (95% CI)

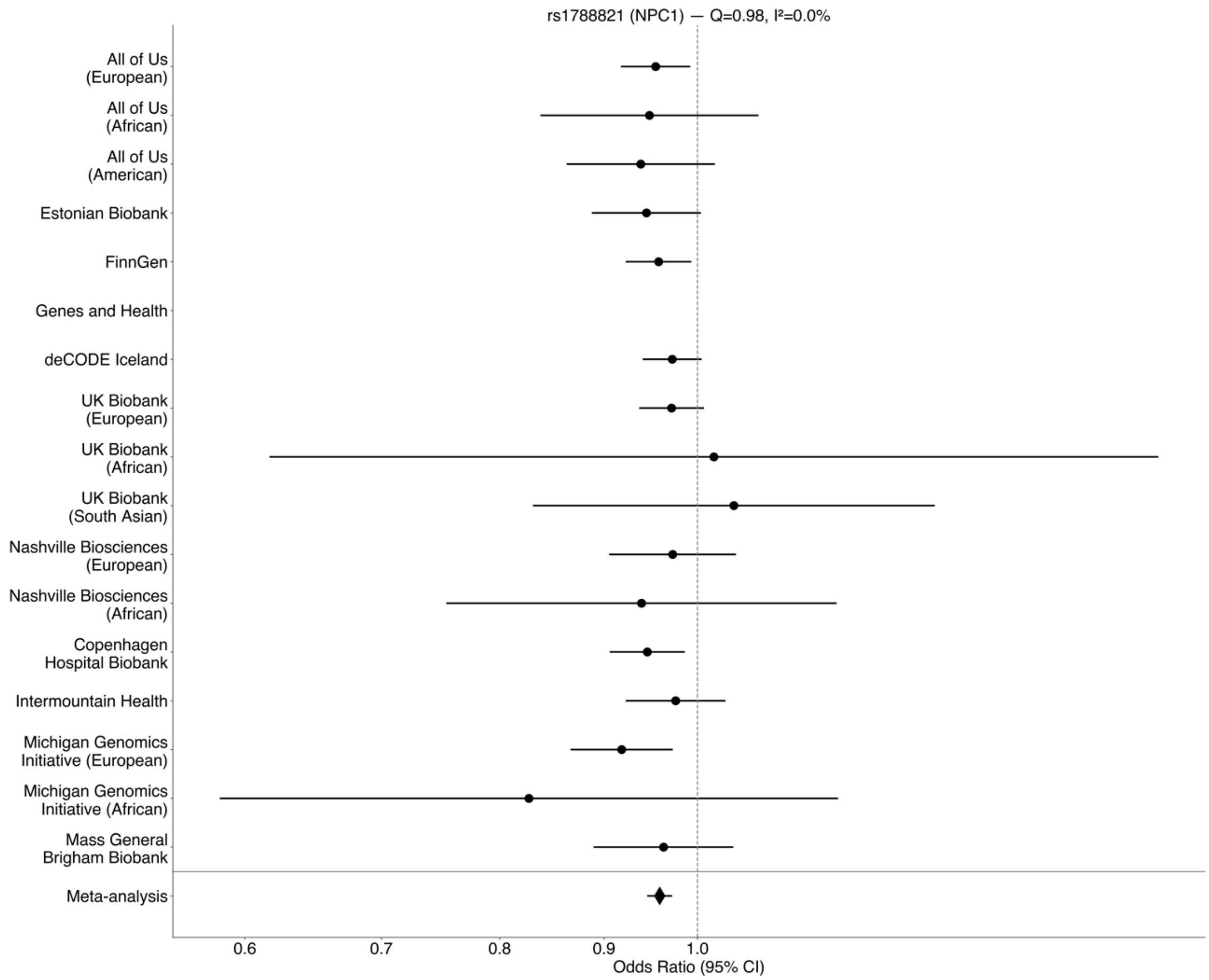
rs1788821 (NPC1) — Q=0.98, I²=0.0%
All of Us (European)
All of Us (African)
All of Us (American)
Estonian Biobank
FinnGen
Genes and Health
deCODE Iceland
UK Biobank (European)
UK Biobank (African)
UK Biobank (South Asian)
Nashville Biosciences (European)
Nashville Biosciences (African)
Copenhagen Hospital Biobank
Intermountain Health
Michigan Genomics Initiative (European)
Michigan Genomics Initiative (African)
Mass General Brigham Biobank
Meta-analysis
0.6
0.7
0.8
0.9
1.0
Odds Ratio (95% CI)

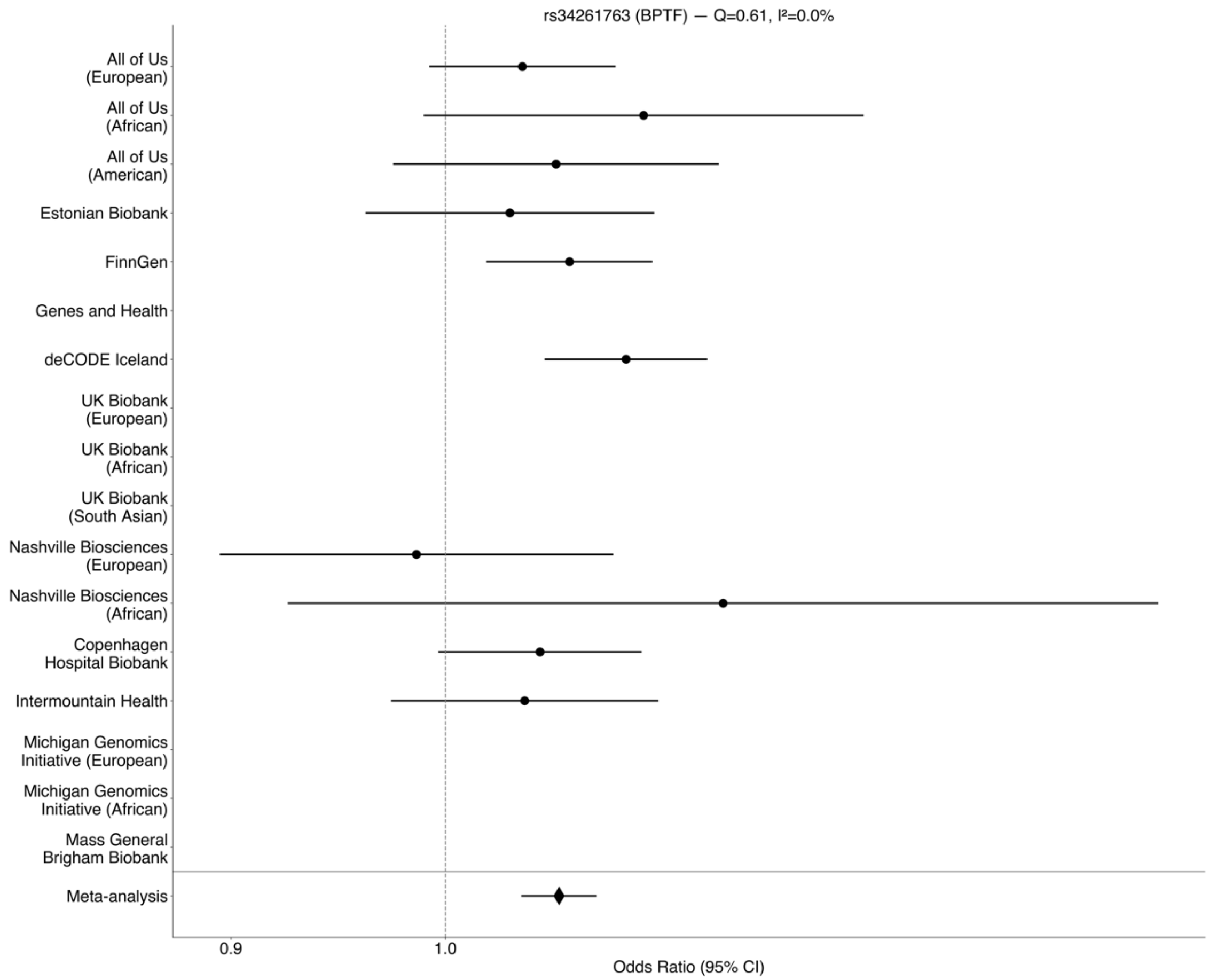
rs34261763 (BPTF) — Q=0.61, I²=0.0%
All of Us (European)
All of Us (African)
All of Us (American)
Estonian Biobank
FinnGen
Genes and Health
deCODE Iceland
UK Biobank (European)
UK Biobank (African)
UK Biobank (South Asian)
Nashville Biosciences (European)
Nashville Biosciences (African)
Copenhagen Hospital Biobank
Intermountain Health
Michigan Genomics Initiative (European)
Michigan Genomics Initiative (African)
Mass General Brigham Biobank
Meta-analysis
0.9
1.0
Odds Ratio (95% CI)

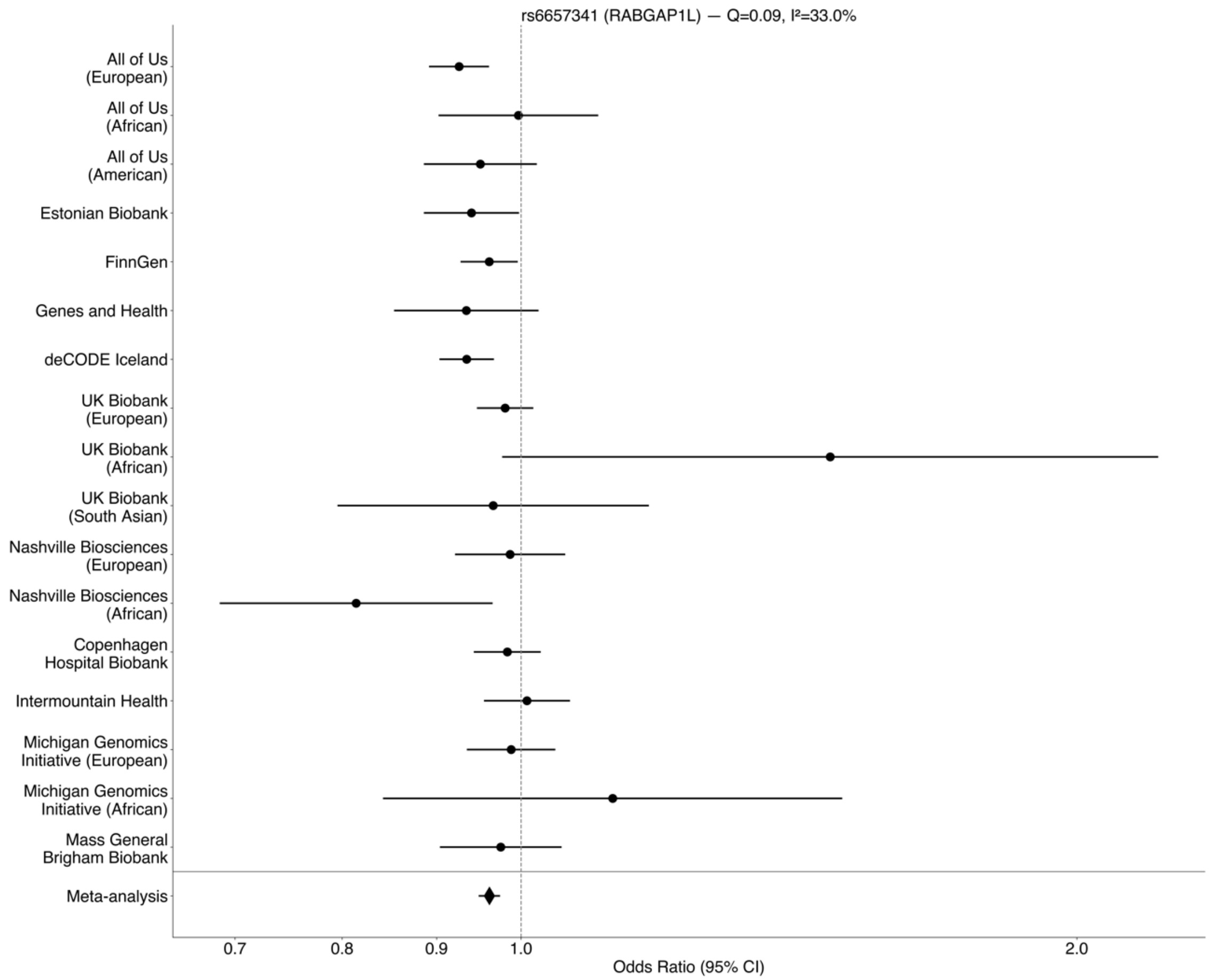
rs6657341 (RABGAP1L) — Q=0.09, I²=33.0%
All of Us (European)
All of Us (African)
All of Us (American)
Estonian Biobank
FinnGen
Genes and Health
deCODE Iceland
UK Biobank (European)
UK Biobank (African)
UK Biobank (South Asian)
Nashville Biosciences (European)
Nashville Biosciences (African)
Copenhagen Hospital Biobank
Intermountain Health
Michigan Genomics Initiative (European)
Michigan Genomics Initiative (African)
Mass General Brigham Biobank
Meta-analysis
0.7
0.8
0.9
1.0
2.0
Odds Ratio (95% CI)

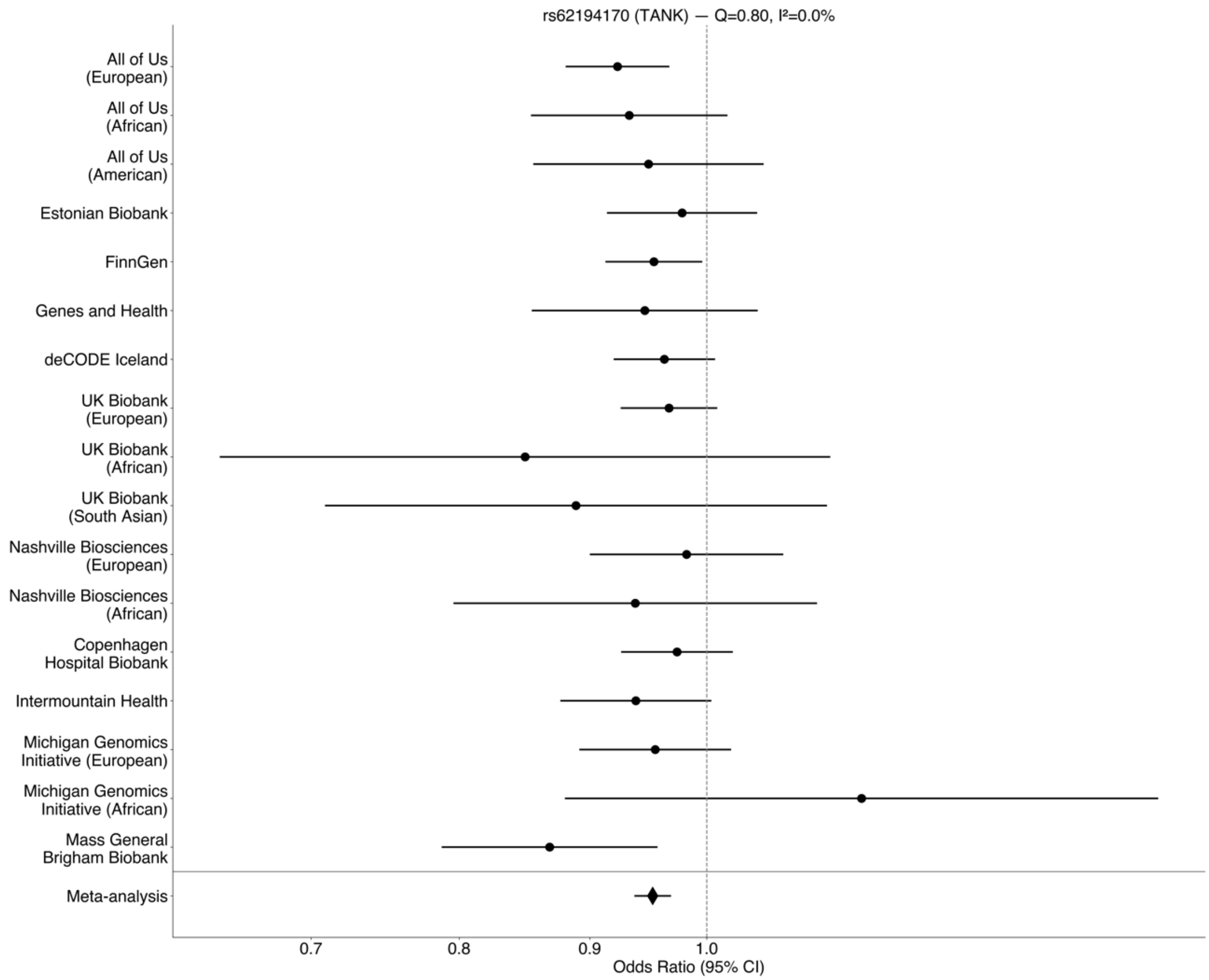

rs62194170 (TANK) — Q=0.80, I²=0.0%
All of Us (European)
All of Us (African)
All of Us (American)
Estonian Biobank
FinnGen
Genes and Health
deCODE Iceland
UK Biobank (European)
UK Biobank (African)
UK Biobank (South Asian)
Nashville Biosciences (European)
Nashville Biosciences (African)
Copenhagen Hospital Biobank
Intermountain Health
Michigan Genomics Initiative (European)
Michigan Genomics Initiative (African)
Mass General Brigham Biobank
Meta-analysis
0.7
0.8
0.9
1.0
Odds Ratio (95% CI)

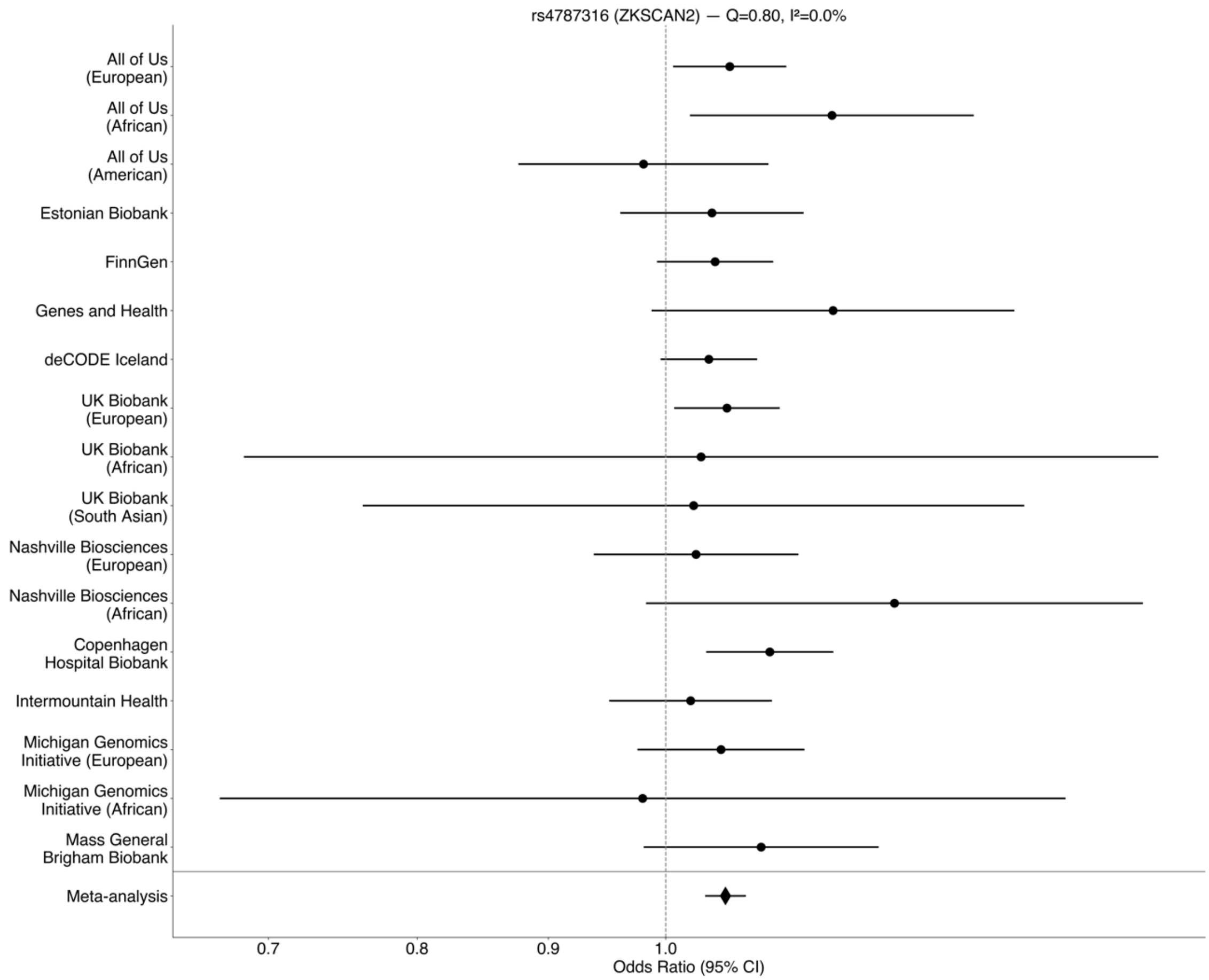
rs4787316 (ZKSCAN2) — Q=0.80, I²=0.0%
All of Us (European)
All of Us (African)
All of Us (American)
Estonian Biobank
FinnGen
Genes and Health
deCODE Iceland
UK Biobank (European)
UK Biobank (African)
UK Biobank (South Asian)
Nashville Biosciences (European)
Nashville Biosciences (African)
Copenhagen Hospital Biobank
Intermountain Health
Michigan Genomics Initiative (European)
Michigan Genomics Initiative (African)
Mass General Brigham Biobank
Meta-analysis
0.7
0.8
0.9
1.0
Odds Ratio (95% CI)

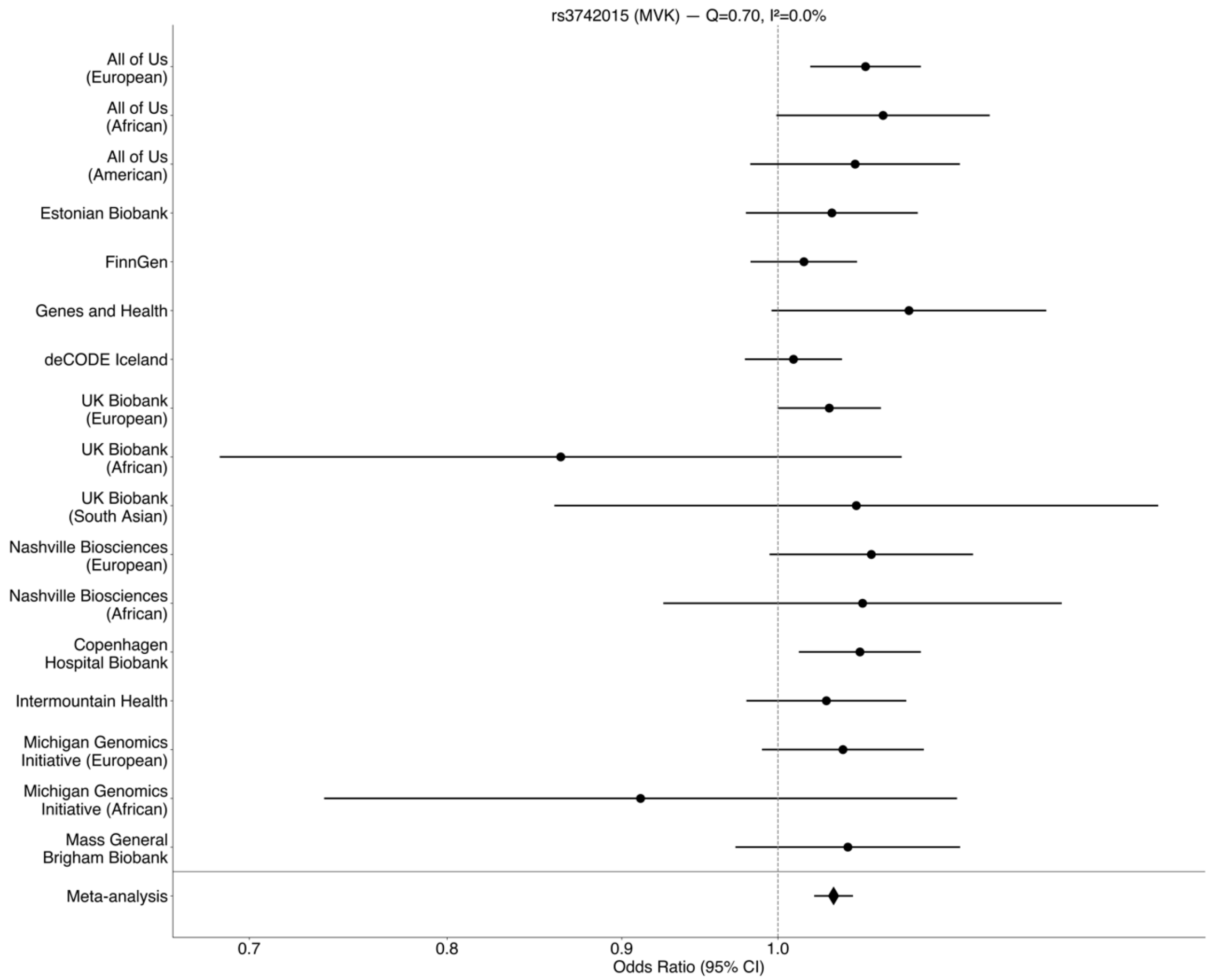
rs3742015 (MVK) — Q=0.70, I²=0.0%
All of Us (European)
All of Us (African)
All of Us (American)
Estonian Biobank
FinnGen
Genes and Health
deCODE Iceland
UK Biobank (European)
UK Biobank (African)
UK Biobank (South Asian)
Nashville Biosciences (European)
Nashville Biosciences (African)
Copenhagen Hospital Biobank
Intermountain Health
Michigan Genomics Initiative (European)
Michigan Genomics Initiative (African)
Mass General Brigham Biobank
Meta-analysis
0.7
0.8
0.9
1.0
Odds Ratio (95% CI)

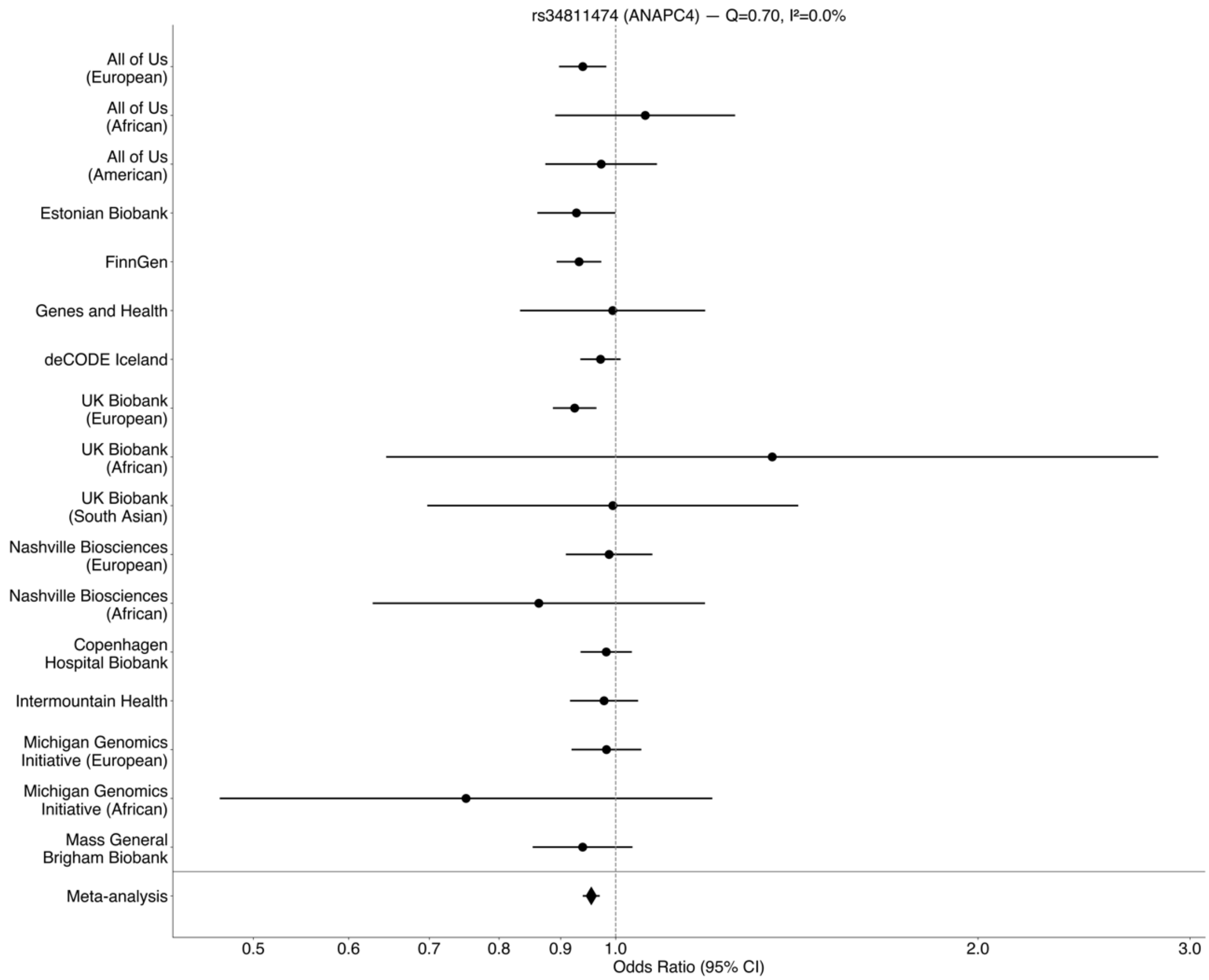
rs34811474 (ANAPC4) — Q=0.70, I²=0.0%
All of Us (European)
All of Us (African)
All of Us (American)
Estonian Biobank
FinnGen
Genes and Health
deCODE Iceland
UK Biobank (European)
UK Biobank (African)
UK Biobank (South Asian)
Nashville Biosciences (European)
Nashville Biosciences (African)
Copenhagen Hospital Biobank
Intermountain Health
Michigan Genomics Initiative (European)
Michigan Genomics Initiative (African)
Mass General Brigham Biobank
Meta-analysis
0.5
0.6
0.7
0.8
0.9
1.0
2.0
3.0
Odds Ratio (95% CI)

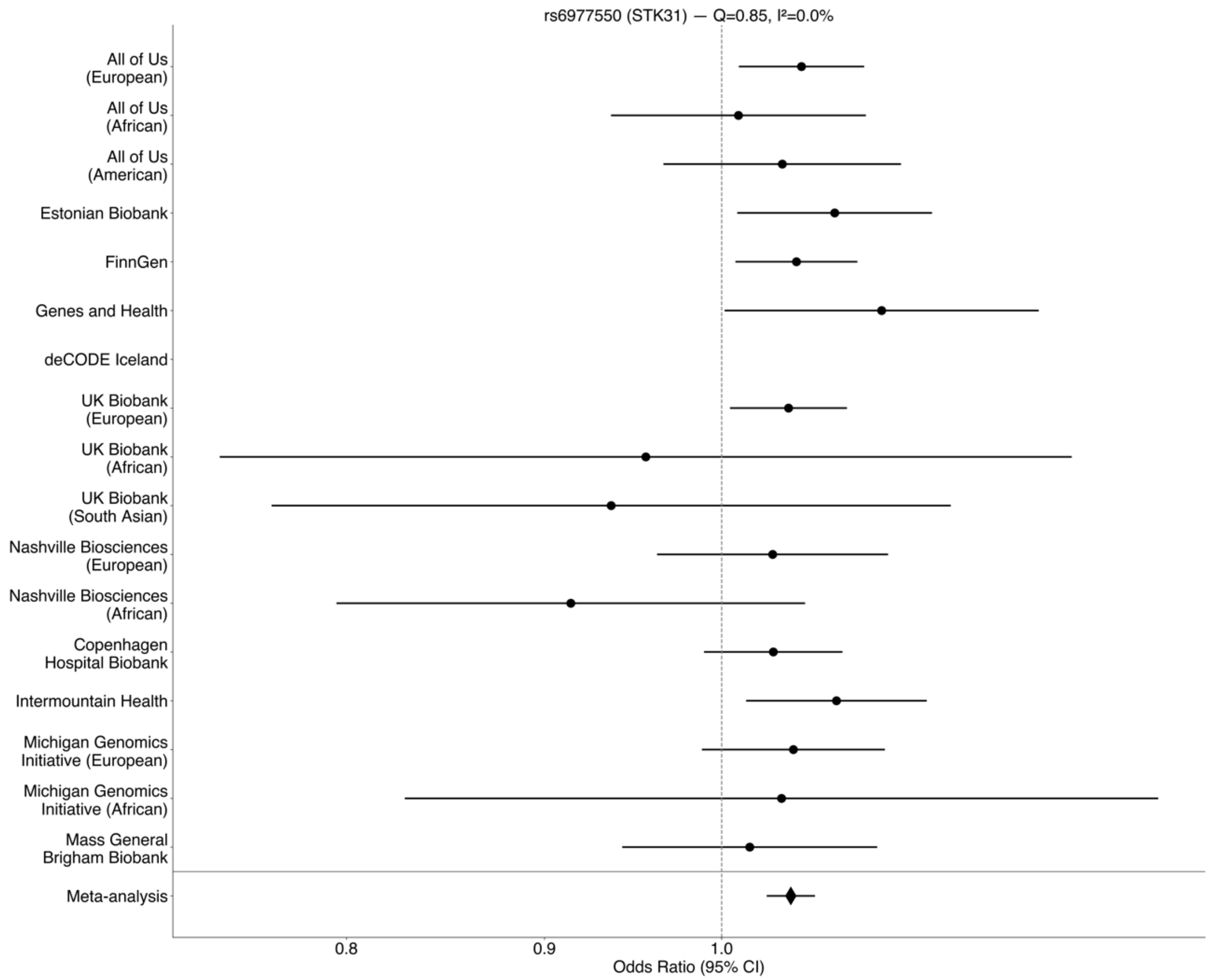

rs6977550 (STK31) — Q=0.85, I²=0.0%
All of Us (European)
All of Us (African)
All of Us (American)
Estonian Biobank
FinnGen
Genes and Health
deCODE Iceland
UK Biobank (European)
UK Biobank (African)
UK Biobank (South Asian)
Nashville Biosciences (European)
Nashville Biosciences (African)
Copenhagen Hospital Biobank
Intermountain Health
Michigan Genomics Initiative (European)
Michigan Genomics Initiative (African)
Mass General Brigham Biobank
Meta-analysis
0.8
0.9
1.0
Odds Ratio (95% CI)

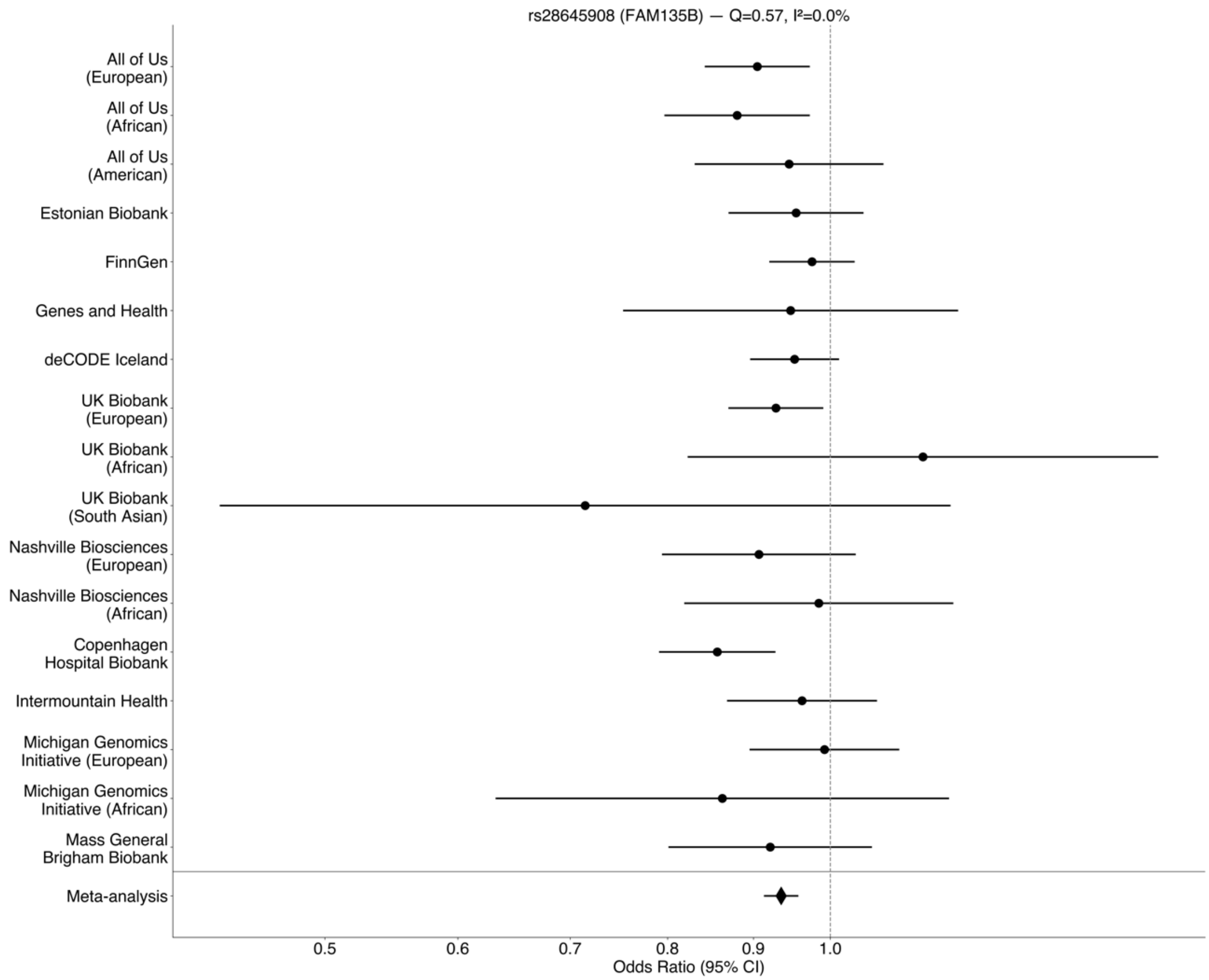

rs28645908 (FAM135B) — Q=0.57, I²=0.0%
All of Us (European)
All of Us (African)
All of Us (American)
Estonian Biobank
FinnGen
Genes and Health
deCODE Iceland
UK Biobank (European)
UK Biobank (African)
UK Biobank (South Asian)
Nashville Biosciences (European)
Nashville Biosciences (African)
Copenhagen Hospital Biobank
Intermountain Health
Michigan Genomics Initiative (European)
Michigan Genomics Initiative (African)
Mass General Brigham Biobank
Meta-analysis
0.5
0.6
0.7
0.8
0.9
1.0
Odds Ratio (95% CI)

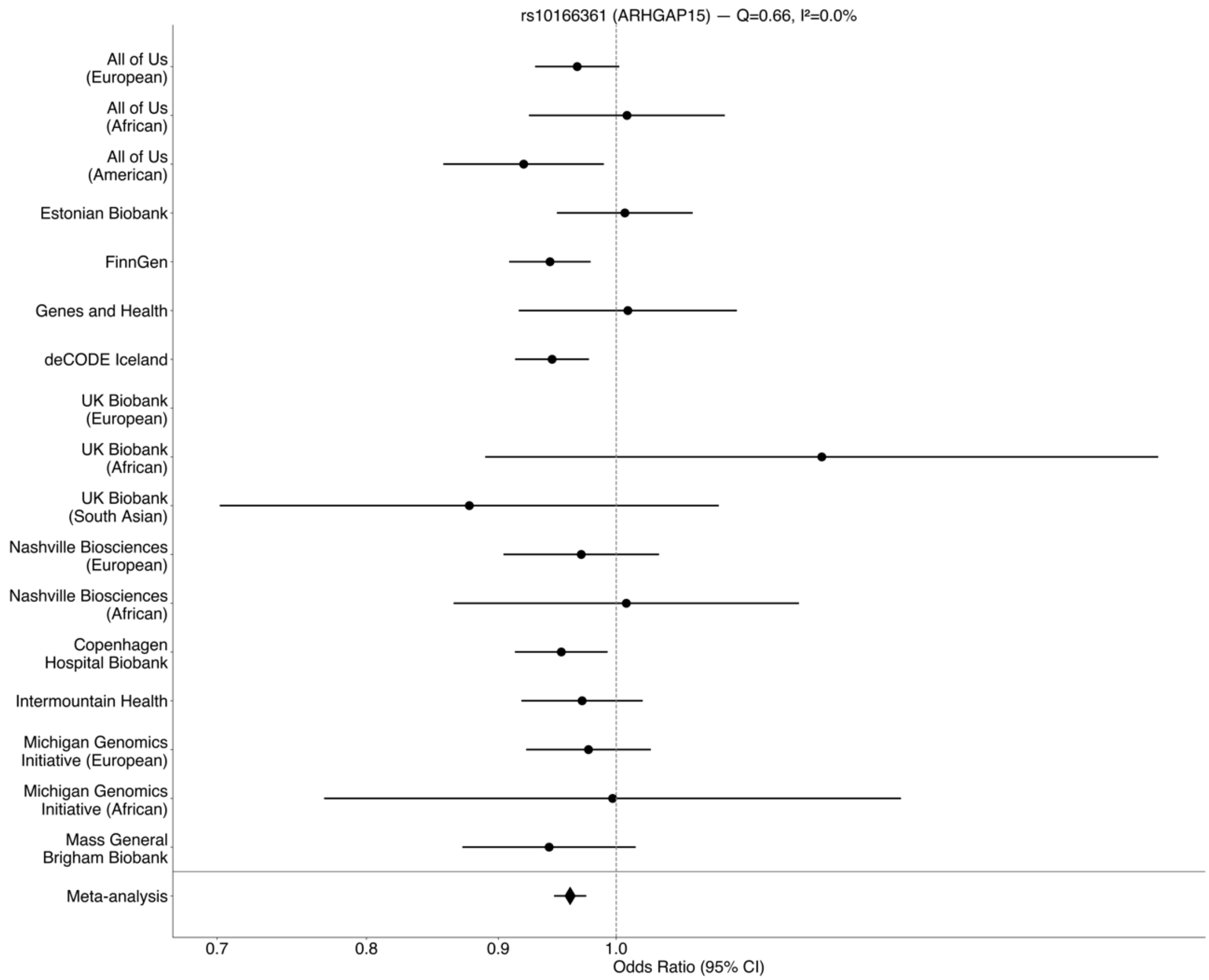
rs10166361 (ARHGAP15) — Q=0.66, I²=0.0%
All of Us (European)
All of Us (African)
All of Us (American)
Estonian Biobank
FinnGen
Genes and Health
deCODE Iceland
UK Biobank (European)
UK Biobank (African)
UK Biobank (South Asian)
Nashville Biosciences (European)
Nashville Biosciences (African)
Copenhagen Hospital Biobank
Intermountain Health
Michigan Genomics Initiative (European)
Michigan Genomics Initiative (African)
Mass General Brigham Biobank
Meta-analysis
0.7
0.8
0.9
1.0
Odds Ratio (95% CI)

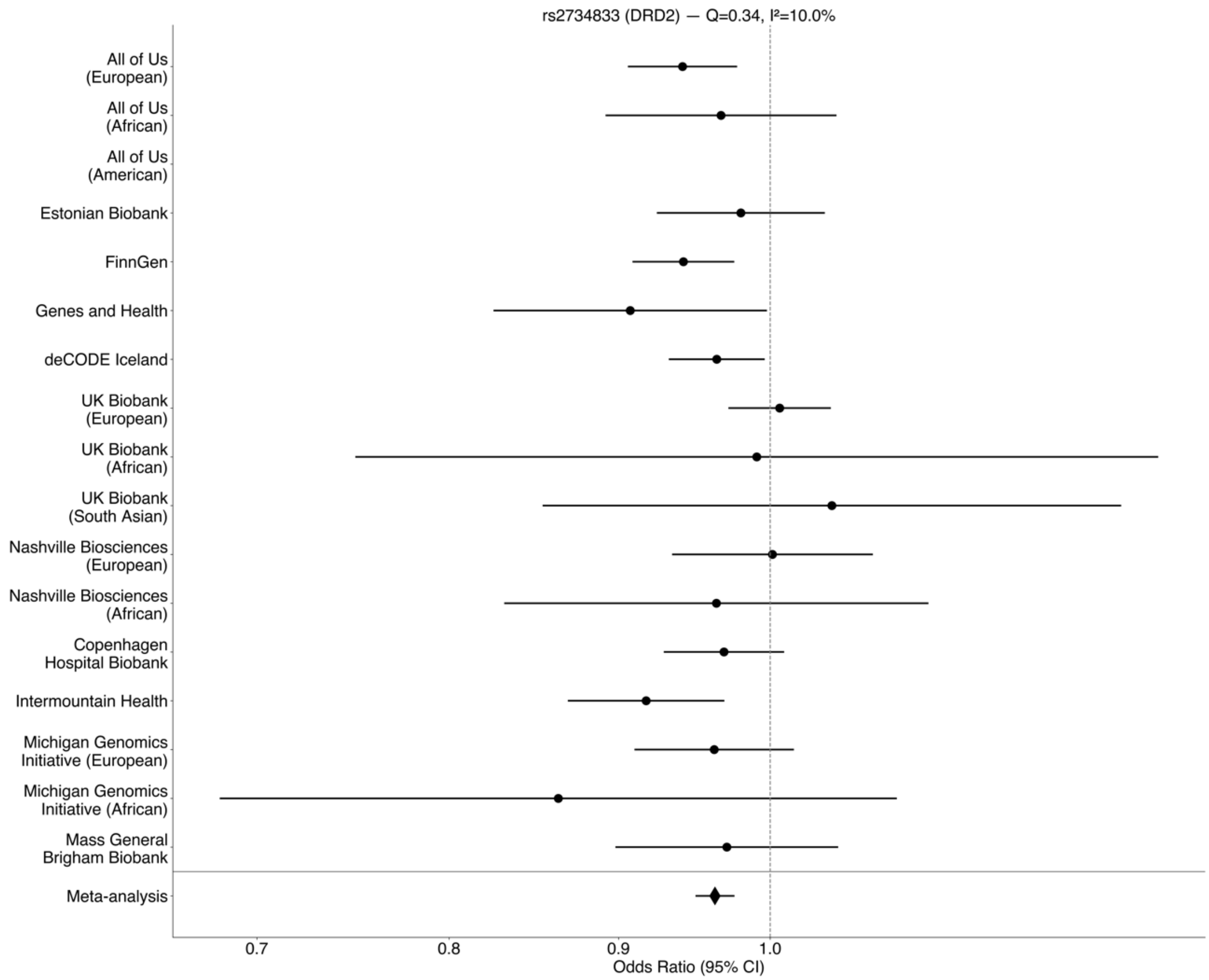
rs2734833 (DRD2) — Q=0.34, I²=10.0%
All of Us (European)
All of Us (African)
All of Us (American)
Estonian Biobank
FinnGen
Genes and Health
deCODE Iceland
UK Biobank (European)
UK Biobank (African)
UK Biobank (South Asian)
Nashville Biosciences (European)
Nashville Biosciences (African)
Copenhagen Hospital Biobank
Intermountain Health
Michigan Genomics Initiative (European)
Michigan Genomics Initiative (African)
Mass General Brigham Biobank
Meta-analysis
0.7
0.8
0.9
1.0
Odds Ratio (95% CI)

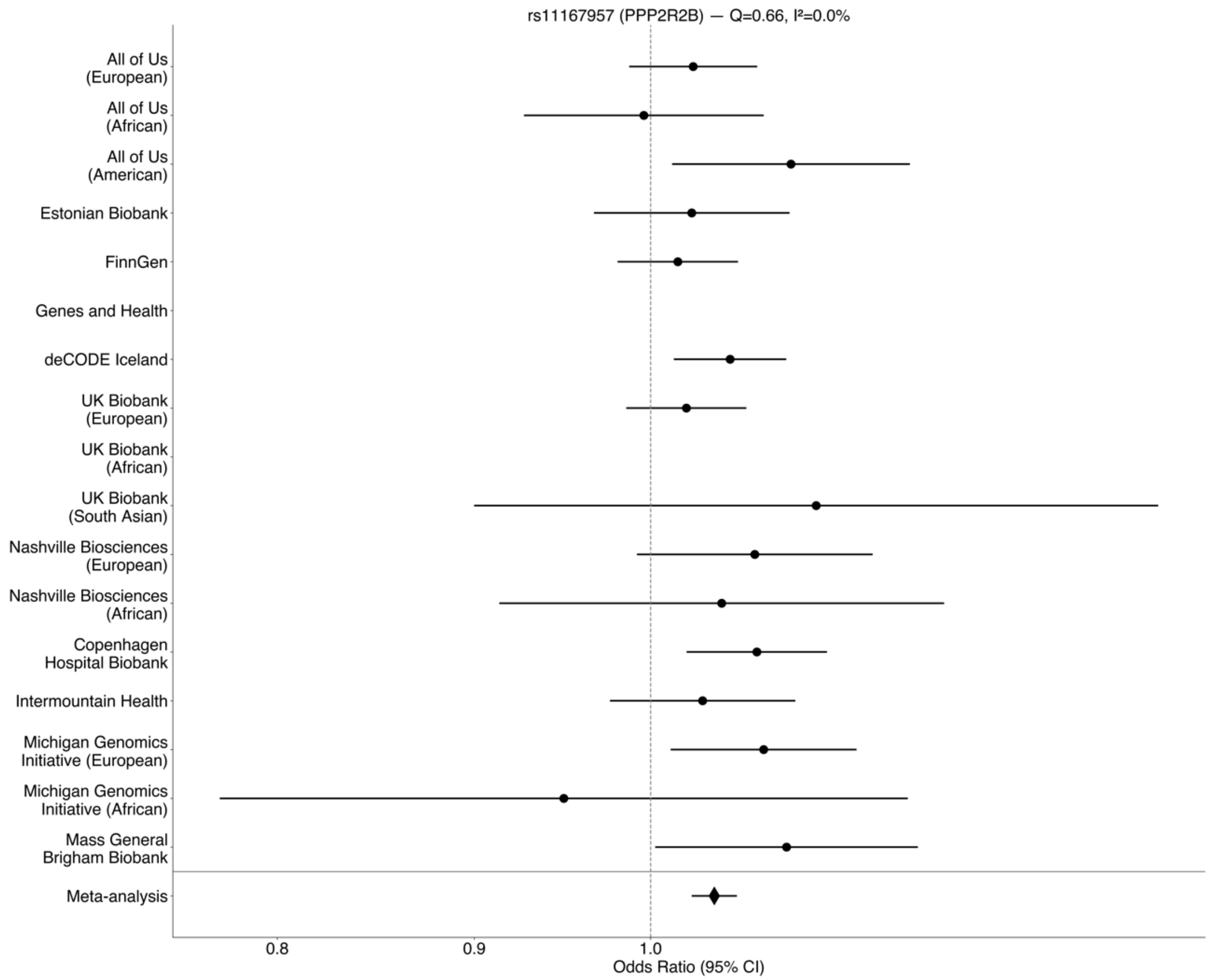

rs11167957 (PPP2R2B) — Q=0.66, I²=0.0%
All of Us (European)
All of Us (African)
All of Us (American)
Estonian Biobank
FinnGen
Genes and Health
deCODE Iceland
UK Biobank (European)
UK Biobank (African)
UK Biobank (South Asian)
Nashville Biosciences (European)
Nashville Biosciences (African)
Copenhagen Hospital Biobank
Intermountain Health
Michigan Genomics Initiative (European)
Michigan Genomics Initiative (African)
Mass General Brigham Biobank
Meta-analysis
0.8
0.9
1.0
Odds Ratio (95% CI)

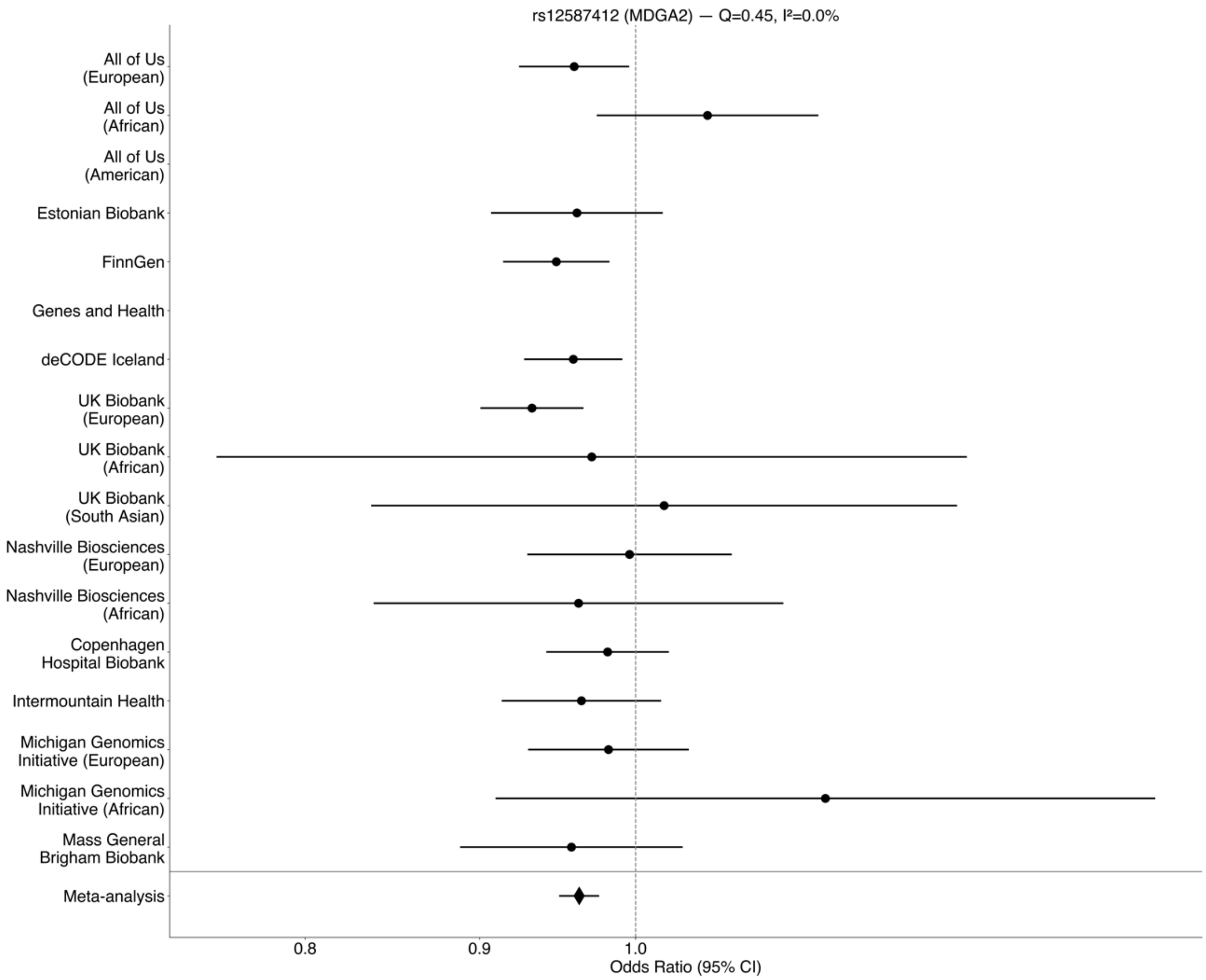
rs12587412 (MDGA2) — Q=0.45, I²=0.0%
All of Us (European)
All of Us (African)
All of Us (American)
Estonian Biobank
FinnGen
Genes and Health
deCODE Iceland
UK Biobank (European)
UK Biobank (African)
UK Biobank (South Asian)
Nashville Biosciences (European)
Nashville Biosciences (African)
Copenhagen Hospital Biobank
Intermountain Health
Michigan Genomics Initiative (European)
Michigan Genomics Initiative (African)
Mass General Brigham Biobank
Meta-analysis
0.8
0.9
1.0
Odds Ratio (95% CI)

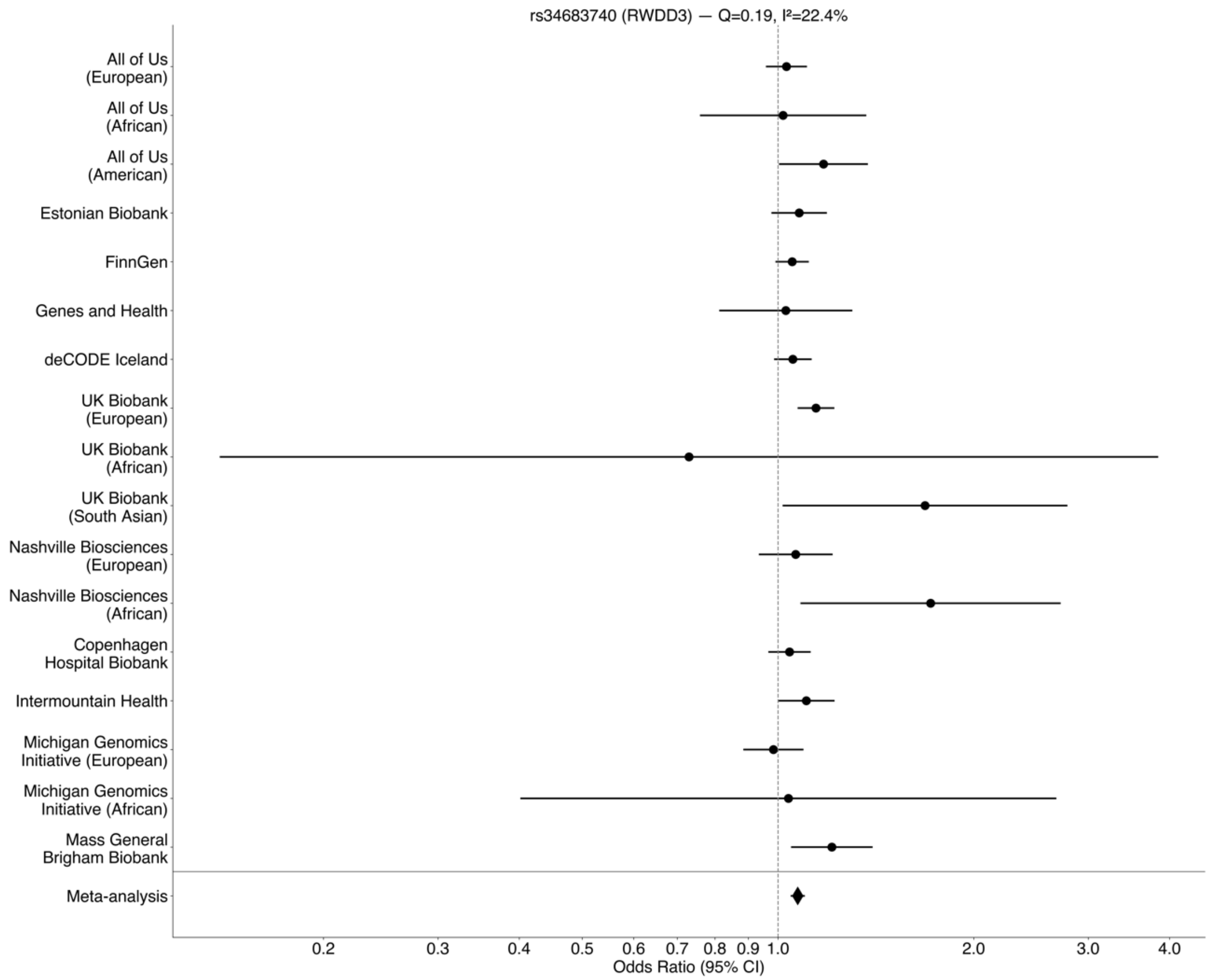
rs34683740 (RWDD3) — Q=0.19, I²=22.4%
All of Us (European)
All of Us (African)
All of Us (American)
Estonian Biobank
FinnGen
Genes and Health
deCODE Iceland
UK Biobank (European)
UK Biobank (African)
UK Biobank (South Asian)
Nashville Biosciences (European)
Nashville Biosciences (African)
Copenhagen Hospital Biobank
Intermountain Health
Michigan Genomics Initiative (European)
Michigan Genomics Initiative (African)
Mass General Brigham Biobank
Meta-analysis
0.2 0.3 0.4 0.5 0.6 0.7 0.8 0.9 1.0 2.0 3.0 4.0
Odds Ratio (95% CI)

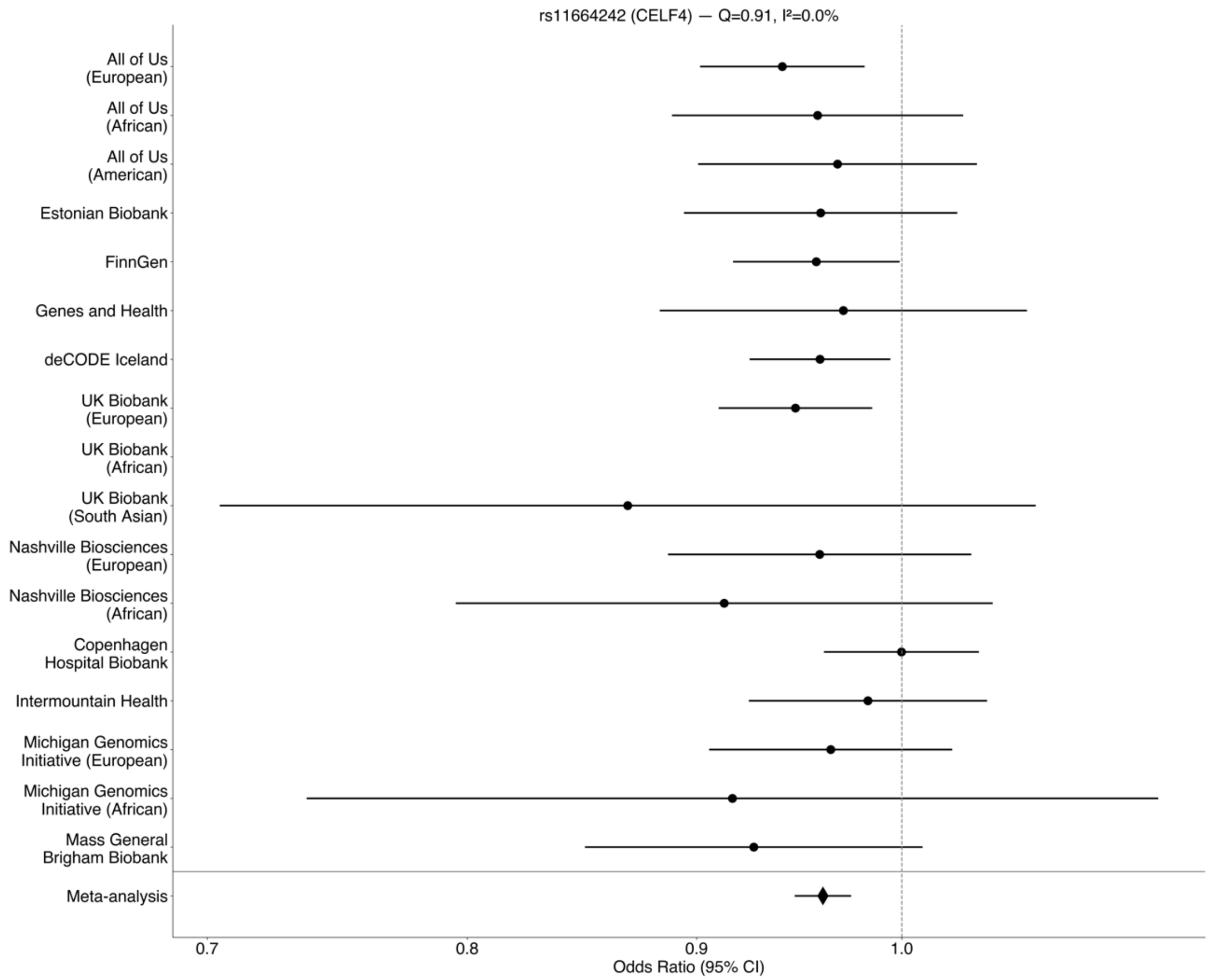

rs11664242 (CELF4) — Q=0.91, I²=0.0%
All of Us (European)
All of Us (African)
All of Us (American)
Estonian Biobank
FinnGen
Genes and Health
deCODE Iceland
UK Biobank (European)
UK Biobank (African)
UK Biobank (South Asian)
Nashville Biosciences (European)
Nashville Biosciences (African)
Copenhagen Hospital Biobank
Intermountain Health
Michigan Genomics Initiative (European)
Michigan Genomics Initiative (African)
Mass General Brigham Biobank
Meta-analysis
0.7
0.8
0.9
1.0
Odds Ratio (95% CI)

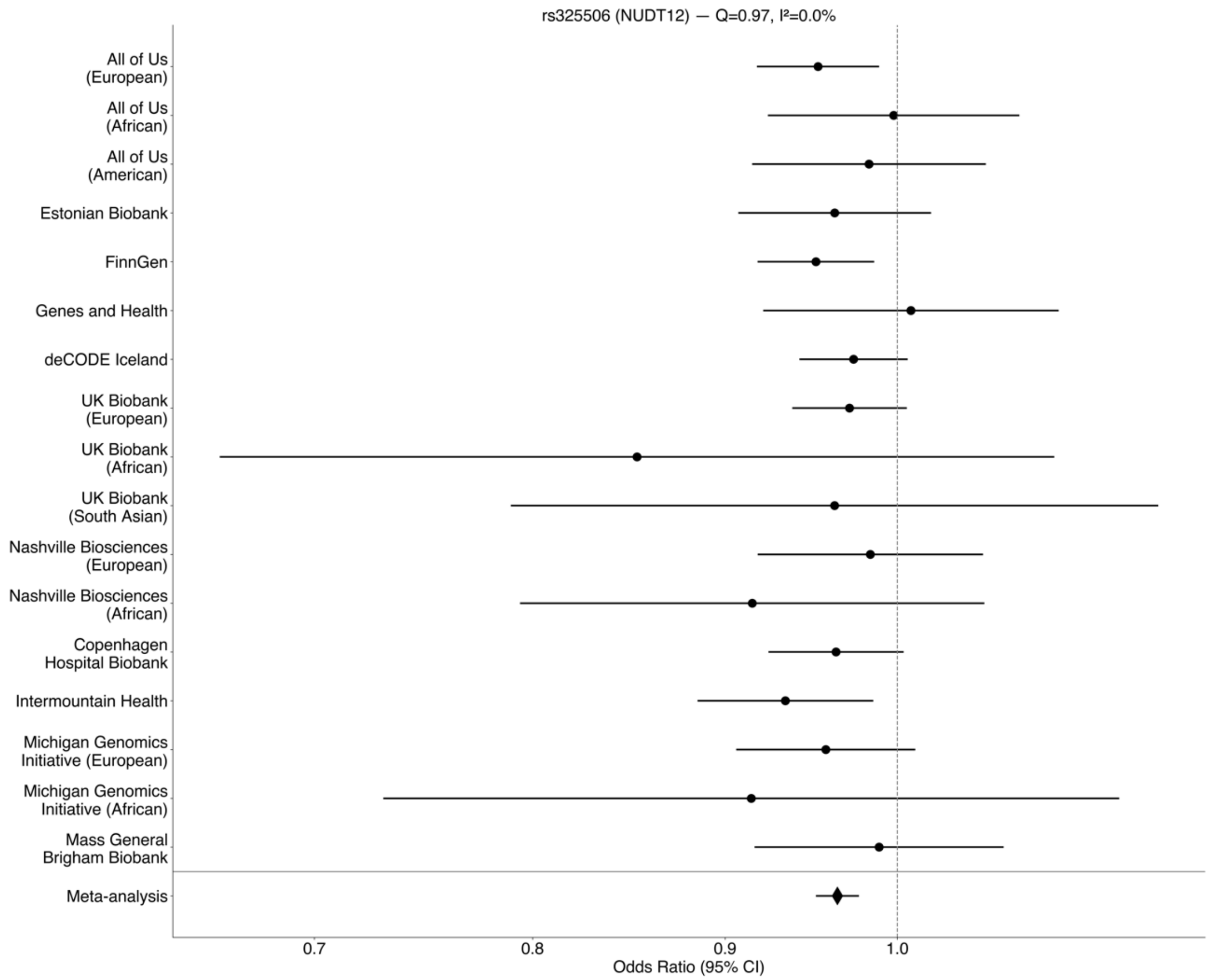

Figure S1: Forest plots of lead variants from the multi-ancestry meta-analysis. Effect estimates are plotted as odds ratios with 95% confidence intervals. “Copenhagen Hospital Biobank” is short for “Copenhagen Hospital Biobank and Danish Blood Donor Study”.

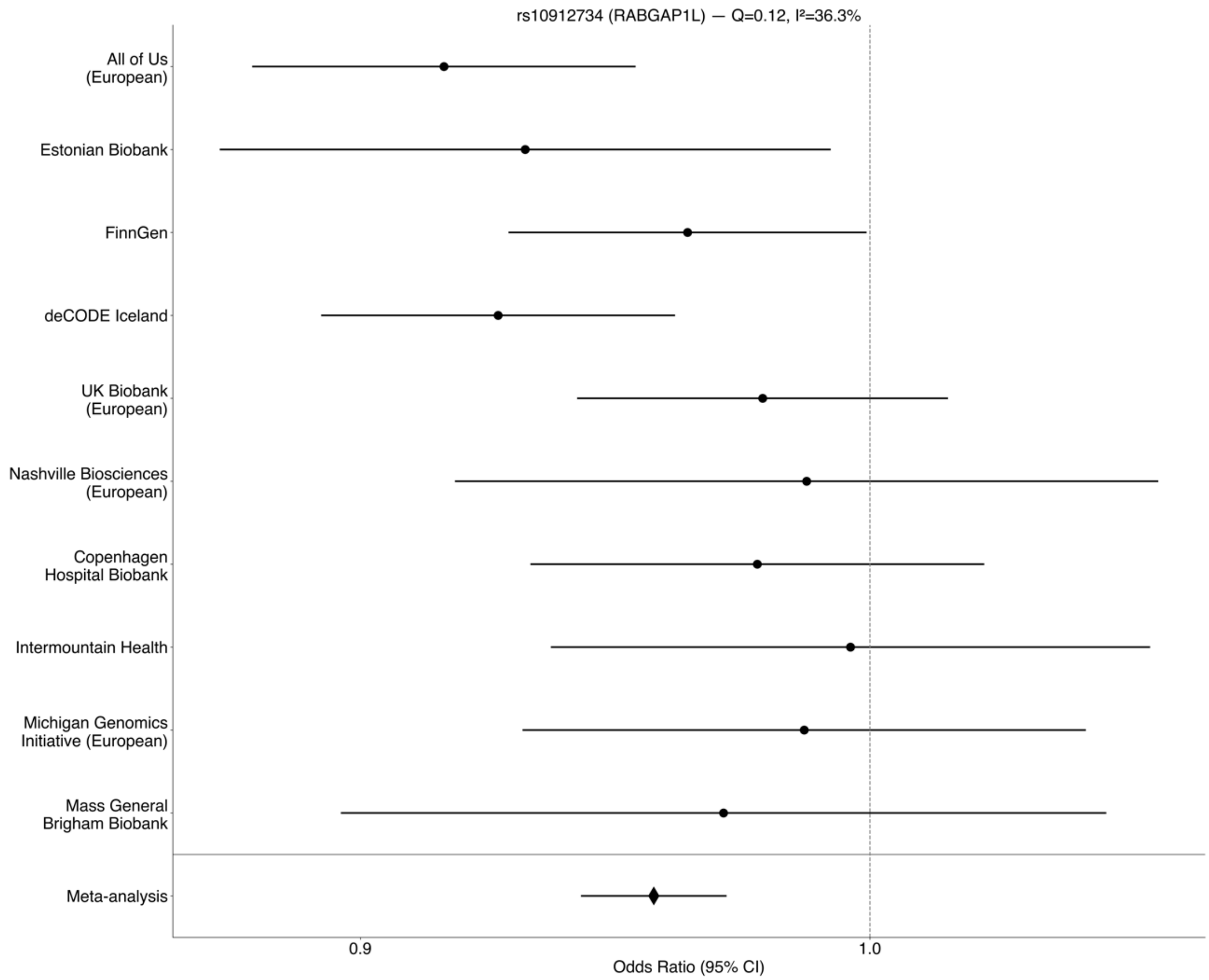

rs10912734 (RABGAP1L) — Q=0.12, I²=36.3%
All of Us (European)
Estonian Biobank
FinnGen
deCODE Iceland
UK Biobank (European)
Nashville Biosciences (European)
Copenhagen Hospital Biobank
Intermountain Health
Michigan Genomics Initiative (European)
Mass General Brigham Biobank
Meta-analysis
0.9
1.0
Odds Ratio (95% CI)

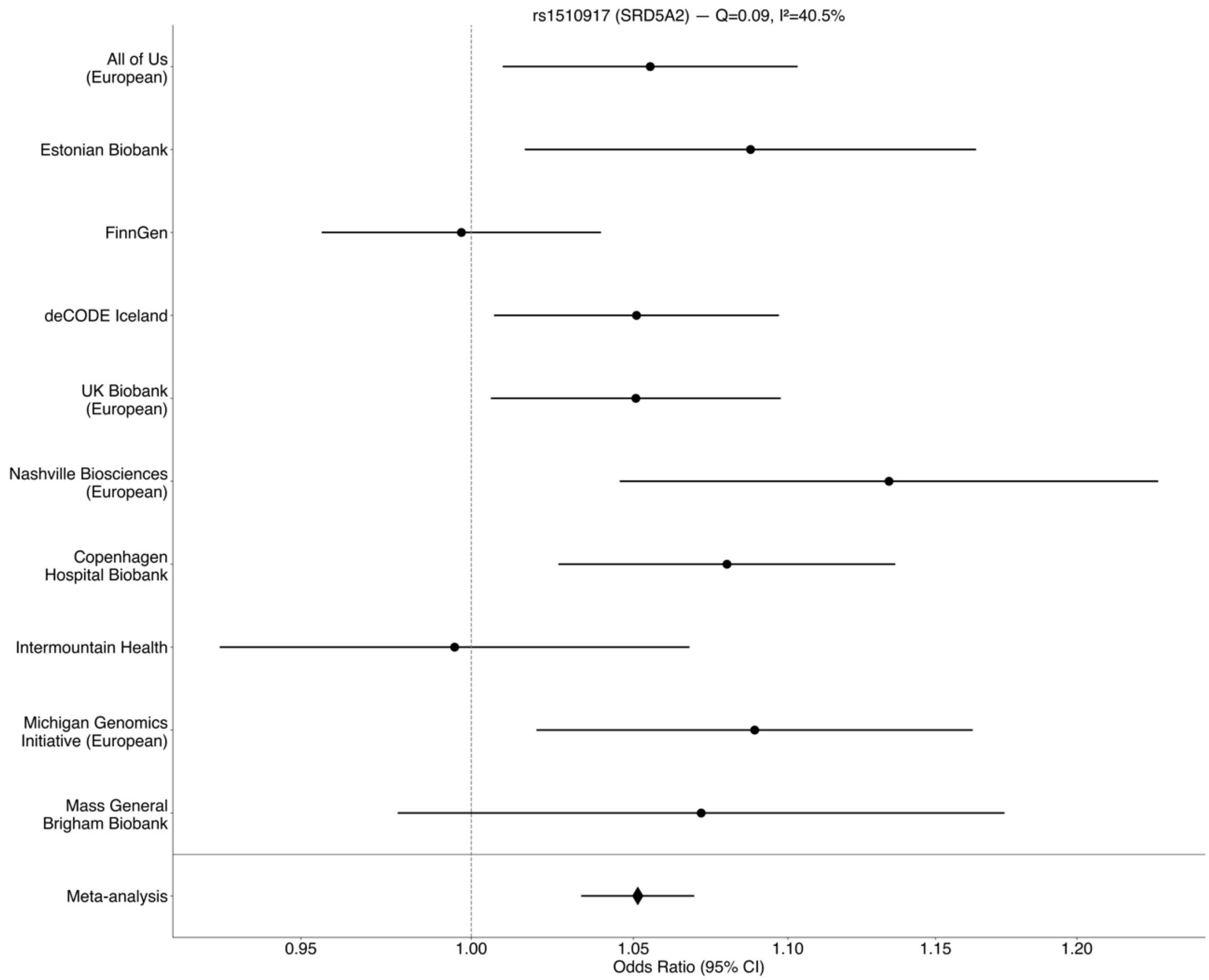
rs1510917 (SRD5A2) — Q=0.09, I²=40.5%
All of Us (European)
Estonian Biobank
FinnGen
deCODE Iceland
UK Biobank (European)
Nashville Biosciences (European)
Copenhagen Hospital Biobank
Intermountain Health
Michigan Genomics Initiative (European)
Mass General Brigham Biobank
Meta-analysis
0.95
1.00
1.05
1.10
1.15
1.20
Odds Ratio (95% CI)

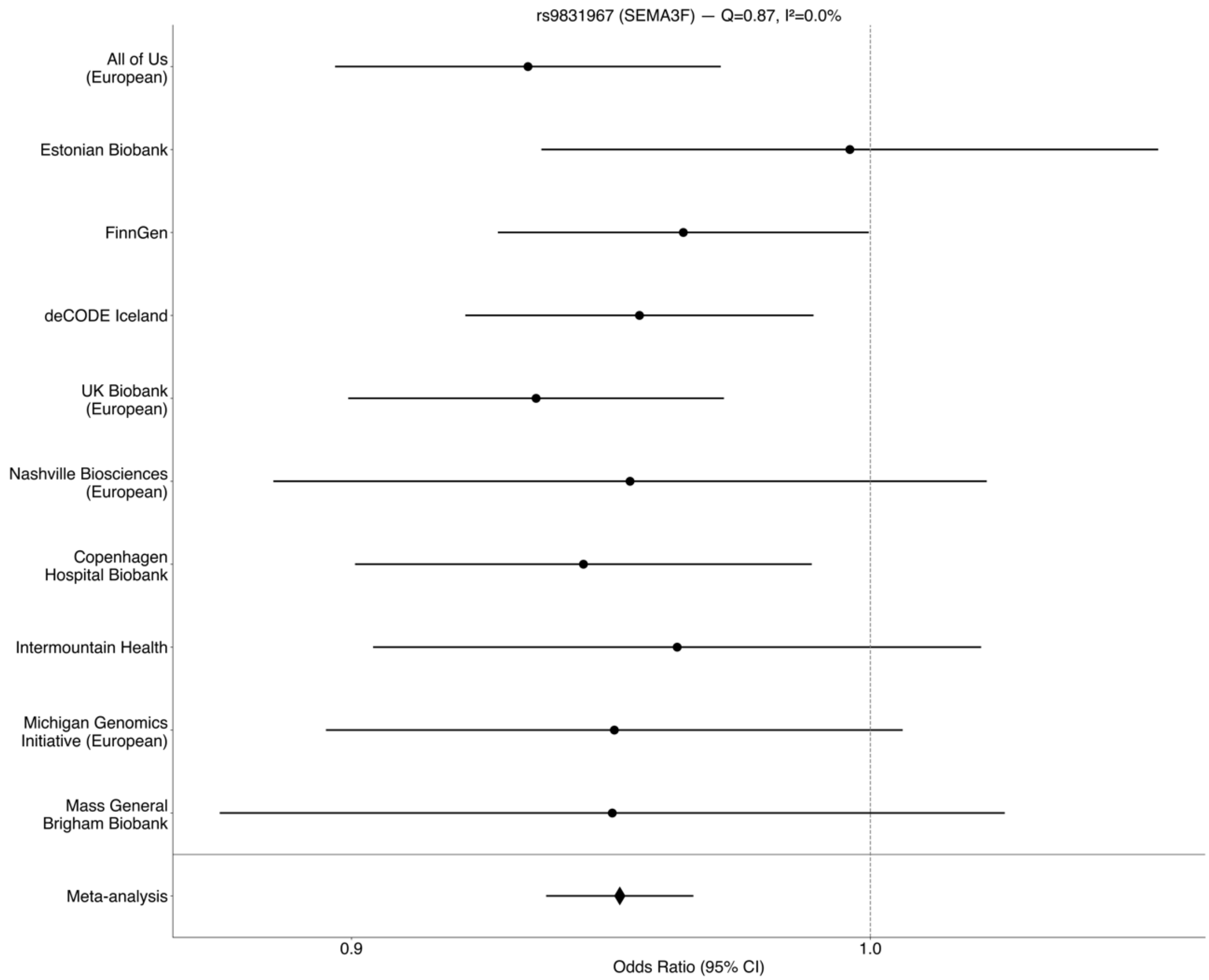
rs9831967 (SEMA3F) — Q=0.87, I²=0.0%
All of Us (European)
Estonian Biobank
FinnGen
deCODE Iceland
UK Biobank (European)
Nashville Biosciences (European)
Copenhagen Hospital Biobank
Intermountain Health
Michigan Genomics Initiative (European)
Mass General Brigham Biobank
Meta-analysis
0.9
1.0
Odds Ratio (95% CI)

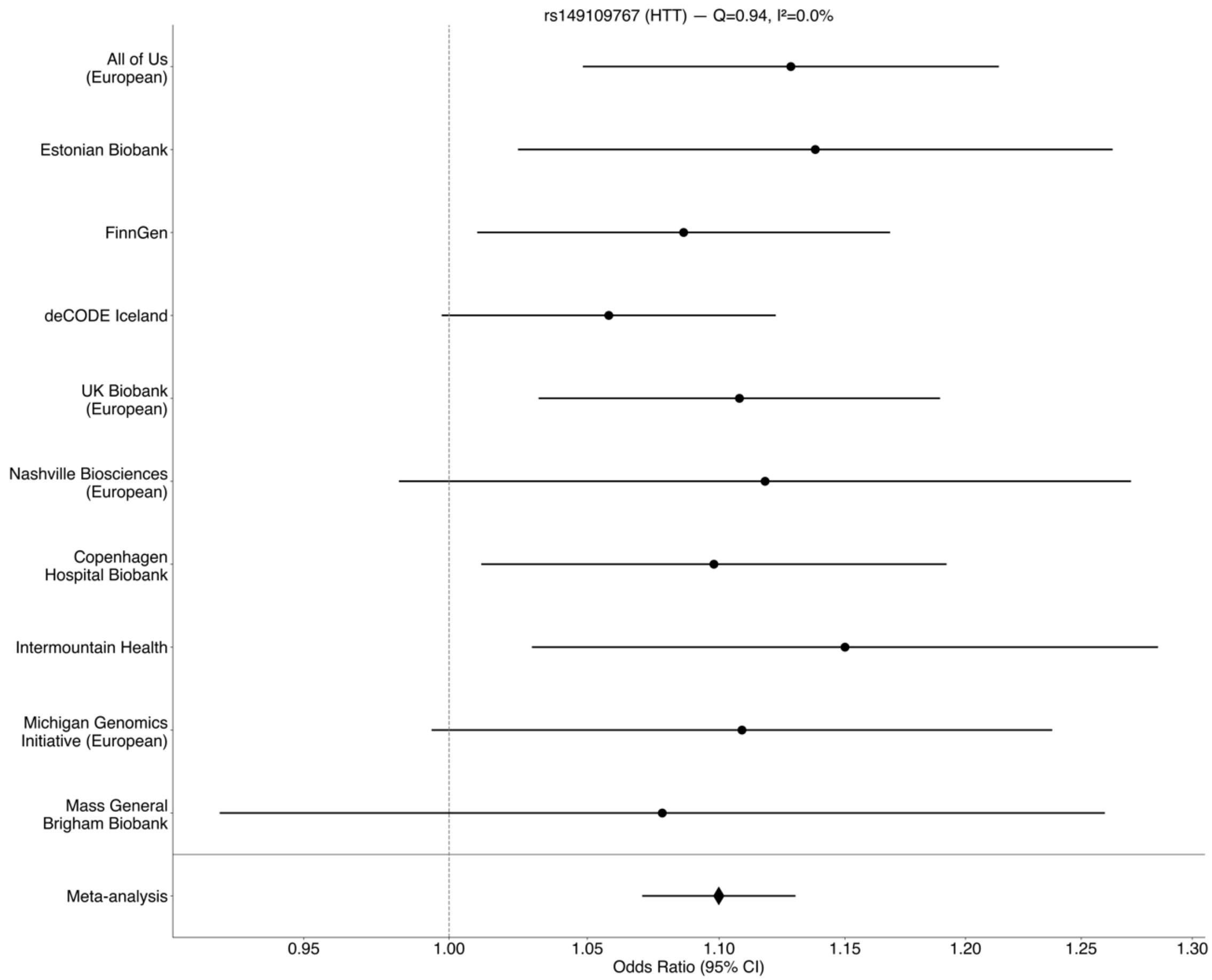
rs149109767 (HTT) — Q=0.94, I²=0.0%
All of Us
(European)
Estonian Biobank
FinnGen
deCODE Iceland
UK Biobank
(European)
Nashville Biosciences
(European)
Copenhagen
Hospital Biobank
Intermountain Health
Michigan Genomics
Initiative (European)
Mass General
Brigham Biobank
Meta-analysis
0.95
1.00
1.05
1.10
1.15
1.20
1.25
1.30
Odds Ratio (95% CI)

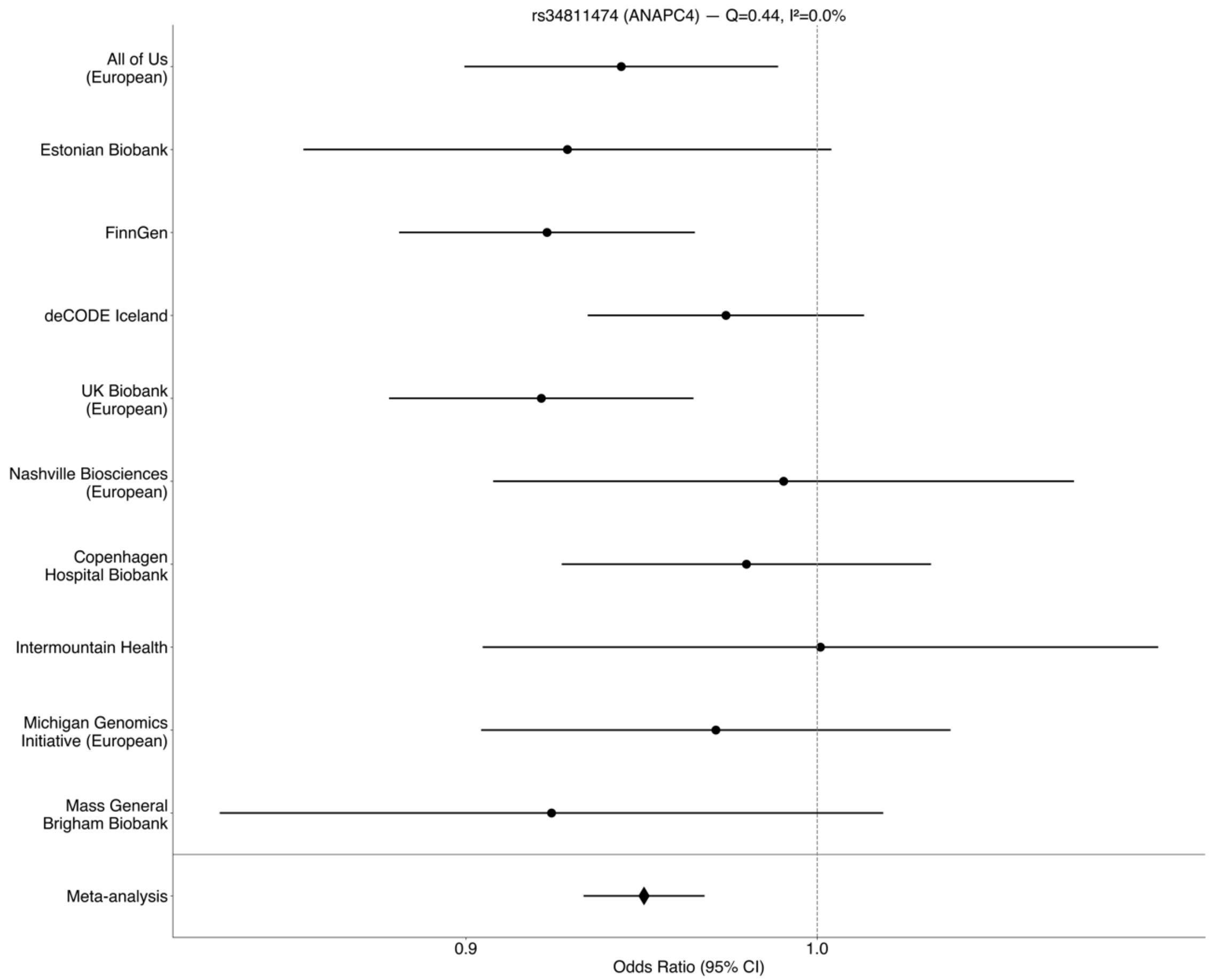
rs34811474 (ANAPC4) — Q=0.44, I²=0.0%
All of Us (European)
Estonian Biobank
FinnGen
deCODE Iceland
UK Biobank (European)
Nashville Biosciences (European)
Copenhagen Hospital Biobank
Intermountain Health
Michigan Genomics Initiative (European)
Mass General Brigham Biobank
Meta-analysis
0.9
1.0
Odds Ratio (95% CI)

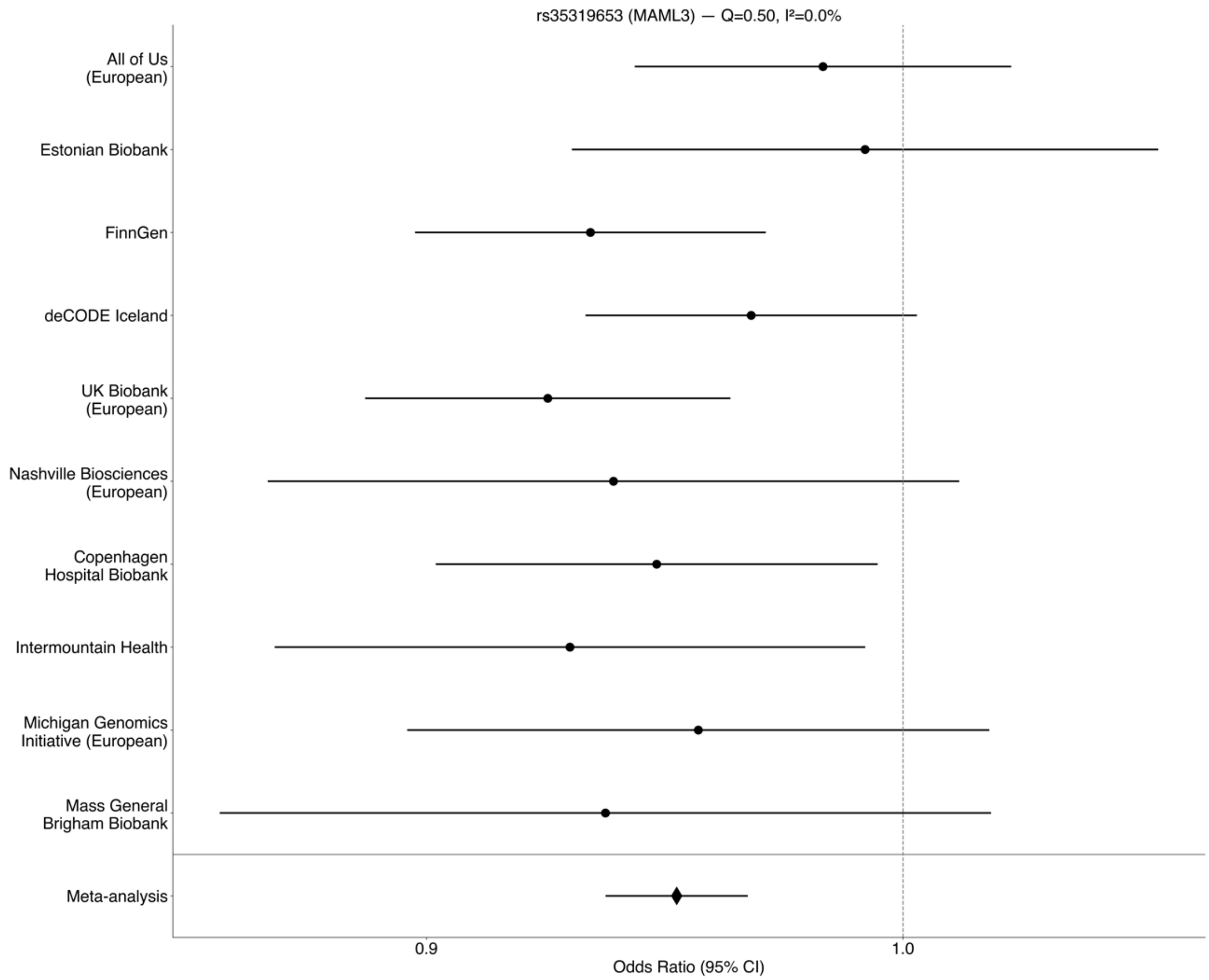
rs35319653 (MAML3) — Q=0.50, I²=0.0%
All of Us (European)
Estonian Biobank
FinnGen
deCODE Iceland
UK Biobank (European)
Nashville Biosciences (European)
Copenhagen Hospital Biobank
Intermountain Health
Michigan Genomics Initiative (European)
Mass General Brigham Biobank
Meta-analysis
0.9
1.0
Odds Ratio (95% CI)

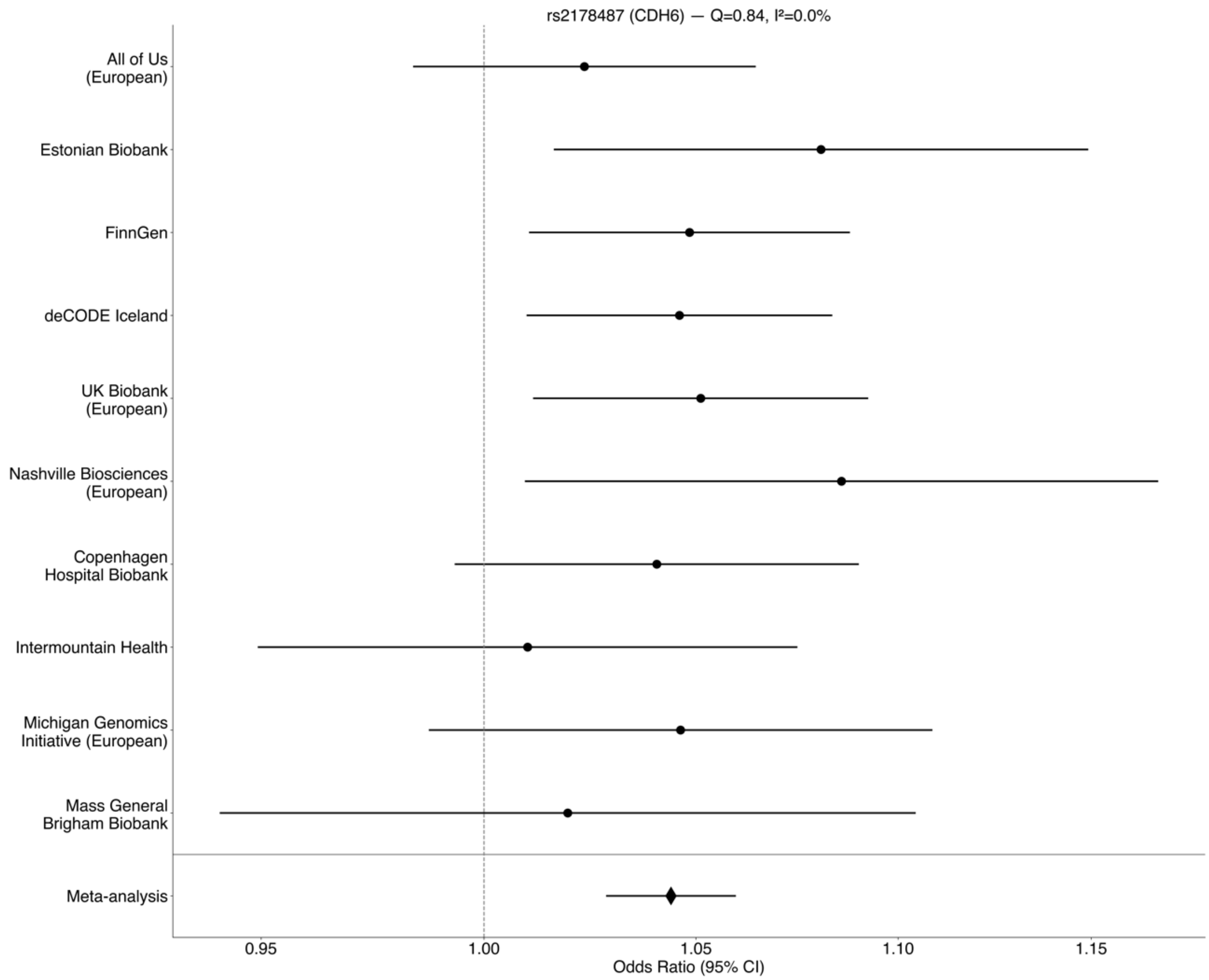
rs2178487 (CDH6) — Q=0.84, I²=0.0%
All of Us (European)
Estonian Biobank
FinnGen
deCODE Iceland
UK Biobank (European)
Nashville Biosciences (European)
Copenhagen Hospital Biobank
Intermountain Health
Michigan Genomics Initiative (European)
Mass General Brigham Biobank
Meta-analysis
0.95
1.00
1.05
1.10
1.15
Odds Ratio (95% CI)

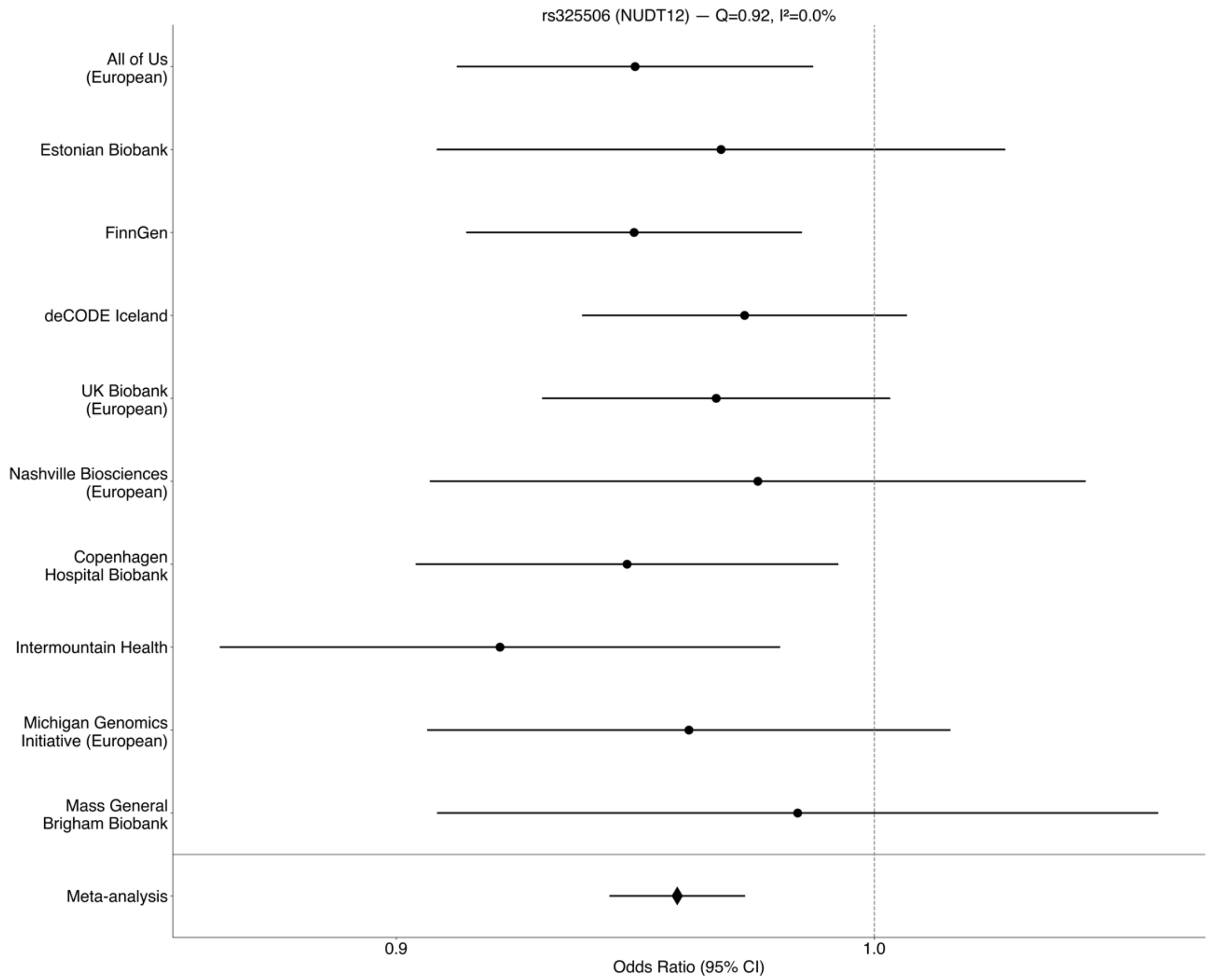
rs325506 (NUDT12) — Q=0.92, I²=0.0%
All of Us (European)
Estonian Biobank
FinnGen
deCODE Iceland
UK Biobank (European)
Nashville Biosciences (European)
Copenhagen Hospital Biobank
Intermountain Health
Michigan Genomics Initiative (European)
Mass General Brigham Biobank
Meta-analysis
0.9
1.0
Odds Ratio (95% CI)

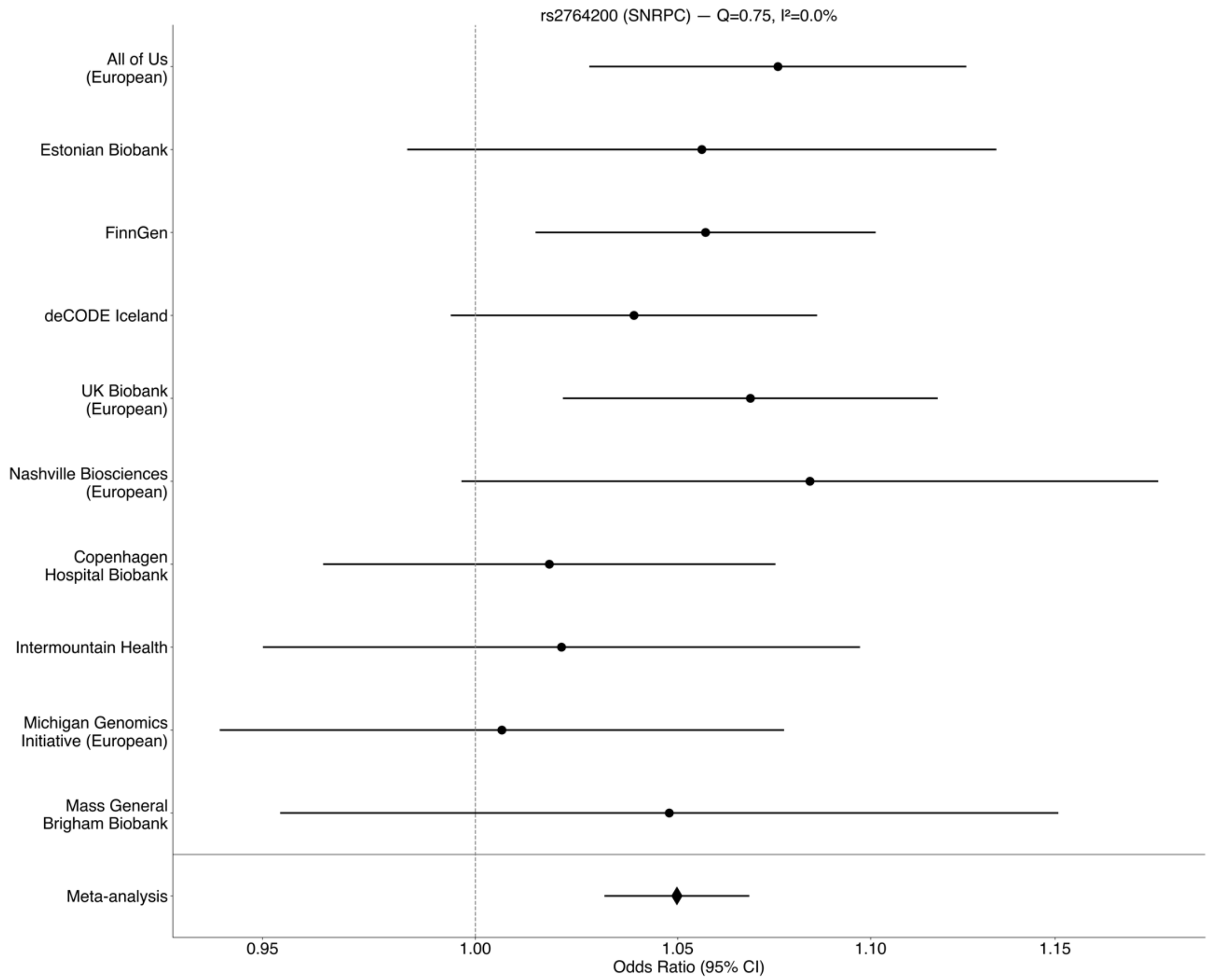
rs2764200 (SNRPC) — Q=0.75, I²=0.0%
All of Us
(European)
Estonian Biobank
FinnGen
deCODE Iceland
UK Biobank
(European)
Nashville Biosciences
(European)
Copenhagen
Hospital Biobank
Intermountain Health
Michigan Genomics
Initiative (European)
Mass General
Brigham Biobank
Meta-analysis
0.95
1.00
1.05
1.10
1.15
Odds Ratio (95% CI)

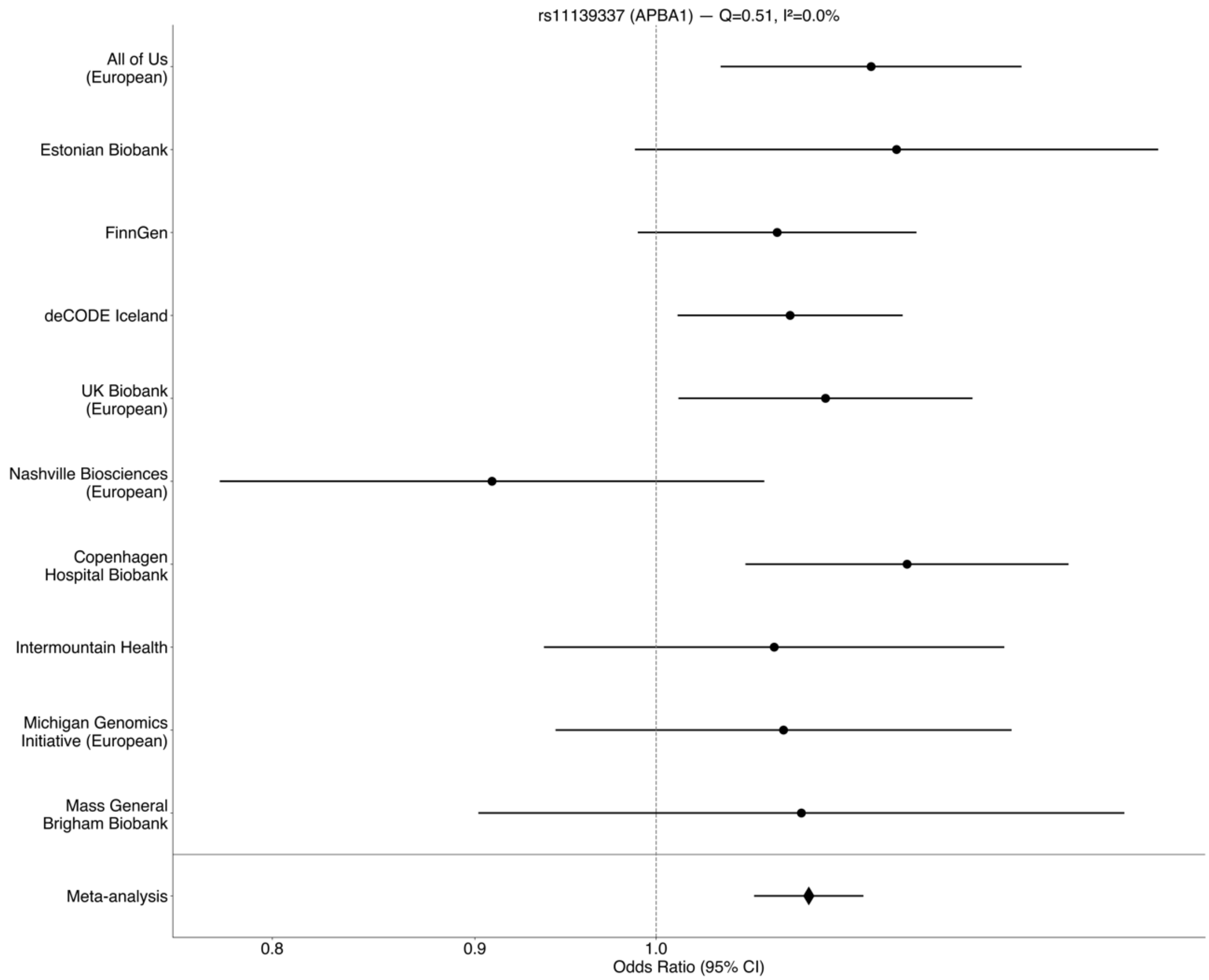
rs11139337 (APBA1) — Q=0.51, I²=0.0%
All of Us
(European)
Estonian Biobank
FinnGen
deCODE Iceland
UK Biobank
(European)
Nashville Biosciences
(European)
Copenhagen
Hospital Biobank
Intermountain Health
Michigan Genomics
Initiative (European)
Mass General
Brigham Biobank
Meta-analysis
0.8
0.9
1.0
Odds Ratio (95% CI)

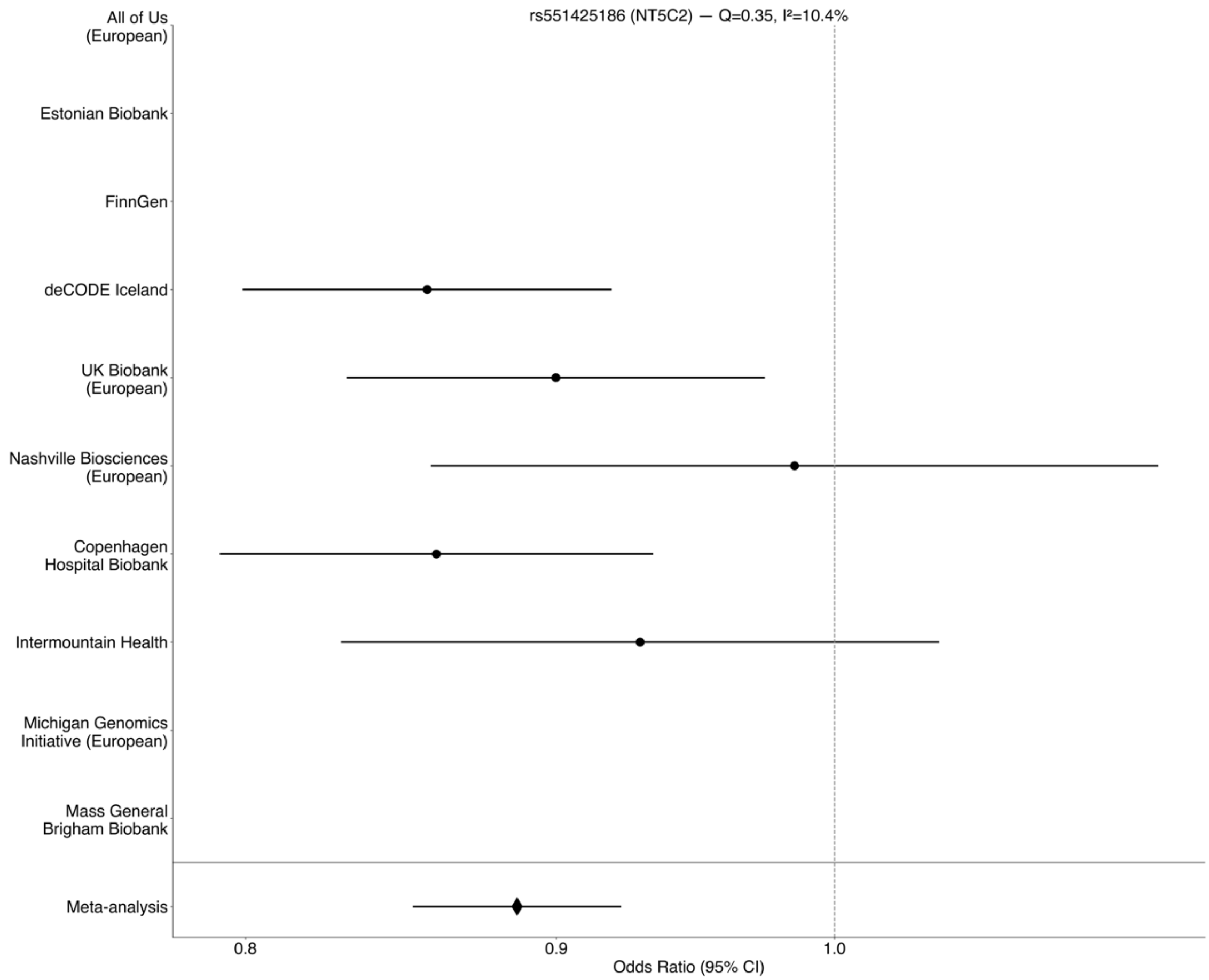
rs551425186 (NT5C2) — Q=0.35, I²=10.4%
All of Us (European)
Estonian Biobank
FinnGen
deCODE Iceland
UK Biobank (European)
Nashville Biosciences (European)
Copenhagen Hospital Biobank
Intermountain Health
Michigan Genomics Initiative (European)
Mass General Brigham Biobank
Meta-analysis
0.8
0.9
1.0
Odds Ratio (95% CI)

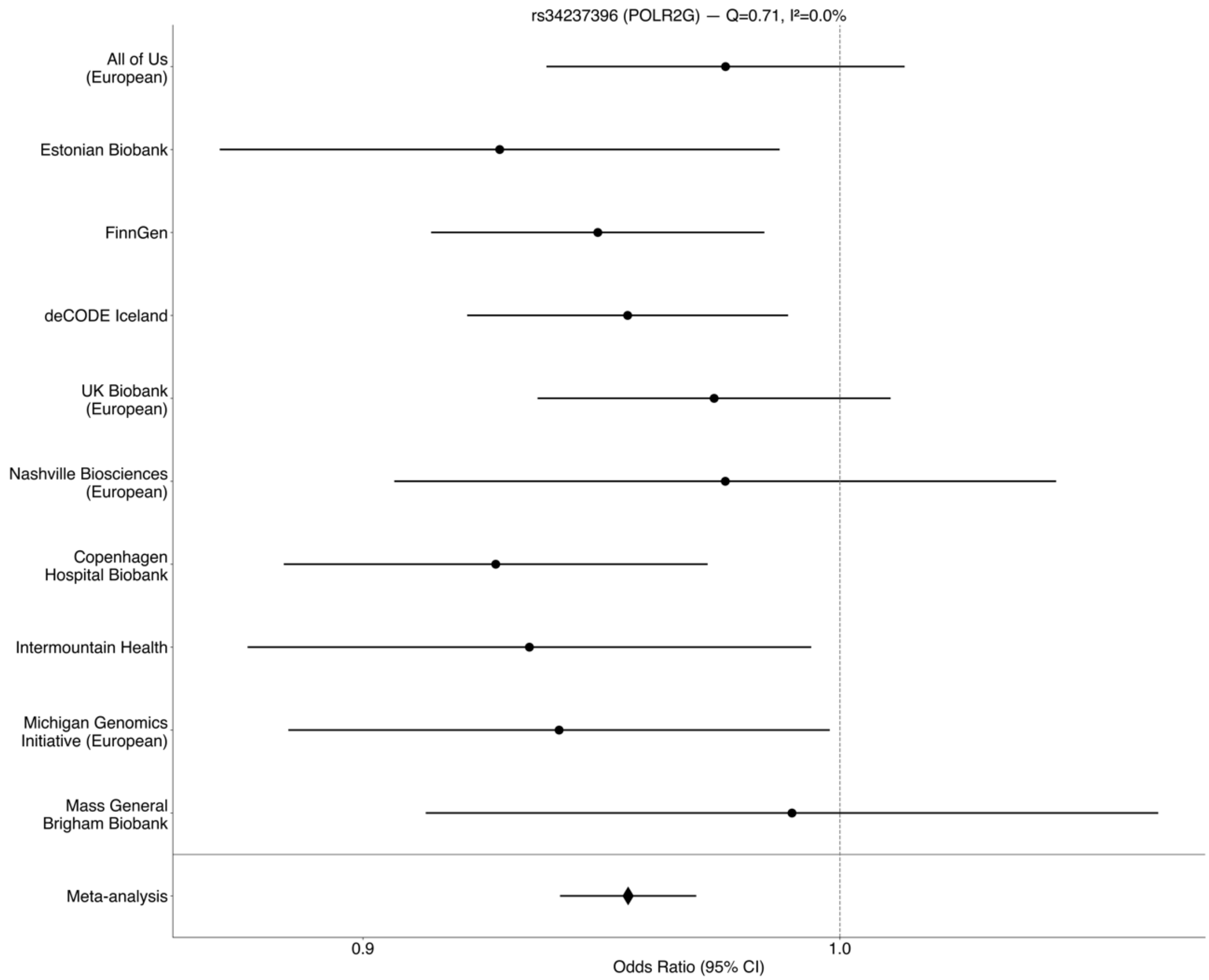
rs34237396 (POLR2G) — Q=0.71, I²=0.0%
All of Us (European)
Estonian Biobank
FinnGen
deCODE Iceland
UK Biobank (European)
Nashville Biosciences (European)
Copenhagen Hospital Biobank
Intermountain Health
Michigan Genomics Initiative (European)
Mass General Brigham Biobank
Meta-analysis
0.9
1.0
Odds Ratio (95% CI)

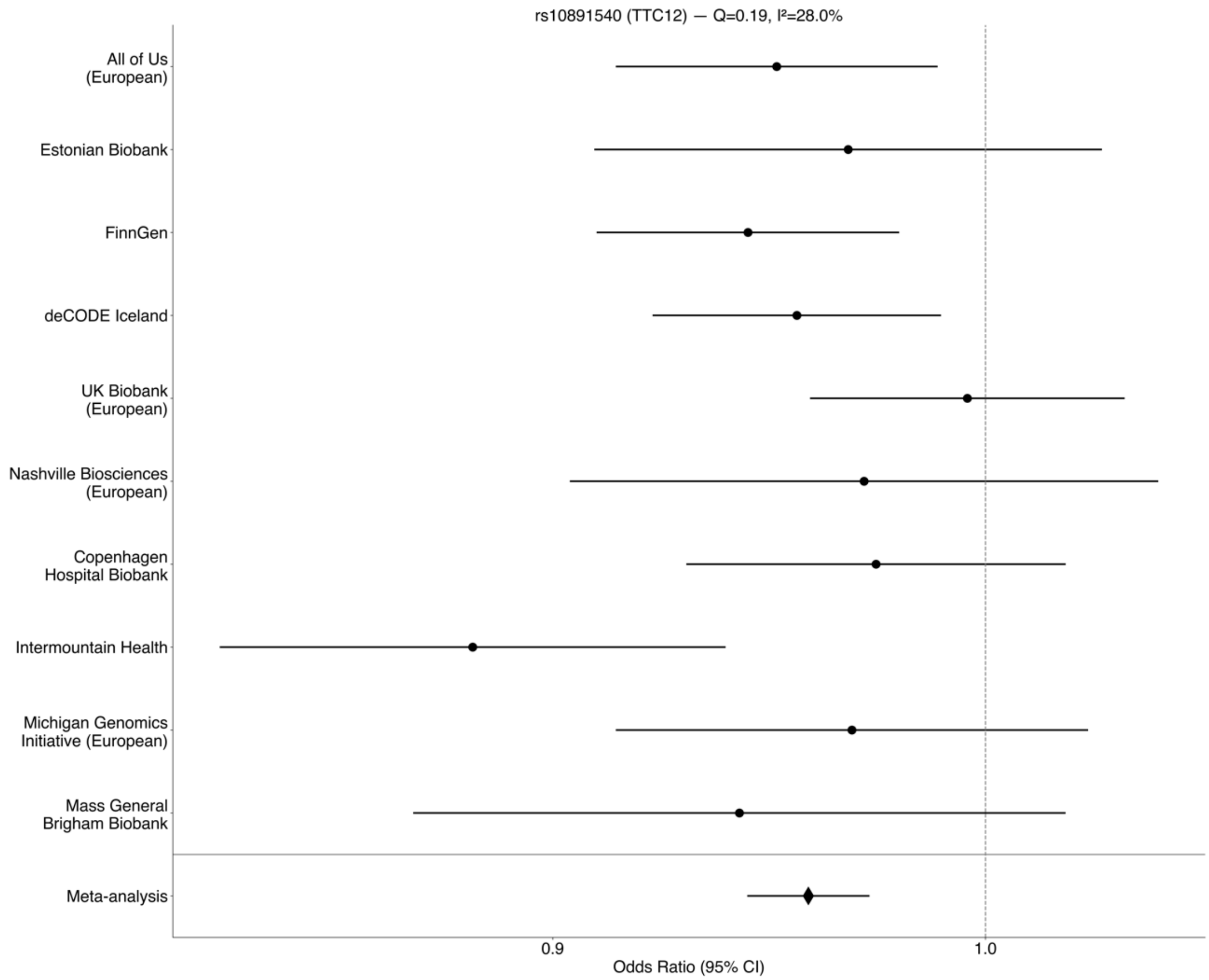
rs10891540 (TTC12) — Q=0.19, I²=28.0%
All of Us (European)
Estonian Biobank
FinnGen
deCODE Iceland
UK Biobank (European)
Nashville Biosciences (European)
Copenhagen Hospital Biobank
Intermountain Health
Michigan Genomics Initiative (European)
Mass General Brigham Biobank
Meta-analysis
0.9
1.0
Odds Ratio (95% CI)

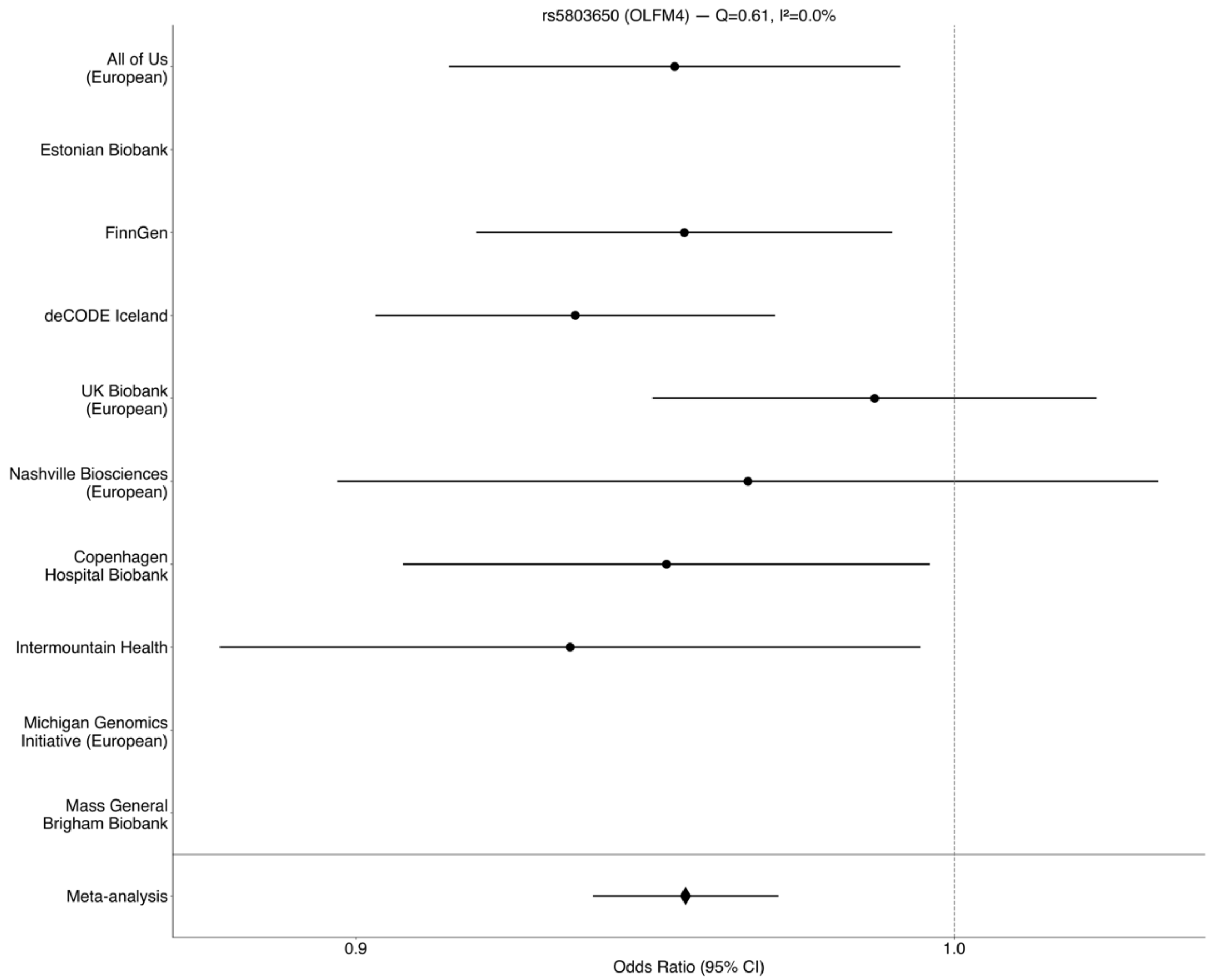

rs5803650 (OLFM4) — Q=0.61, I²=0.0%
All of Us (European)
Estonian Biobank
FinnGen
deCODE Iceland
UK Biobank (European)
Nashville Biosciences (European)
Copenhagen Hospital Biobank
Intermountain Health
Michigan Genomics Initiative (European)
Mass General Brigham Biobank
Meta-analysis
0.9
1.0
Odds Ratio (95% CI)

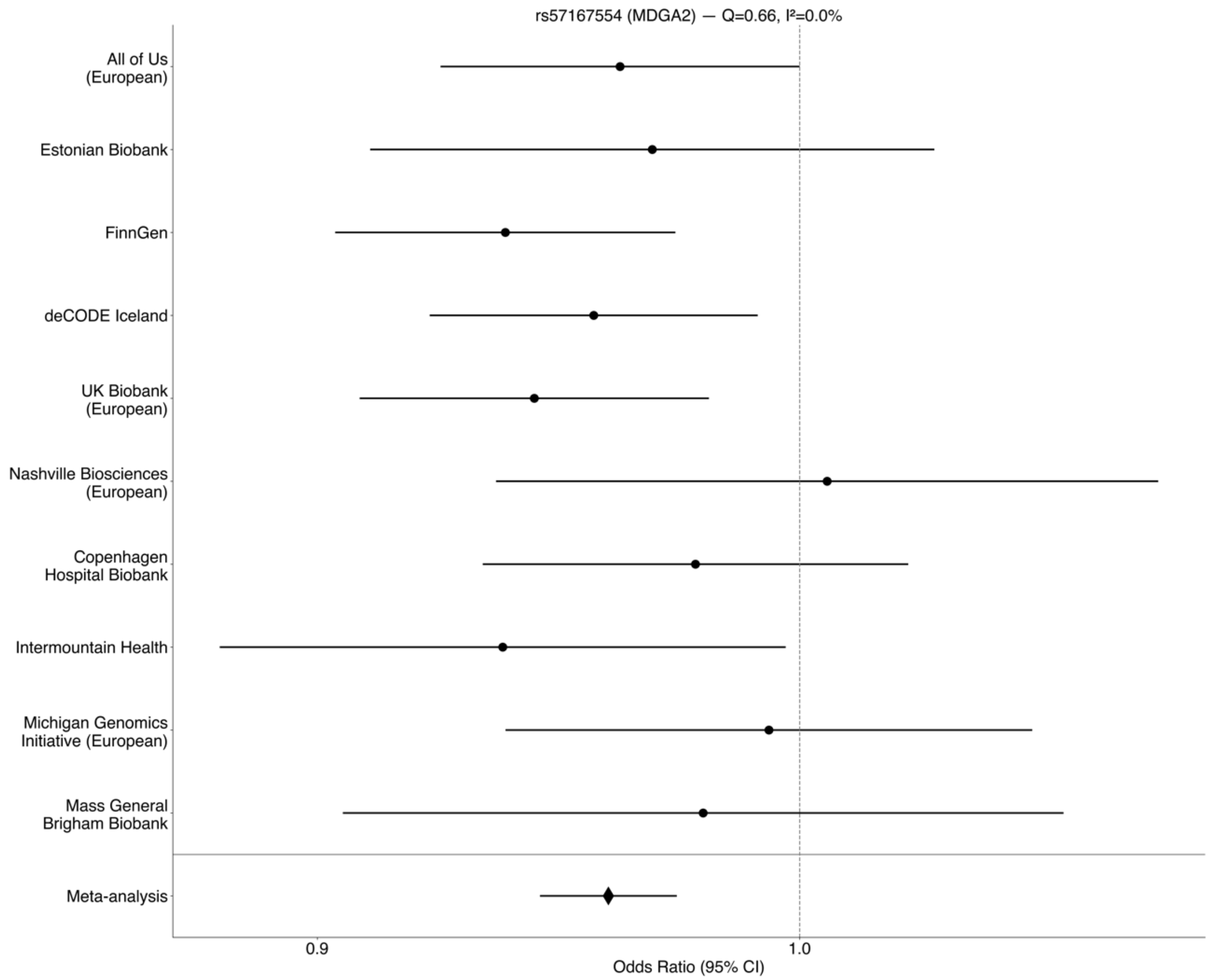
rs57167554 (MDGA2) — Q=0.66, I²=0.0%
All of Us (European)
Estonian Biobank
FinnGen
deCODE Iceland
UK Biobank (European)
Nashville Biosciences (European)
Copenhagen Hospital Biobank
Intermountain Health
Michigan Genomics Initiative (European)
Mass General Brigham Biobank
Meta-analysis
0.9
1.0
Odds Ratio (95% CI)

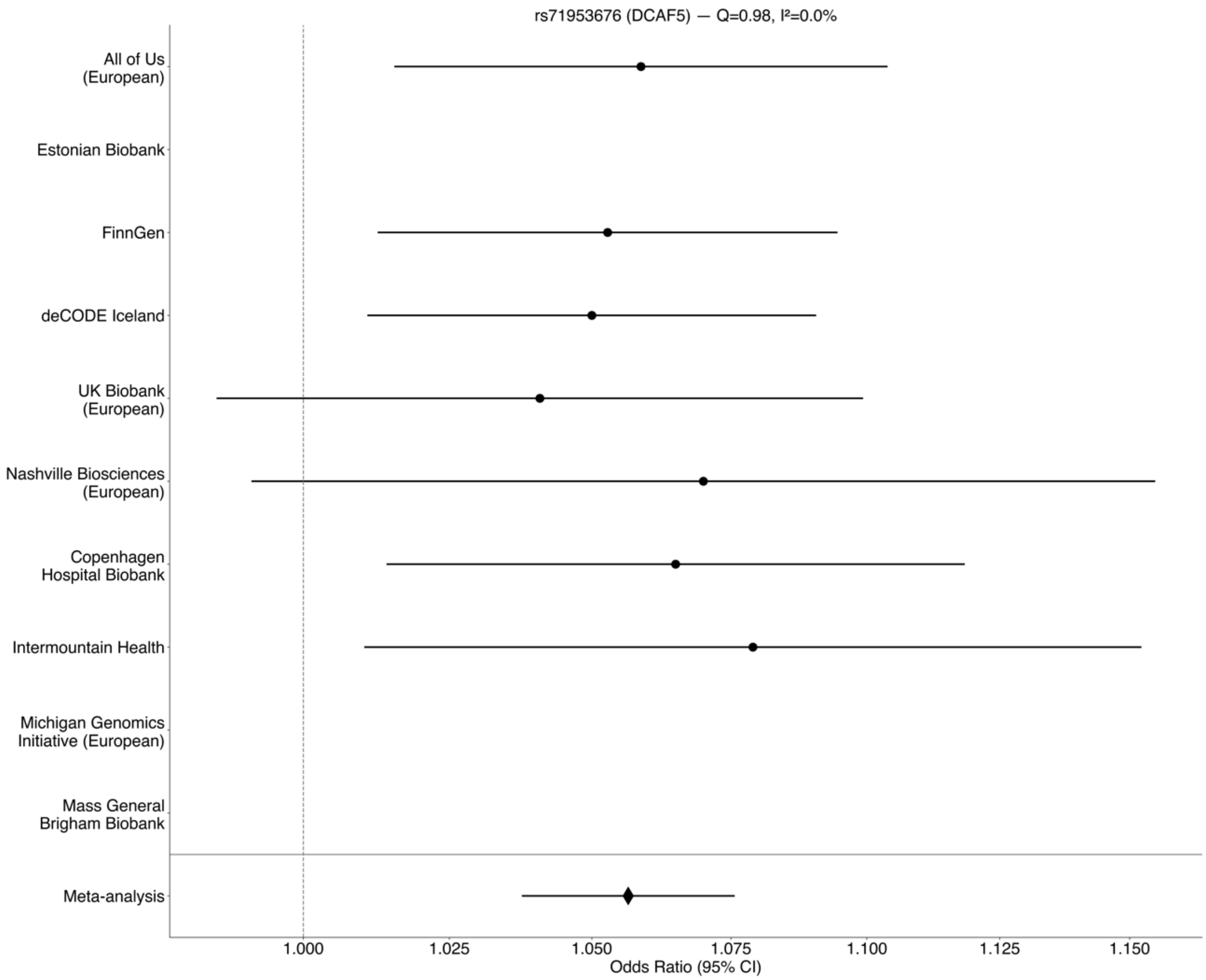
rs71953676 (DCAF5) — Q=0.98, I²=0.0%
All of Us (European)
Estonian Biobank
FinnGen
deCODE Iceland
UK Biobank (European)
Nashville Biosciences (European)
Copenhagen Hospital Biobank
Intermountain Health
Michigan Genomics Initiative (European)
Mass General Brigham Biobank
Meta-analysis
1.000
1.025
1.050
1.075
1.100
1.125
1.150
Odds Ratio (95% CI)

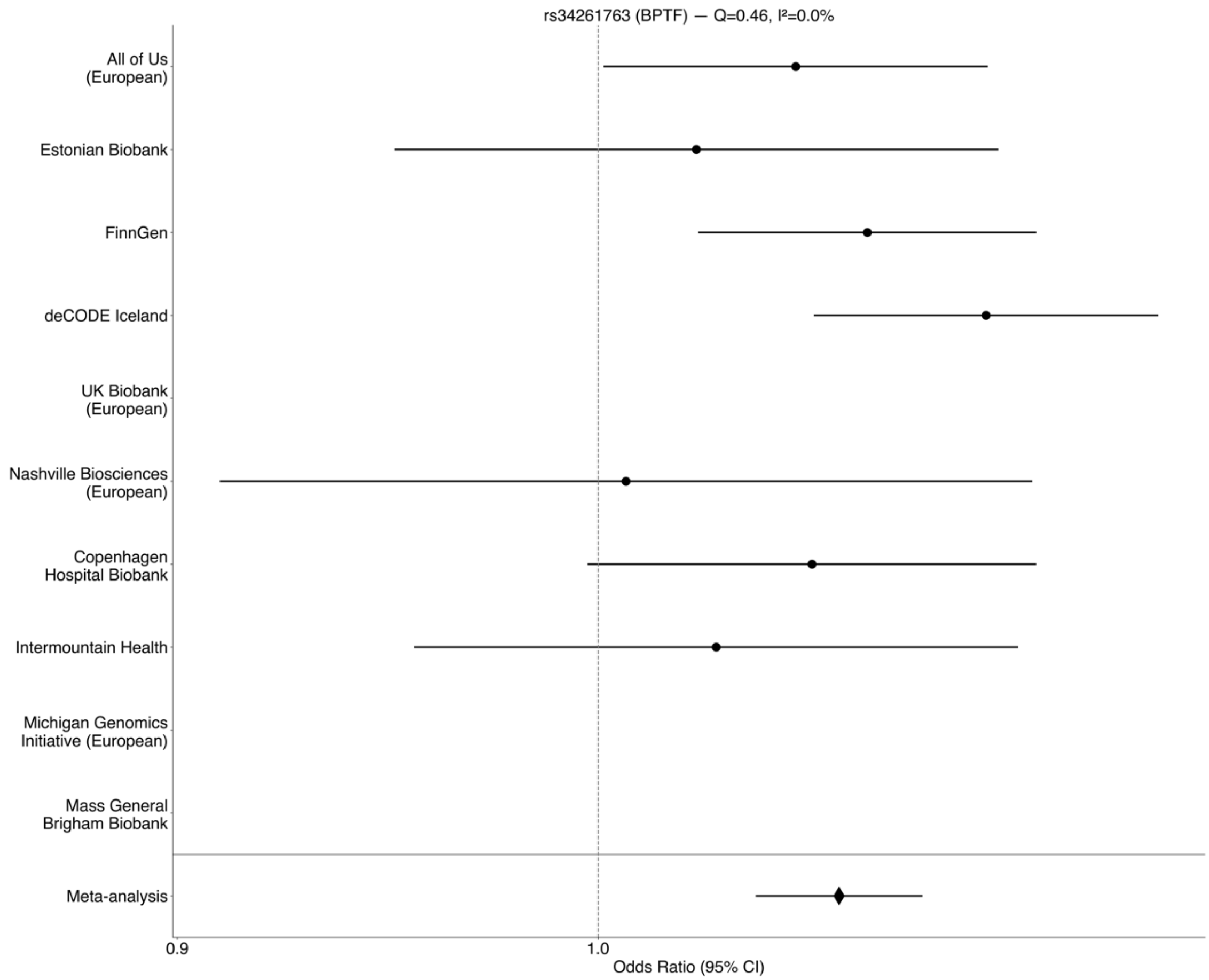
rs34261763 (BPTF) — Q=0.46, I²=0.0%
All of Us (European)
Estonian Biobank
FinnGen
deCODE Iceland
UK Biobank (European)
Nashville Biosciences (European)
Copenhagen Hospital Biobank
Intermountain Health
Michigan Genomics Initiative (European)
Mass General Brigham Biobank
Meta-analysis
0.9
1.0
Odds Ratio (95% CI)

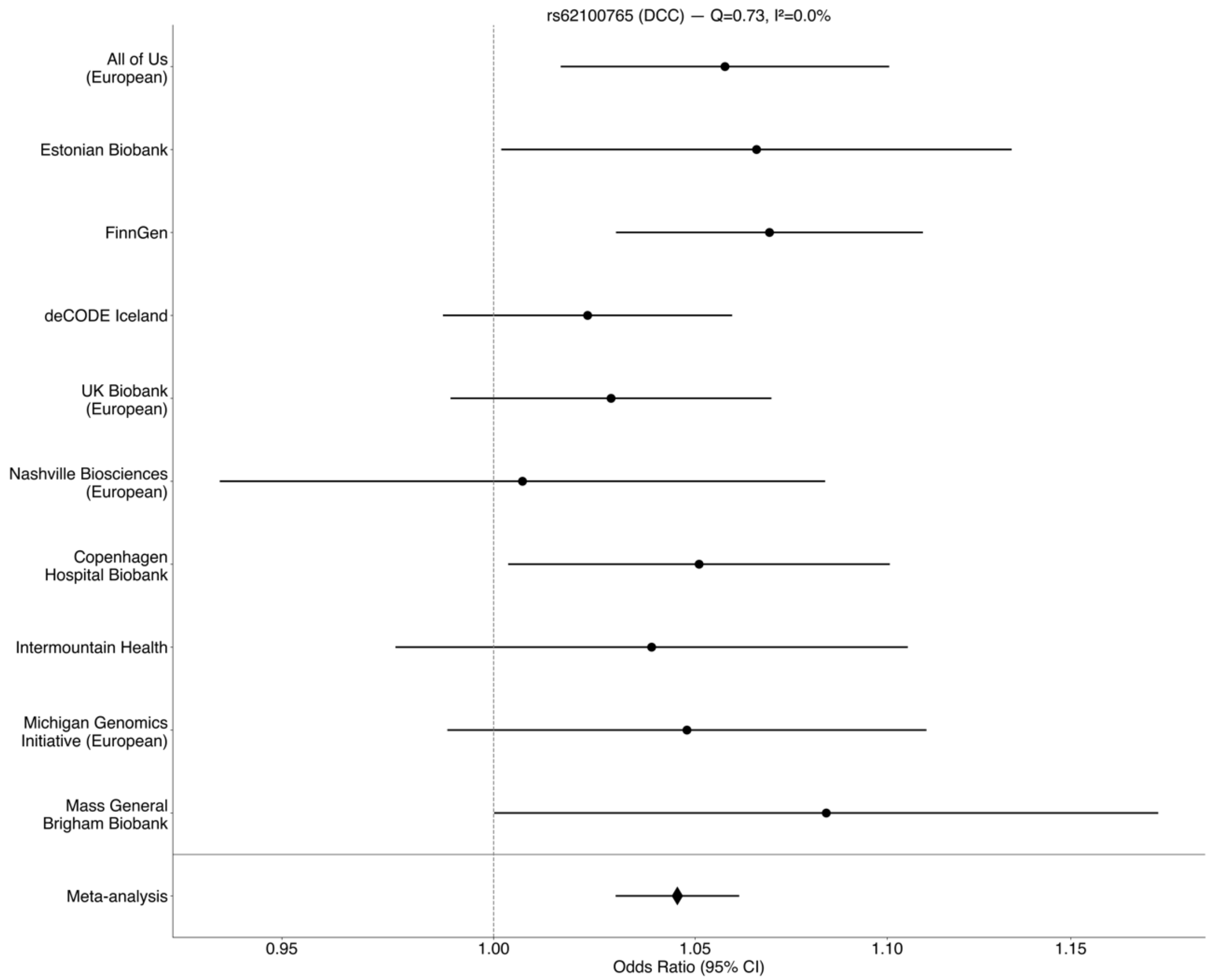
rs62100765 (DCC) — Q=0.73, I²=0.0%
All of Us
(European)
Estonian Biobank
FinnGen
deCODE Iceland
UK Biobank
(European)
Nashville Biosciences
(European)
Copenhagen
Hospital Biobank
Intermountain Health
Michigan Genomics
Initiative (European)
Mass General
Brigham Biobank
Meta-analysis
0.95
1.00
1.05
1.10
1.15
Odds Ratio (95% CI)

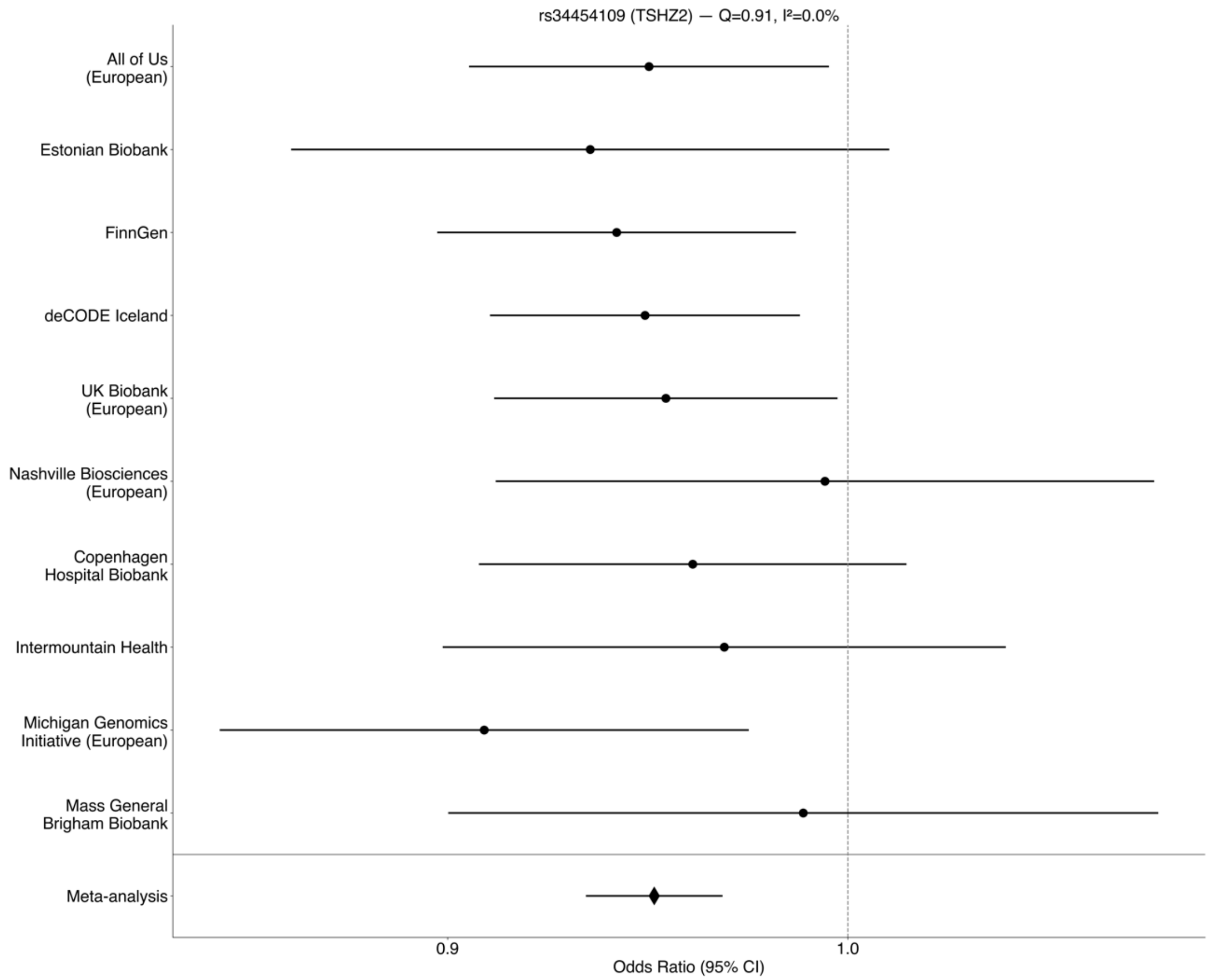
rs34454109 (TSHZ2) — Q=0.91, I²=0.0%
All of Us (European)
Estonian Biobank
FinnGen
deCODE Iceland
UK Biobank (European)
Nashville Biosciences (European)
Copenhagen Hospital Biobank
Intermountain Health
Michigan Genomics Initiative (European)
Mass General Brigham Biobank
Meta-analysis
0.9
1.0
Odds Ratio (95% CI)

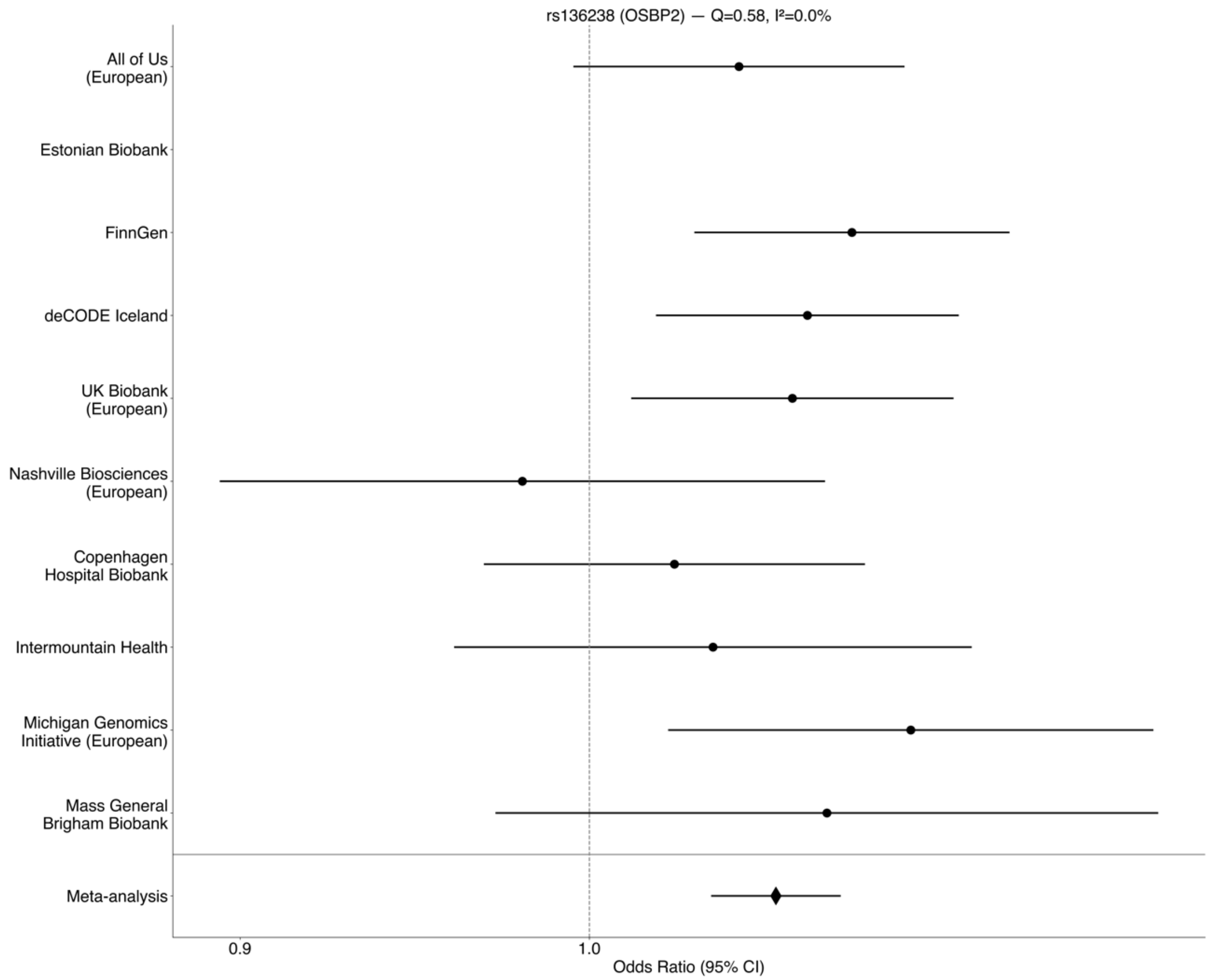

Figure S2: Forest plots of lead variants from the European-only, female-only meta-analysis. Effect estimates are plotted as odds ratios with 95% confidence intervals. "Copenhagen Hospital Biobank" is short for "Copenhagen Hospital Biobank and Danish Blood Donor Study".

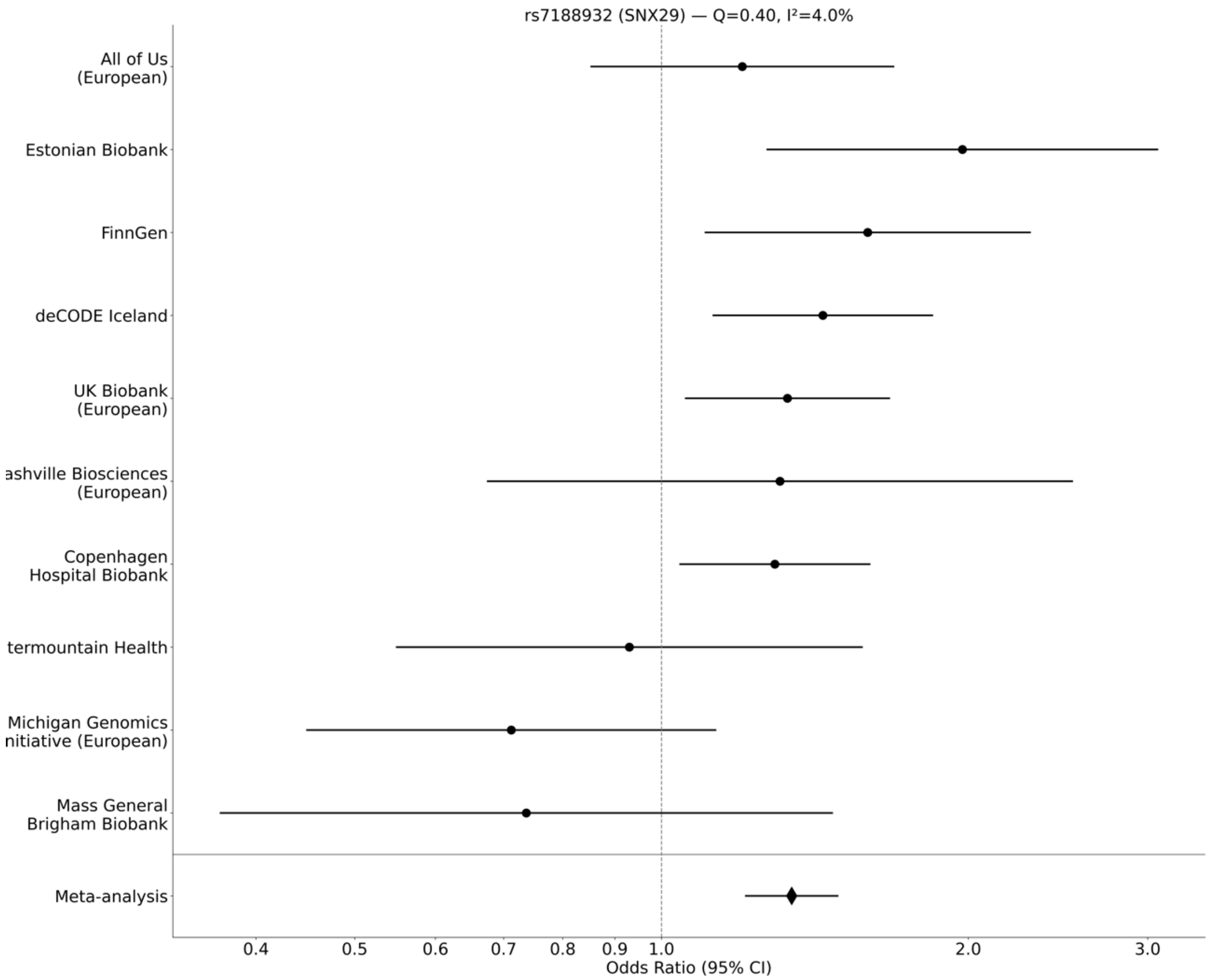

rs7188932 (SNX29) — Q=0.40, I²=4.0%
All of Us
(European)
Estonian Biobank
FinnGen
deCODE Iceland
UK Biobank
(European)
ashville Biosciences
(European)
Copenhagen
Hospital Biobank
termountain Health
Michigan Genomics
nitiative (European)
Mass General
Brigham Biobank
Meta-analysis
0.4
0.5
0.6
0.7
0.8
0.9
1.0
2.0
3.0
Odds Ratio (95% CI)

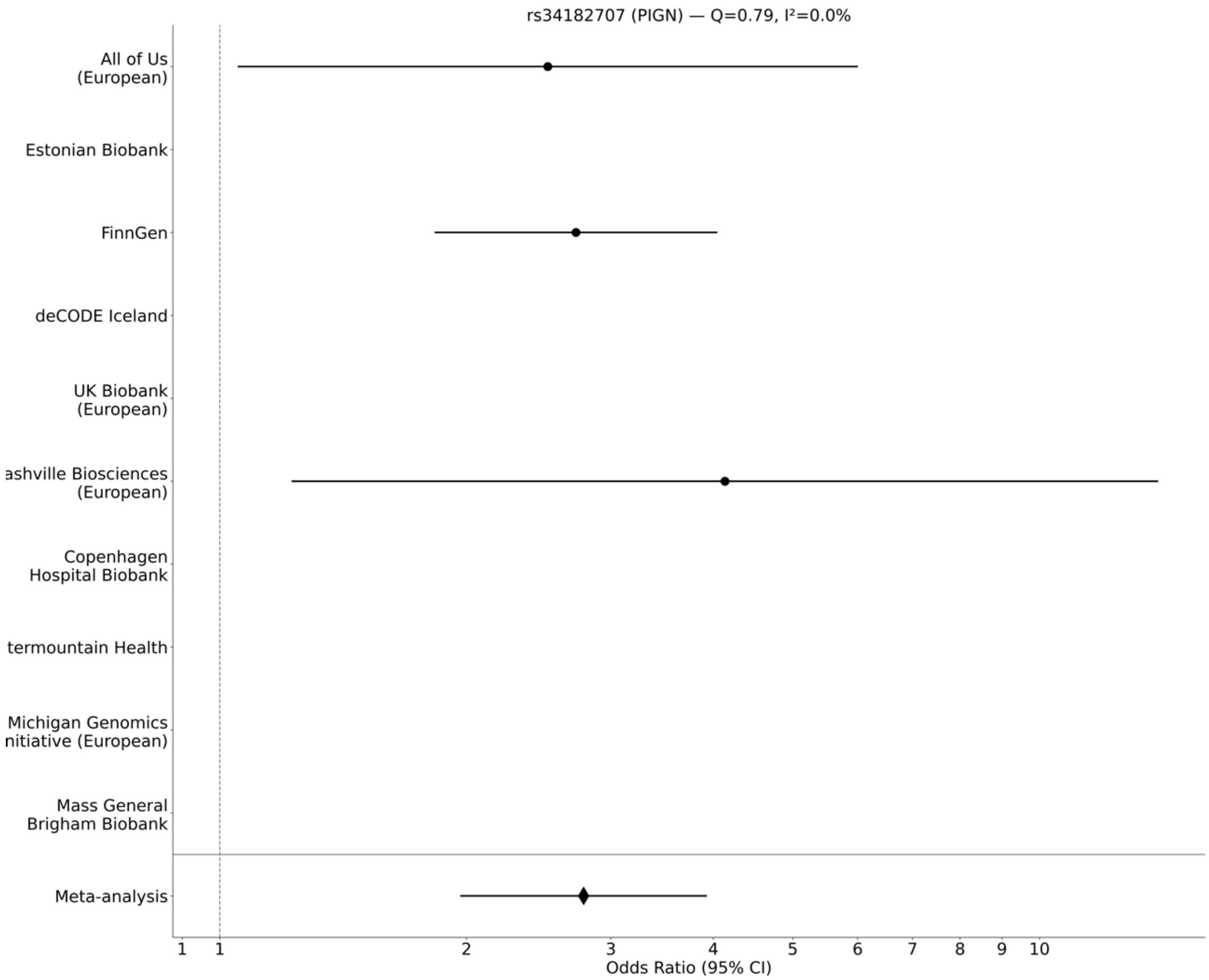

Figure S3: Forest plots of lead variants from the European-only, male-only meta-analysis. Effect estimates are plotted as odds ratios with 95% confidence intervals. “Copenhagen Hospital Biobank” is short for “Copenhagen Hospital Biobank and Danish Blood Donor Study”.
