# Supplementary material for: The genetic architecture of fibromyalgia across 2.5 million individuals
Source: Nat Med. 2026 Jul 28;32(8):3060–70. doi: 10.1038/s41591-026-04492-6 (PMC13472937; doi:10.1038/s41591-026-04492-6)
Supplement: Supplementary file 2 [file 41591_2026_4492_MOESM2_ESM.pdf]

nature portfolio

Corresponding author(s): Michael Wainberg

Last updated by author(s): May 8, 2026

Reporting Summary

Nature Portfolio wishes to improve the reproducibility of the work that we publish. This form provides structure for consistency and transparency in reporting. For further information on Nature Portfolio policies, see our Editorial Policies and the Editorial Policy Checklist.

Statistics

For all statistical analyses, confirm that the following items are present in the figure legend, table legend, main text, or Methods section.

n/a	Confirmed	
☐	☒	The exact sample size (*n*) for each experimental group/condition, given as a discrete number and unit of measurement
☐	☒	A statement on whether measurements were taken from distinct samples or whether the same sample was measured repeatedly
☐	☒	The statistical test(s) used AND whether they are one- or two-sided<br>*Only common tests should be described solely by name; describe more complex techniques in the Methods section.*
☐	☒	A description of all covariates tested
☐	☒	A description of any assumptions or corrections, such as tests of normality and adjustment for multiple comparisons
☐	☒	A full description of the statistical parameters including central tendency (e.g. means) or other basic estimates (e.g. regression coefficient) AND variation (e.g. standard deviation) or associated estimates of uncertainty (e.g. confidence intervals)
☐	☒	For null hypothesis testing, the test statistic (e.g. *F*, *t*, *r*) with confidence intervals, effect sizes, degrees of freedom and *P* value noted<br>*Give P values as exact values whenever suitable.*
☒	☐	For Bayesian analysis, information on the choice of priors and Markov chain Monte Carlo settings
☒	☐	For hierarchical and complex designs, identification of the appropriate level for tests and full reporting of outcomes
☒	☐	Estimates of effect sizes (e.g. Cohen's *d*, Pearson's *r*), indicating how they were calculated

Our web collection on statistics for biologists contains articles on many of the points above.

Software and code

Policy information about availability of computer code

Data collection	No custom methods were developed for data collection.
Data analysis	The following software were used in this study: PLINK v1.9.0 and v2.0.0, regenie v3.0.3, v3.2.8, v3.3 and v3.4.1, SAIGE v0.35, Illumina GenomeStudio v2.0.4, GenomeStudio 2.0 Genotyping Module v2.0.4 with GenTrain v3.0, zCall v3.4, Beagle v5.4 / beagle.22Jul22.46e.jar, Eagle v2.4, SHAPEIT4 v4.2.2, Minimac4, Michigan Imputation Server, Graphtyper v2.7.1, ADMIXTURE v1.23, KING v2.1.3, v2.2.7 and v2.3.0, FlashPCA2 v2.0, PCAone v0.3.4, Picard, LDSC v1.0.1, LDSC-SEG using LDSC v1.0.1, FLAMES v1.1.2, SuSiE/susieR v0.14.2, PoPS v0.2, MAGMA v1.10, Variant Effect Predictor, kallisto v0.46, PRS-CS v1.1.0, SomaLogic SOMAscan v4.

For manuscripts utilizing custom algorithms or software that are central to the research but not yet described in published literature, software must be made available to editors and reviewers. We strongly encourage code deposition in a community repository (e.g. GitHub). See the Nature Portfolio guidelines for submitting code & software for further information.

Data

Policy information about availability of data

All manuscripts must include a data availability statement. This statement should provide the following information, where applicable:

- Accession codes, unique identifiers, or web links for publicly available datasets
- A description of any restrictions on data availability
- For clinical datasets or third party data, please ensure that the statement adheres to our policy

GWAS summary statistics are available in the GWAS catalog (accession numbers GCST90838603 to GCST90838628) and polygenic risk scores are available in the PGS catalog (score IDs PGS012560 and PGS012561). All summary statistics and polygenic risk scores are also available at https://paingenomics.org.
This study used data from the All of Us Research Program's Controlled Tier Dataset Curated Data Repository version 8, available to authorized users on the Researcher Workbench.

Research involving human participants, their data, or biological material

Policy information about studies with human participants or human data. See also policy information about sex, gender (identity/presentation), and sexual orientation and race, ethnicity and racism.

Reporting on sex and gender	We conducted analysis in both sexes with sex as a covariate and we conducted sex-stratified analysis.
Reporting on race, ethnicity, or other socially relevant groupings	We report sample sizes per genetic ancestry. We do not report on race, ethnicity or other socially relevant groupings.
Population characteristics	We conducted multi-ancestry analysis including European, African, South Asian and Admixed American samples. We also conducted European only analysis. Our primary meta-analysis encompassed 54,629 fibromyalgia cases and 2,509,126 controls. 89.7% of cases were of European ancestry and 87.7% of cases were female. All participants were 18 years or older.
Recruitment	Relevant recruitment details for each cohort are described in the methods and executed as per ethics approvals.
Ethics oversight	All of Us (All of Us Institutional Review Board), UK Biobank (North West Multi-centre Research Ethics Committee / North West–Haydock Research Ethics Committee), FinnGen (Coordinating Ethics Committee of the Hospital District of Helsinki and Uusimaa), Estonian Biobank (Estonian Committee on Bioethics and Human Research, Estonian Ministry of Social Affairs), Genes & Health (London–South East Research Ethics Committee / NRES Committee London–South East), Mass General Brigham Biobank (Mass General Brigham Institutional Review Board / Mass General Brigham Human Research Committee), Michigan Genomics Initiative (University of Michigan Medical School Institutional Review Board), deCODE Genetics/Amgen Iceland (National Bioethics Committee of Iceland and Icelandic Data Protection Authority), Copenhagen Hospital Biobank (Danish National Committee on Health Research Ethics and Capital Region Data Protection Agency), Danish Blood Donor Study (Danish National Committee on Health Research Ethics and Danish Data Protection Agency), Intermountain Health (Intermountain Healthcare Institutional Review Board), and Nashville Biosciences/BioVU (Vanderbilt University Medical Center Institutional Review Board) gave ethical approval for this work. Participants were not compensated for this study.

Note that full information on the approval of the study protocol must also be provided in the manuscript.

Field-specific reporting

Please select the one below that is the best fit for your research. If you are not sure, read the appropriate sections before making your selection.

☒ Life sciences ☐ Behavioural & social sciences ☐ Ecological, evolutionary & environmental sciences

For a reference copy of the document with all sections, see nature.com/documents/nr-reporting-summary-flat.pdf

Life sciences study design

All studies must disclose on these points even when the disclosure is negative.

Sample size	The number of datasets was determined by the availability of cohorts with genome-wide genotype data, fibromyalgia phenotype definitions suitable for harmonized analysis, and permission to contribute cohort-level summary statistics to the meta-analysis. We sought to include all eligible datasets available through the participating cohorts and consortia, rather than selecting a fixed number of datasets based on a formal sample-size calculation.<br><br>The primary meta-analysis included 54,629 fibromyalgia cases and 2,509,126 controls across 11 cohorts. All cohort-specific sample sizes are reported in the Supplementary Tables. This dataset was considered sufficient as it provided statistical power for genome-wide discovery of common-variant associations.
Data exclusions	Cohort-specific exclusions are described in the methods. Generally, we excluded persons with sex-chromosome aneuploidy

Data exclusions	or that had discordant genetic sex versus self-reported sex, person that were not of European, African, South Asian or Admixed American genetic ancestry, and persons failing sample-level GWAS quality control.
Replication	Reproducibility of the genetic association findings was assessed by comparing effect estimates across contributing cohorts. For genome-wide significant loci, we examined cohort-specific effect directions and confidence intervals using forest plots, and evaluated between-cohort heterogeneity using standard heterogeneity statistics. For the primary association signals, the majority, and in several cases all, contributing cohorts showed concordant directions of effect, and there was no evidence of substantial heterogeneity.
Randomization	Randomization is not applicable to a GWAS study.
Blinding	Blinding is not applicable to a GWAS study.

Reporting for specific materials, systems and methods

We require information from authors about some types of materials, experimental systems and methods used in many studies. Here, indicate whether each material, system or method listed is relevant to your study. If you are not sure if a list item applies to your research, read the appropriate section before selecting a response.

Materials & experimental systems

n/a	Involved in the study
☒	☐ Antibodies
☒	☐ Eukaryotic cell lines
☒	☐ Palaeontology and archaeology
☒	☐ Animals and other organisms
☒	☐ Clinical data
☒	☐ Dual use research of concern
☒	☐ Plants

Methods

n/a	Involved in the study
☒	☐ ChIP-seq
☒	☐ Flow cytometry
☒	☐ MRI-based neuroimaging

Plants

Seed stocks	*Report on the source of all seed stocks or other plant material used. If applicable, state the seed stock centre and catalogue number. If plant specimens were collected from the field, describe the collection location, date and sampling procedures.*
Novel plant genotypes	*Describe the methods by which all novel plant genotypes were produced. This includes those generated by transgenic approaches, gene editing, chemical/radiation-based mutagenesis and hybridization. For transgenic lines, describe the transformation method, the number of independent lines analyzed and the generation upon which experiments were performed. For gene-edited lines, describe the editor used, the endogenous sequence targeted for editing, the targeting guide RNA sequence (if applicable) and how the editor was applied.*
Authentication	*Describe any authentication procedures for each seed stock used or novel genotype generated. Describe any experiments used to assess the effect of a mutation and, where applicable, how potential secondary effects (e.g. second site T-DNA insertions, mosiacism, off-target gene editing) were examined.*
